# Focal adhesion protein Kindlin-2 regulates bone homeostasis in mice

**DOI:** 10.1038/s41413-019-0073-8

**Published:** 2020-01-02

**Authors:** Huiling Cao, Qinnan Yan, Dong Wang, Yumei Lai, Bo Zhou, Qi Zhang, Wenfei Jin, Simin Lin, Yiming Lei, Liting Ma, Yuxi Guo, Yishu Wang, Yilin Wang, Xiaochun Bai, Chuanju Liu, Jian Q. Feng, Chuanyue Wu, Di Chen, Xu Cao, Guozhi Xiao

**Affiliations:** 1 Guangdong Provincial Key Laboratory of Cell Microenvironment and Disease Research, Shenzhen Key Laboratory of Cell Microenvironment, and Department of Biology, Southern University of Science and Technology, Shenzhen, 518055 China; 2 Key Laboratory of Neuroregeneration of Jiangsu and Ministry of Education, Co-innovation Center of Neuroregeneration, Nantong University, Nantong, 226001 China; 3 Department of Orthopedic Surgery, Rush University Medical Center, Chicago, IL 60612 USA; 4 Department of Cell Biology, School of Basic Medical Sciences, Southern Medical University, Guangzhou, 510515 China; 5 Department of Orthopedic Surgery, New York University School of Medicine, New York, NY 10003 USA; 6 Department of Cell Biology, New York University School of Medicine, New York, NY 10016 USA; 7 Department of Biomedical Sciences, Texas A&M University College of Dentistry, Dallas, TX 75246 USA; 8 Department of Orthopedic Surgery, The Johns Hopkins University, Baltimore, MD 21205 USA

**Keywords:** Bone, Calcium and phosphate metabolic disorders, Osteoporosis

## Abstract

Our recent studies demonstrate that the focal adhesion protein Kindlin-2 is critical for chondrogenesis and early skeletal development. Here, we show that deleting Kindlin-2 from osteoblasts using the 2.3-kb mouse *Col1a1-Cre* transgene minimally impacts bone mass in mice, but deleting Kindlin-2 using the 10-kb mouse *Dmp1-Cre* transgene, which targets osteocytes and mature osteoblasts, results in striking osteopenia in mice. Kindlin-2 loss reduces the osteoblastic population but increases the osteoclastic and adipocytic populations in the bone microenvironment. Kindlin-2 loss upregulates sclerostin in osteocytes, downregulates β-catenin in osteoblasts, and inhibits osteoblast formation and differentiation in vitro and in vivo. Upregulation of β-catenin in the mutant cells reverses the osteopenia induced by Kindlin-2 deficiency. Kindlin-2 loss additionally increases the expression of RANKL in osteocytes and increases osteoclast formation and bone resorption. Kindlin-2 deletion in osteocytes promotes osteoclast formation in osteocyte/bone marrow monocyte cocultures, which is significantly blocked by an anti-RANKL-neutralizing antibody. Finally, Kindlin-2 loss increases osteocyte apoptosis and impairs osteocyte spreading and dendrite formation. Thus, we demonstrate an important role of Kindlin-2 in the regulation of bone homeostasis and provide a potential target for the treatment of metabolic bone diseases.

## Introduction

Adult bone is a dynamic tissue that constantly adapts its structure through bone remodeling. Osteocytes originate from osteoprogenitors through osteoblast differentiation and are the most abundant bone cells. It is now widely believed that osteocytes play key roles in the regulation of bone remodeling and in the mediation of bone mechanotransduction, although the mechanisms are currently incompletely defined.^1–10^ Elucidating these mechanisms will advance the progress of bone biology research and help define new targets for the treatment of metabolic bone diseases.

Osteocytes coordinate bone remodeling, during which osteoclast-mediated bone resorption is closely coupled with osteoblast-mediated bone formation.^1–5,8,11^ Osteocytes communicate with both osteoblasts and osteoclasts located on bone surfaces through their dendrites. Furthermore, osteocytes regulate the formation and function of osteoblasts and osteoclasts by producing key mediators, such as proteins and miRNAs, through local diffusion, circulation, or exosomes. Among those, sclerostin is encoded by the *SOST* gene and is almost exclusively produced by osteocytes.^12^ Sclerostin interacts with the Wnt coreceptors Lrp5 and Lrp6 and suppresses Wnt/β-catenin signaling, which is the main determinant of osteoblast formation and bone mass accrual.^13^ Romosozumab (AMG 785), a humanized monoclonal antibody that targets human sclerostin, significantly increased bone mass and reduced the risk for vertebral fractures in women with postmenopausal osteoporosis.^14^ The receptor activator of nuclear factor kappaB ligand (RANKL),^9,10^ a master regulator of osteoclast formation and differentiation, and osteoprotegerin, a potent inhibitor of RANKL, are now known to be primarily produced by osteocytes.^15^ However, key signals that modulate the expression of those factors in osteocytes remain poorly defined.

Through integrin activation, Kindlins play a pivotal role in the regulation of cell differentiation, adhesion, migration, and signaling.^16–21^ Mammalian cells have three Kindlin proteins, i.e., Kindlin-1, -2, and -3. They are encoded by three different genes, Kindlin-1 by Fermt1, Kindlin-2 by Fermt2, and Kindlin-3 by Fermt3. Human genetic diseases are linked to mutations in *FERMT1* and *FERMT3*, but not *FERMT2*, genes.^22–27^ *Kindlin-2* knockout mice died at E7.5.^28^ For this reason, we conditionally deleted Kindlin-2 expression in Prx1-expressing mesenchymal stem cells and found that Kindlin-2 regulates chondrogenesis and early skeletal development by modulating TGF-β signaling and Sox9 expression in chondrocytes and their precursors.^29^ We further demonstrated that Kindlin-2 determines whether mesenchymal stem cells differentiate into osteoblasts or adipocytes through control of YAP1/TAZ.^30^ However, the potential role(s) of Kindlin-2 in the regulation of bone homeostasis have not been established.

Through comprehensive analyses of cells and genetic mouse models in this study, we define a critical new role of Kindlin-2. Its expression in osteocytes and mature osteoblasts regulates bone homeostasis by controlling bone remodeling through distinct mechanisms.

## Results

### Deleting Kindlin-2 in osteoblasts using the 2.3-kb mouse *Col1a1-Cre* transgene slightly reduces bone mass in mice

Our previous studies demonstrated an essential role of Kindlin-2 in chondrogenesis and skeletogenesis.^29^ To determine the potential role of Kindlin-2 in the osteoblastic cell lineage, we first deleted its expression in osteoblasts by breeding 2.3-kb mouse collagen type I, alpha 1(*Col1a1*)-*Cre* mice with *Kindlin-2*^*fl/fl*^*;* mice and created *Kindlin-2*^*fl/fl*^*; Col1a1-Cre* conditional knockout mice (hereafter referred to as *Kind2-Col1a1*). Microcomputed tomography (μCT) analysis of distal femurs revealed that all key parameters, including bone volume/tissue volume fraction (BV/TV), trabecular number (Tb.N), trabecular separation (Tb.Sp), trabecular thickness (Tb.Th), cortical thickness (Cort.Th), and bone mineral density (BMD), were not significantly altered in 2-month-old *Kind2-Col1a1* mice compared with their control littermates (Supplementary Fig. 1a–f). However, at 4 months after birth, *Kind2-Col1a1* displayed a decrease in BV/TV, but not other parameters, compared with their sex-matched control littermates (Supplementary Fig. 1g–j).

### Mice lacking Kindlin-2 in mature osteoblasts and osteocytes display striking osteopenia

**Fig. 1:**
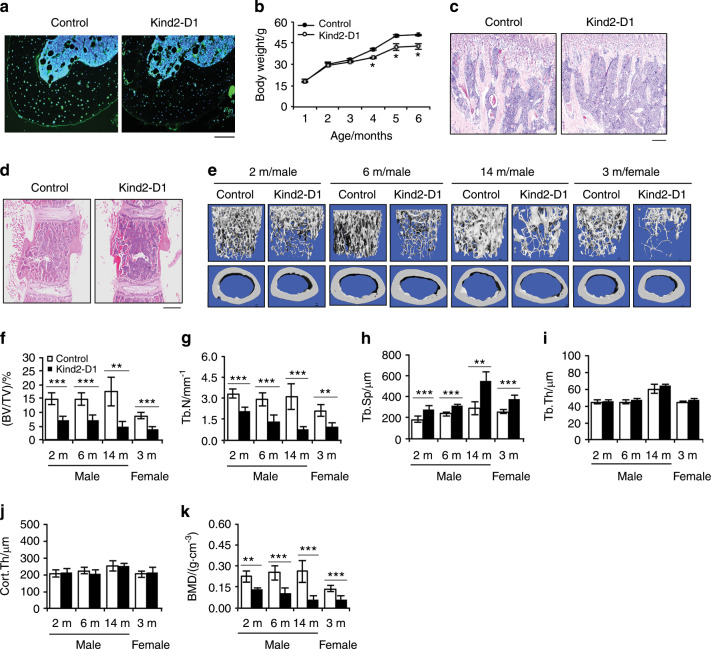
Deleting Kindlin-2 in osteocytes and mature osteoblasts causes striking osteopenia in young and rapidly growing, adult, and aging mice. **a** Immunofluorescence staining of tibial sections of 4-month-old male *Kind2-D1* mice and control littermates with an anti-Kindlin-2 antibody. Scale bar, 80 μm. **b** Animal growth curves. *N* = 2–6 male mice per group. *P < 0.05, versus control, Student’s *t* test. The results are expressed as the mean ± standard deviation (s.d.). Hematoxylin and eosin staining of tibial (**d**) or lumbar spine (L4) (**c**) sections from 2-month-old male *Kind2-D1* mice and their control littermates. Scale bar: 160 μm (**c**) or 400 μm (**d**). **e** Three-dimensional (3D) reconstruction from microcomputed tomography (μCT) scans of the distal femurs of *Kind2-D1* mice and their control littermates with the indicated ages and sexes. **f**–**k** Quantitative analyses of bone volume/tissue volume (BV/TV), trabecular number (Tb.N), trabecular thickness (Tb.Th), trabecular separation (Tb.Sp), cortical thickness (Cort.Th), and bone mineral density (BMD) of distal femurs of *Kind2-D1* mice and their control littermates with the indicated ages and sexes. *N* = 4, 14-month-old male *Kind2-D1* mice; *N* = 6, 14-month-old male control *Kind2-D1* mice; *N* = 9, 6-month-old male control mice; *N* = 7 mice in the remaining groups. *P < 0.05, **P < 0.01, ***P < 0.001, versus controls, Student’s *t* test. The results are expressed as the mean ± s.d.

Given the subtle osteopenic phenotype of the *Kind2-Col1a1* mice observed above, we wondered whether Kindlin-2 plays a more important role in mature osteoblasts and osteocytes. To test if this is the case, we next deleted Kindlin-2 by breeding *Kindlin-2*^*fl/fl*^ mice with 10-kb mouse dentin matrix protein 1 (*Dmp1*)*-*Cre transgenic mice^31^ and obtained *Kindlin-2*^*fl/fl*^*; Dmp1-Cre* mice (referred to as *Kind2-D1* hereafter), in which Kindlin-2 is selectively deleted in Dmp1-positive cells, i.e., primarily osteocytes and mature osteoblasts. As demonstrated by immunofluorescence (IF) staining, Kindlin-2 protein was strongly detected in cortical osteocytes of control mice, which was dramatically reduced in *Kind2-D1* osteocytes (Fig. 1a). *Kind2-D1* were born at a frequency expected by Mendelian law and, at birth, were indistinguishable from their control littermates. Beginning 4 months after birth, *Kind2-D1* displayed slightly reduced body weight (Fig. 1b). At 2 months of age, *Kind2-D1* exhibited markedly decreased trabecular bone mass in the tibiae and lumbar spine (L4) compared with control mice (Fig. 1c, d). Micro-CT analysis of distal femurs showed a dramatic reduction in trabecular bone mass in 6-month-old male *Kind2-D1* compared with controls (Supplementary Fig. 2). The BV/TV of distal femurs in male *Kind2-D1* mice was reduced by 52% at 2 months of age, 50% at 6 months of age, and 80% at 14 months of age, while it was reduced by 59% in 3-month-old female *Kind2-D1* mice compared with their respective controls (Fig. 1e, f). *Kind2-D1* displayed dramatically decreased Tb.N and BMD and increased Tb.Sp with no markedly altered Tb.Th or Cort.Th (Fig. 1g-k). The littermates generated from breeding, including the *Kindlin-2* flox heterozygotes that harbor *Dmp1-Cre* (i.e., *Kindlin-2*^*fl/+*^*; Dmp1-Cre*), *Kindlin-2*^*fl/fl*^ or *Dmp1-Cre*, did not display marked skeletal abnormalities (data not shown) and were used as control groups as specified in each experiment.

### Kindlin-2 loss decreases osteoblast number and impairs osteoblast function

**Fig. 2:**
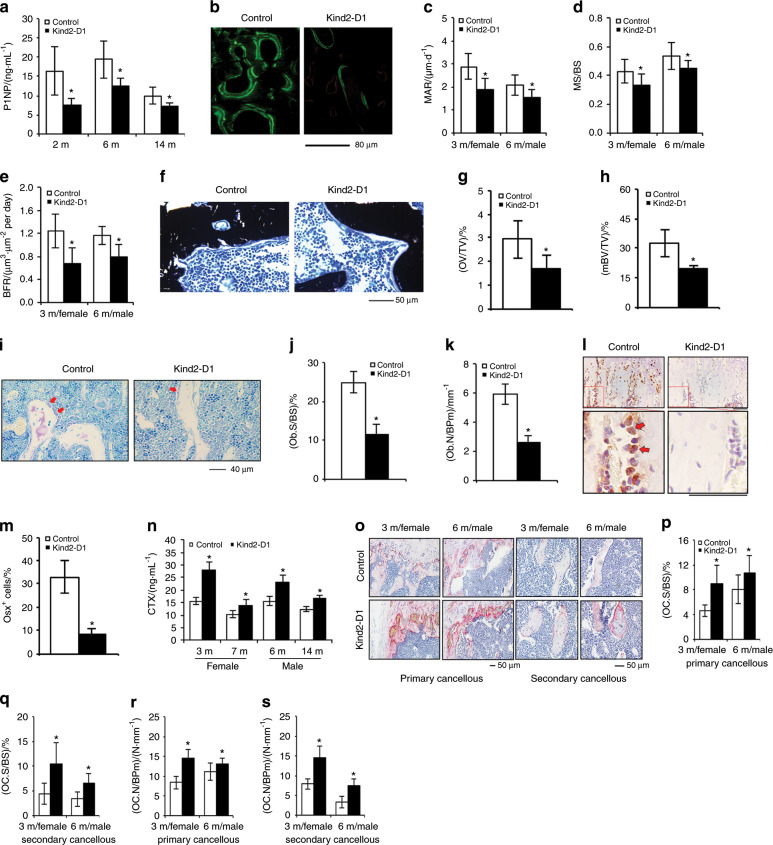
Abnormal bone remodeling in *Kind2-D1* mice. **a** Serum levels of procollagen type 1 amino-terminal propeptide (P1NP), *N* = 8 male mice per group. *P < 0.05, versus controls, Student’s *t* test. The results are expressed as the mean ± standard deviation (s.d.). **b**–**e** Calcein double labeling. Representative images of 6-month-old male *Kind2-D1* and control femoral sections (**b**). Scale bar, 80 μm. Sections of nondemineralized femurs of 3-month-old female and 6-month-old male *Kind2-D1* and control littermates were used for measurements of mineral apposition rate (MAR) (**c**), mineralizing surface per bone surface (MS/BS) (**d**), and bone formation rate (BFR) (**e**). Quantitative MAR, MS/BS, and BFR data for metaphyseal trabecular bones. *N* = 9 in the 7-month-old control male group; *N* = 8 in the remaining groups. *P < 0.05, versus controls, Student’s *t* test. The results are expressed as the mean ± s.d. (**f**–**h**) von Kossa staining. Undecalcified sections of femora from 3-month-old female *Kind2-D1* mice and control littermates were subjected to von Kossa staining. *N* = 5 mice per genotype. *P < 0.05, versus controls, Student’s *t* test. Scale bar, 50 μm. **i**–**k** Toluidine blue staining of tibial sections of 2-month-old male *Kind2-D1* mice and their control littermates. *N* = 8 control mice; *N* = 7 *Kind2-D1* mice. *P < 0.05, versus controls, Student’s *t* test. Red arrowheads indicate osteoblasts located on the cancellous bone surfaces. Scale bar, 40 μm. **l**, **m** Immunohistochemical (IHC) staining of tibial sections of 2-month-old male *Kind2-D1* mice and their control littermates with an anti-osterix (Osx) antibody. Scale bar, 40 μm. Red arrowheads indicate Osx-positive cells located on the cancellous bone surfaces. Quantitative data of Osx-positive cells located on the cancellous bone surfaces (**m**). *N* = 7 control mice; *N* = 9 *Kind2-D1* mice. *P < 0.05, versus controls, Student’s *t* test. The results are expressed as the mean ± s.d. **n** Serum level of collagen type I cross-linked C-telopeptide (CTX). *N* = 6 mice per group. *P* < 0.05, versus control, Student’s *t* test. The results are expressed as the mean ± standard deviation (s.d.). **o**–**s** Tartrate-resistant acid phosphatase (TRAP) staining. Osteoclast surface/bone surface (Oc.S/BS) (**p**, **q**) and osteoclast number/bone perimeter (Oc.N/BPm) (**r**, **s**) of primary and secondary cancellous bones were measured using Image-Pro Plus 7.0. Scale bar, 50 μm. *N* = 8 mice per group. *P* < 0.05, versus control, Student’s *t* test. The results are expressed as the mean ± standard deviation (s.d.).

We determined whether osteoblast function was impaired in *Kind2-D1* mice by measuring the level of serum procollagen type 1 amino-terminal propeptide (P1NP), a serum indicator of osteoblast function and bone formation, using ELISA and observed significant reductions in serum P1NP in 2-, 6-, and 14-month-old male *Kind2-D1* relative to controls (Fig. 2a). We performed calcein double labeling experiments to measure the in vivo bone-forming activity by osteoblasts and found significant decreases in mineral apposition rate (MAR), mineralizing surface per bone surface (MS/BS), and bone formation rate (BFR) in the femoral metaphyseal cancellous bones in 3- and 6-month-old *Kind2-D1* compared with their control littermates (Fig. 2b–e). Notably, Kindlin-2 loss only slightly reduced BFR, but not MAR and MS/BS, in femoral diaphyseal cortical bones (Supplementary Fig. 3). The results of von Kossa staining of undecalcified femoral sections revealed significant reductions in osteoid volume/tissue volume and mineralized BV/TV of the cancellous bones of *Kind2-D1* mice compared with control littermates (Fig. 2f–h). The results of toluidine blue staining showed that the osteoblast surface/bone surface and osteoblast number/bone perimeter of metaphyseal trabecular bone were dramatically decreased in 2-month-old male *Kind2-D1* mice compared with their control littermates (Fig. 2i–k). The results of immunohistochemical staining showed that the number of Osx-expressing cells (i.e., preosteoblasts) located on the tibial metaphyseal cancellous bone surface was drastically decreased in *Kind2-D1* relative to that in the control group (Fig. 2l, m).

### Kindlin-2 loss promotes osteoclast formation and bone resorption

In addition to osteoblast-mediated bone formation, osteoclast-mediated bone resorption also plays an important role in the control of bone mass. We further determined whether Kindlin-2 loss affects osteoclast formation and bone resorption. We found that the serum levels of collagen type I cross-linked C-telopeptide (CTX), an in vivo indicator of osteoclast-mediated bone-resorbing activity, were significantly elevated in *Kind2-D1* of the indicated ages and sexes compared with control littermates (Fig. 2n). We further performed tartrate-resistant acid phosphatase (TRAP) staining of tibial sections of the two genotypes (Fig. 2o) and found that the osteoclast surface/bone surface and osteoclast number/bone perimeter were significantly increased in both primary and secondary trabecular bones in 3-month-old female and 6-month-old male *Kind2-D1* compared with control mice (Fig. 2p–s). Thus, increased bone-resorbing activity could contribute to osteopenia in *Kind2-D1*.

### Kindlin-2 loss decreases osteoblasts but increases osteoclasts

**Fig. 3:**
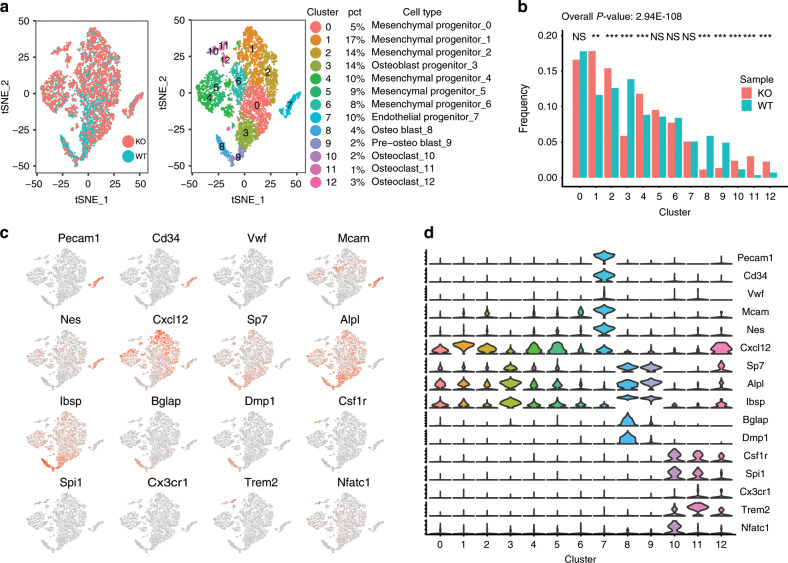
Kindlin-2 loss reduces the osteoblast population but increases the osteoclast population. **a** Single cell RNA sequencing data of the control and *Kind2-D1* groups were merged, showing no marked batch effect (left). Cells were classified into 13 clusters (right). Four major cell types, including mesenchymal progenitors, endothelial progenitors, osteoblasts, and osteoclasts, were identified. The corresponding percentage of cells in each cluster is indicated. **b** The distribution of different cell types was significantly changed in the *Kind2-D1* group (*P* value = 2.94E−108). Several clusters, including mesenchymal progenitors, osteoblasts, and osteoclasts, were significantly changed. *P* values were calculated with Pearson’s Chi-square test in R (version 3.5.2). **c** TSNEPlot of the merged data with selected marker genes. Red points indicate expression of the gene, and gray points indicate no expression. **d** Violin plot of the merged data with selected marker genes. The Y-axis indicates the expression level.

To explore whether Kindlin-2 loss alters the bone marrow microenvironment, we next analyzed the distribution of adhesive bone marrow cells by performing single-cell RNA sequencing analyses. Primary bone marrow cells from 3-month-old control and *Kind2-D1* mice were cultured under undifferentiating conditions to obtain enough cells for single-cell sequencing analysis, as described in section ‘Methods''. Four major cell types, namely, mesenchymal progenitor cells (clusters 0, 1, 2, 4, 5, and 6), endothelial progenitor cells (cluster 7), osteoblasts (clusters 3, 8, and 9), and osteoclasts (clusters 10, 11, and 12), were identified in two groups (Fig. 3a and Supplementary Fig. 4a). Mesenchymal progenitor cells were identified by the expression of *Mcam*, *Nes*, and *Cxcl12*; endothelial progenitor cells, by the expression of *Pecam1*, *Cd34*, and *Vwf*; osteoblasts by the expression of *Sp7*, *Alpl*, *Ibsp*, *Bglap*, and *Dmp1*; and osteoclasts, by the expression of *Csf1r*, *Spi1*, *Cx3cr1*, *Trem2*, and *Nfatc1* (Fig. 3c, d and Supplementary Fig. 4c, d).^32–34^ Furthermore, osteoblastic clusters were further recognized as osteoblast progenitors (cluster 3), preosteoblasts (cluster 9), and osteoblasts (cluster 8) according to their relative expression levels of *Sp7*, *Alpl*, *Ibsp*, *Bglap*, and *Dmp1*. Notably, adipocytes and adipocyte progenitors were not identified in either genotype (Supplementary Fig. 5).^35^ Of the 8 046 total cells, 5 849 were mesenchymal progenitor cells (72.70%), 409 were endothelial progenitor cells (5.08%), 1 418 were osteoblasts (17.62%), and 370 were osteoclasts (4.60%).

Pearson’s Chi-squared test was performed, and the distribution of cell types between the control and *Kind2-D1* groups was significantly different (*P* value = 2.94E−108) (Fig. 3b). Specifically, the frequencies of clusters 1, 2, 3, 4, 8, 9, 10, 11, and 12 were all significantly different between the control and *Kind2-D1* groups. Interestingly, all osteoblasts (clusters 3, 8, and 9) were significantly decreased in the *Kind2-D1* group compared with the control group. Consistent with the reduced numbers in osteoblasts and osteoprogenitors in *Kind2-D1* mice (Fig. 2i–m), the frequencies of osteoblast progenitors (13.87%), preosteoblasts (4.94%), and osteoblasts (5.90%) in bone marrow cell cultures of the control group were dramatically decreased to 5.88%, 1.35%, and 1.15%, respectively, in the *Kind2-D1* group. Consistent with increased osteoclast formation in *Kind2-D1* bone (Fig. 2o–s), the frequencies of the three osteoclast clusters [clusters 10 (1.19%), 11 (0.35%), and 12 (0.72%)] in bone marrow cultures of the control group were significantly increased to 2.38%, 3.01%, and 2.26%, respectively, in the *Kind2-D1* group. The alterations in osteoblast and osteoclast frequencies between the two groups were specific because no significant differences in endothelial progenitor clusters between the two groups were observed.

**Fig. 4:**
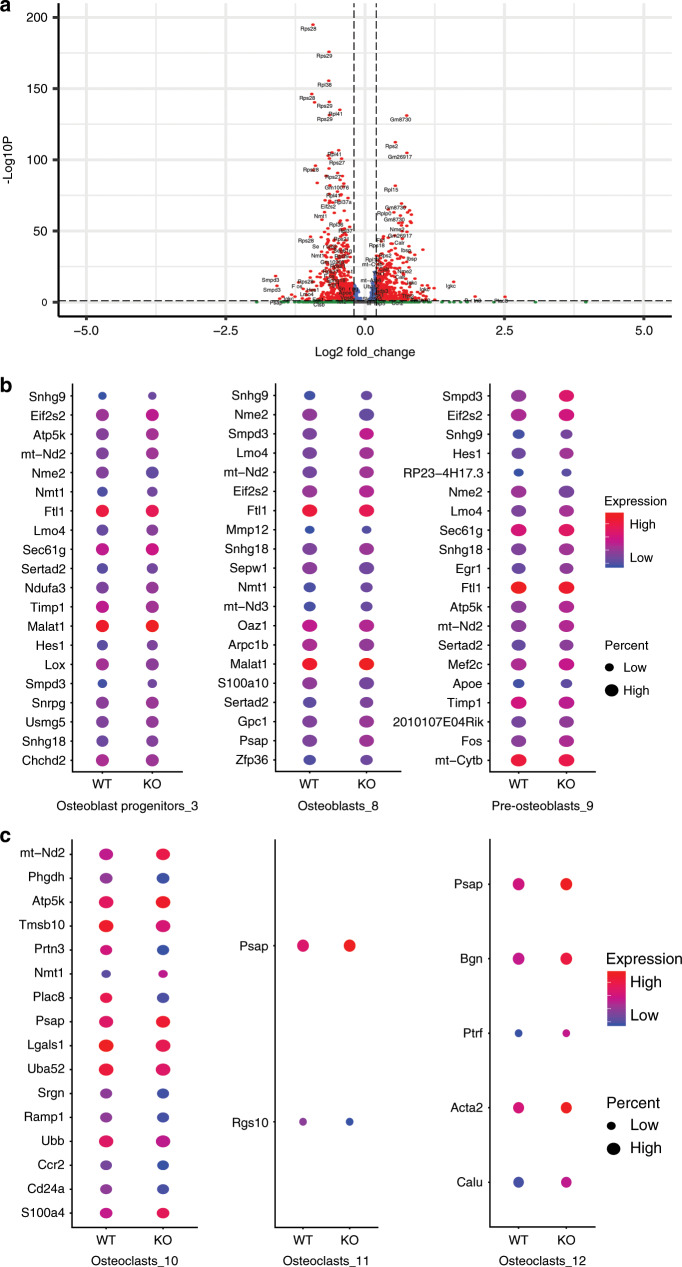
Kindlin-2 loss reduces the osteoblast population but increases the osteoclast population. **a** Volcano plot of differential gene expression results of all clusters between the control and *Kind2-D1* groups. The adjusted *P* value was used, the threshold for the adjusted *P* value was 0.01, and the absolute log2 fold change was 0.2. **b**, **c** Top 20 significant nonribosomal protein genes with adjusted *P* values lower than 0.01 of all three clusters of osteoblasts (**b**) and osteoclasts (**c**). The color indicates the average expression level of the corresponding genes in the group, and the diameter of the circle is correlated with the percentage of cells that express the gene in the group. WT: control group, KO: *Kind2-D1* group.

Over 1 000 genes were found to be significantly differentially expressed between the control and *Kind2-D1* groups, including 996 genes with adjusted *P* values lower than 0.01 (Supplementary Table 1). The top significant genes were mostly ribosomal protein-related genes (Fig. 4a), indicating that the protein translation rate might be different between the two groups. Because both osteoblasts and osteoclasts were influenced in *Kind2-D1* bones (Fig. 2), we were particularly interested in genes that were differentially expressed among clusters of osteoblasts and osteoclasts of the two genotypes. We initially focused on the top 20 most significant nonribosomal protein genes with adjusted *P* values lower than 0.01 (Fig. 4b, c). Among these genes, eight genes (*Snhg9*, *Eif2s2*, *mt-Nd2*, *Nme2*, *Ftl1*, *Lmo4*, *Sertad2*, and *Smpd3*) were differentially expressed in all three osteoblast clusters. In addition, *Atp5k* and *Snhg18* were differentially expressed in all three osteoblast clusters with adjusted *P* values lower than 0.01. Furthermore, in the osteoblast progenitor cluster and the pre-osteoblast cluster, *Apoe*, *Fos*, *Hes1*, *Mef2c*, *Sec61g*, and *Timp1* were differentially expressed with adjusted *P* values lower than 0.01. Several other genes that are known to be involved in bone development, including *Bmp1*, *Cxcl12*, *Dpm3*, *Fos*, *Itm2a*, *Ogn*, *Spp1*, and *Wisp1*, were differentially expressed only in cluster 3 (osteoblast progenitors) with adjusted *P* values lower than 0.01 between the control and *Kind2-D1* groups. The 996 genes were then subjected to gene set enrichment analysis using Metascape for each cluster (Supplementary Fig. 5). In the KEGG pathway, ribosome (mmu03010) was the most significantly enriched in almost all clusters, except osteoblast progenitors (cluster 3). For osteoblast progenitors (cluster 3), cytoplasmic translation (GO:0002181) was the most significantly enriched. The enrichment results suggest that protein translation might be largely different between the control and *Kind2-D1* groups.

### Mesenchymal stromal cells from *Kind2-D1* mice display reduced osteogenic but enhanced adipogenic differentiation capacity

**Fig. 5:**
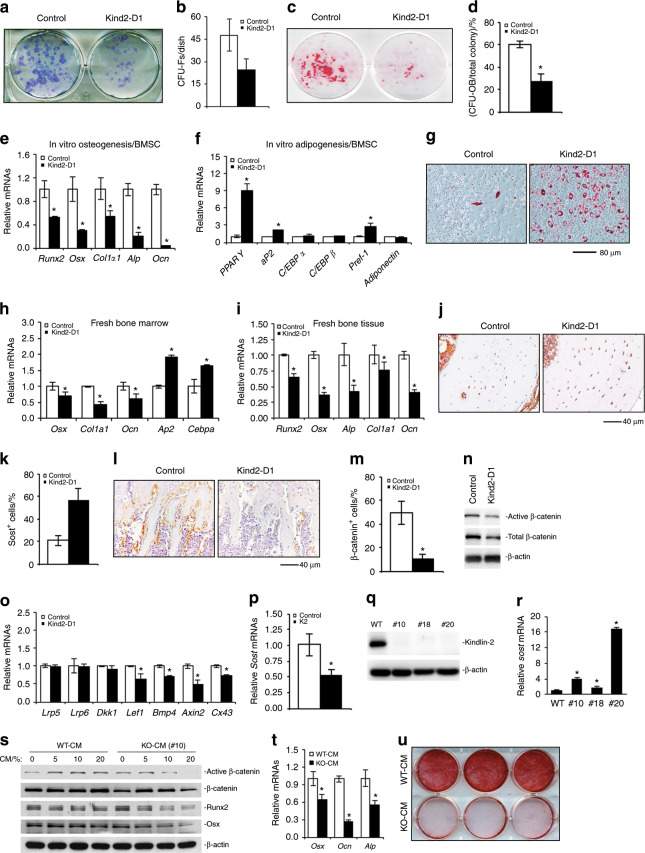
Kindlin-2 loss impairs osteoblast formation and function. **a**, **b** Colony forming unit-fibroblast (CFU-F) assays, followed by Giemsa staining (**a**). The number of CFU-F was counted under a microscope (**b**). *P < 0.05, versus controls, Student’s *t*-test. The results are expressed as the mean ± standard deviation (s.d.). *N* = 3 mice per group. (**c**, **d**) Colony forming unit-osteoblast (CFU-OB) assays. *P < 0.05, versus controls, Student’s *t*-test. The results are expressed as the mean ± standard deviation (s.d.). **e**–**g** In vitro osteoblastic or adipogenic differentiation of primary bone marrow stromal cells (BMSCs), followed by Oil Red O staining (**g**). *P < 0.05, versus controls, Student’s *t*-test. The results are expressed as the mean ± s.d. Scale bar, 80 mm. **h** Real-time RT-PCR (qPCR) analyses. *P < 0.05, versus controls, Student’s *t*-test. *N* = 4 mice per genotype. The results are expressed as the mean ± s.d. **i** Real-time RT-PCR (qPCR) analyses. *P < 0.05, versus controls, Student’s *t*-test. *N* = 4 mice per genotype. The results are expressed as the mean ± s.d. **j**, **k** Immunohistochemical (IHC) staining of tibial diaphyseal cross-sections of 2-month-old male *Kind2-D1* and their control littermates with an antibody against sclerostin. Scale bar, 40 mm. *N* = 8 control mice; *N* = 7 *Kind2-D1* mice. *P < 0.05, versus controls, Student’s *t*-test. The results are expressed as the mean ± s.d. **l**, **m** Immunohistochemical (IHC) staining of tibial metaphyseal longitudinal sections of 2-month-old male *Kind2-D1* and control littermates with an antibody against active b-catenin. Scale bar, 40 mm. *N* = 8 control mice; *N* = 7 *Kind2-D1* mice. *P < 0.05, versus controls, Student’s *t*-test. The results are expressed as the mean ± s.d. **n** Western blot analysis. **o** Real-time RT-PCR (qPCR) analyses. *N* = 3 control mice; *N* = 4 *Kind2-D1* mice. *P < 0.05, versus controls, Student’s *t*-test. The results are expressed as the mean ± s.d. **p** qPCR analysis. *P < 0.05, versus controls, Student’s *t*-test. The results are expressed as the mean ± s.d. **q** Crispr/Cas-9 deletion of Kindlin-2 in MLO-Y4 osteocyte-like cells. **r** qPCR analysis. *P < 0.05, versus controls, Student’s *t*-test. The results are expressed as the mean ± s.d. (**s**–**u**) MC-4 preosteoblastic cells were cultured in osteoblast differentiation media in the presence of the conditioned media from parent MLO-Y4 cells (WT-CM) or Kindlin-2-deficient subclone #10 (KO-CM) for 5 d, followed by western blotting with the indicated antibodies (**s**) or qPCR analysis of the indicated genes normalized to Gapdh mRNA (**t**), or 10 d, followed by Alizarin red staining (**u**).

The above results from single-cell sequencing analysis strongly suggest that Kindlin-2 deletion in Dmp1-expressing cells impairs the bone microenvironment. To determine if this is the case, we performed several sets of experiments. First, we performed a colony forming unit-fibroblast (CFU-F) assay and found that the number of CFU-F colonies was reduced by 48% in *Kind2-D1* group relative to the control group (Fig. 5a, b). The cell growth rate in CFU-F culture was decreased in the Kind2-D1 group compared with the control group (Supplementary Fig. 6). Second, we performed a colony forming unit-osteoblast (CFU-OB) assay and observed a markedly reduced number of CFU-OB (osteoprogenitors) in the *Kind2-D1* group compared with the control group (Fig. 5c, d). Third, we determined whether Kindlin-2 ablation impacted the in vitro differentiation capacity of primary bone marrow stromal cells (BMSCs) and found that the expression of Runx2, Osx, Col1a1, Alp, and Ocn was all dramatically decreased in the *Kind2-D1* group compared with the control group (Fig. 5e). Fourth, Kindlin-2 loss significantly increased the expression of adipogenic differentiation genes, including those encoding peroxisome proliferator-activated receptor gamma, adipocyte protein 2, and preadipocyte factor-1, but not C/EBPα, C/EBPβ, and adiponectin (Fig. 5f). Kindlin-2 loss promoted adipocyte formation in BMSC cultures, as measured by Oil Red O staining (Fig. 5g). Finally, the results from qPCR analyses using RNA isolated from fresh bone marrow or bone tissues of the two genotypes revealed that the expression of osteoblast genes was significantly reduced and the expression of adipocyte genes was increased in the bone marrow of *Kind2-D1* relative to their control littermates (Fig. 5h, i).

### Kindlin-2 deletion upregulates sclerostin expression in osteocytes and downregulates β-catenin expression in osteoblastic cells in bone and in vitro

Given the critical role of sclerostin in controlling bone mass, we determined whether Kindlin-2 loss affects sclerostin expression in osteocytes. We found that the number of osteocytes that express abundant sclerostin protein was dramatically increased in the cortical bone of *Kind2-D1* mice relative to their control littermates (Fig. 5j, k). Consistent with increased sclerostin expression, active β-catenin protein was drastically downregulated in osteoblasts and osteoprogenitors on the bone surfaces of *Kind2-D1* tibiae compared with control tibiae (Fig. 5l, m). Furthermore, the levels of both total and active β-catenin proteins were markedly reduced in the osteoblast-differentiated cultures of primary BMSCs from *Kind2-D1* mice compared with control cell cultures (Fig. 5n). Consistent with the reduced β-catenin level, the expression of its downstream genes, including those encoding Lef1, Bmp4, Axin2, and Cx43, was significantly downregulated in *Kind2-D1* bones relative bones of controls (Fig. 5o and Supplementary Fig. 7). In contrast, the expression levels of Lrp5 and Lrp6, both Wnt receptors, were not reduced in *Kind2-D1* bones compared with control bones (Fig. 5o). The results of in vitro studies revealed that overexpression of Kindlin-2 reduced the level of *sclerostin* mRNA in MLO-Y4 osteocyte-like cells (Fig. 5p), while deleting Kindlin-2 expression in MLO-Y4 cells using Crispr/Cas-9 technology increased the level of *sclerostin* mRNA (Fig. 5q, r). Treatment of murine MC-4 preosteoblastic cells with KO-CM (from subclone #10) reduced osteoblast gene expression and mineralization (Fig. 5s–u).

### Upregulation of β-catenin reverses the osteopenia induced by Kindlin-2 loss

**Fig. 6:**
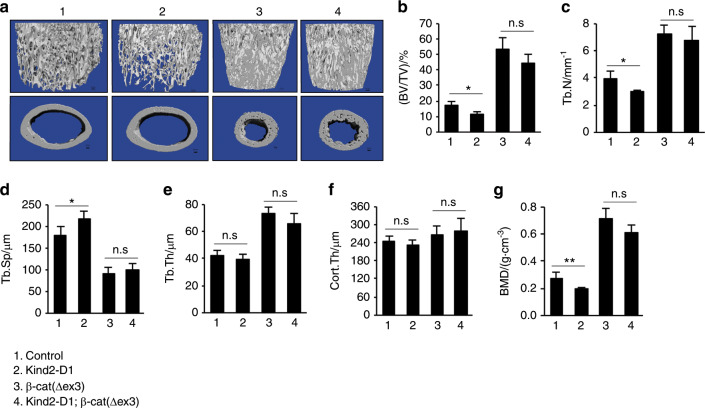
Upregulation of b-catenin reverses osteopenia in *Kind2-D1* mice. **a** Three-dimensional (3D) reconstruction from microcomputerized tomography (mCT) scans of distal femurs from 43-d-old male *Kind2-D1* mice and their control littermates. **b**–**g** Quantitative analyses of bone volume/tissue volume (BV/TV), trabecular number (Tb.N), trabecular thickness (Tb.Th), trabecular separation (Tb.Sp), cortical thickness (Cort.Th), and bone mineral density (BMD) of distal femurs from *Kind2-D1* mice and control littermates. *N* = 6 mice in the *Kind2-D1* group and *N* = 5 mice in the control, b-cat(∆ex3), and *Kind2-D1*; b-cat (∆ex) groups, Student’s *t*-test. The results are expressed as the mean ± standard deviation (s.d.). *P < 0.05, **P < 0.01, versus controls, Student’s *t*-test. The results are expressed as the mean ± s.d.

We next determined the effect of upregulation of β-catenin in the mutant cells on bone mass in *Kind2-D1* mice compared with control littermates. To this end, we bred *Kind2-D1* mice with *β-cat(∆ex3)*^*fl/fl*^ mice and generated *Kind2-D1; β-cat(∆ex3)*^*fl/+*^ mice. In these mice, expression of a constitutively active form of β-catenin that lacks the GSK phosphorylation sites (*β-cat(∆ex3)*) was driven by the 10-kb *Dmp1-Cre* transgene in *Kind2-D1* mice. Shockingly, almost all mice expressing the β-cat(∆ex3) protein (*Kind2-D1; β-cat(∆ex3)*^*fl/+*^ and *Dmp1-Cre*; *β-cat(∆ex3)*^*fl/+*^) died before the age of 50 days for unknown reason(s). This prevented us from analyzing the effect of β-catenin upregulation on bone in adult animals. We therefore performed μCT analyses of the femurs of young mice (43 days old) of the indicated groups (Fig. 6a). As expected, upregulation of β-catenin dramatically increased the bone mass (comparing group 1 to group 3). Furthermore, 43-day-old *Kind2-D1* mice displayed significant reductions in cancellous bone mass and BMD when compared with age- and sex-matched controls (Fig. 6b, c, g). Importantly, upregulation of β-catenin reversed the osteopenia of *Kind2-D1* mice (comparing group 3 to group 4) (Fig. 6b, c, g).

### Kindlin-2 loss increases RANKL expression in osteocytes and promotes osteoclast formation

**Fig. 7:**
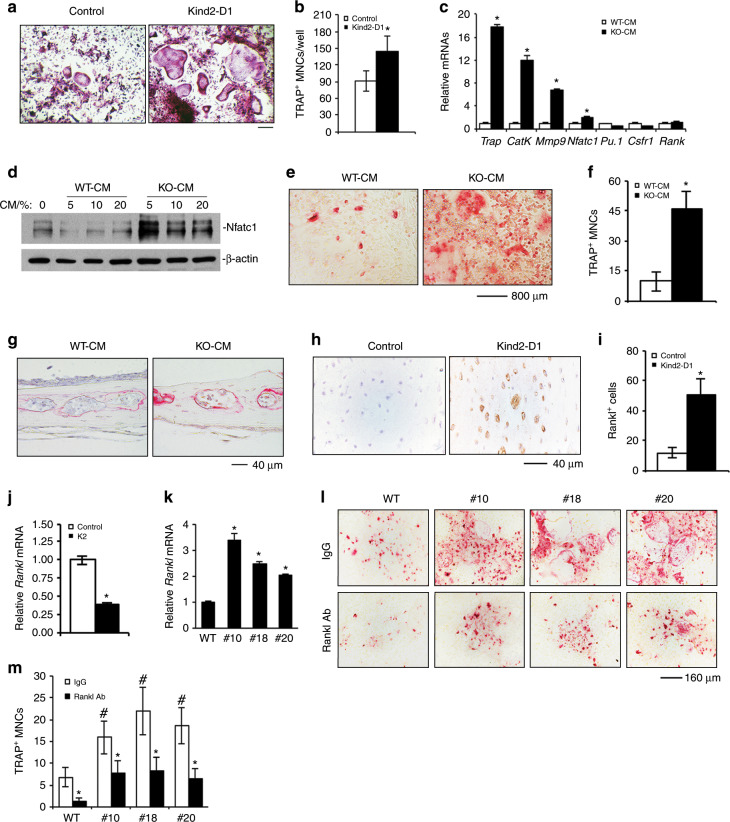
Kindlin-2 loss promotes RANKL expression in osteocytes and promotes osteoclast formation in vitro and in bone. **a**, **b** Tartrate-resistant acid phosphatase (TRAP) staining. Nonadherent bone marrow monocytes (BMMs) from 3-month-old female *Kind2-D1* mice and their control littermates were induced with the macrophage colony-stimulating factor (M-CSF) (10 ng·mL^−1^) and RANKL (50 ng·mL^−1^) for 7 d, followed by TRAP staining (**a**). TRAP-positive multiple nucleated cells (MNCs) with ≥3 nuclei per well were scored (**b**). *P < 0.05, versus controls, Student’s *t*-test. The results are expressed as the mean ± s.d. Scale bar, 160 mm. **c**–**f** Osteoclast gene expression and TRAP staining. Primary BMMs from 2-month-old wild-type male C57BL/6 mice were treated with WT-CM and KO-CM (from subclone #10) in the absence of exogenous RANKL for 7 d, followed by qPCR analysis, which were normalized to Gapdh mRNA (**c**); 2 d followed by western blotting for Nfatc1 (**d**); or 7 d followed by TRAP staining (**e**). TRAP-positive MNCs (≥3 nuclei) per well were scored (**f**). **g** Calvariae were dissected from 4-week-old wild-type C57BL/6 mice and cultured with 15% FBS a-MEM media in the presence of 20% WT-CM and KO-CM (#10) in the absence of exogenous RANKL for 3 d. *N* = 4 mice per group. Scale bar, 40 mm. **h**, **i** Immunohistochemical (IHC) staining of diaphyseal cross-sections of tibiae of 6-month-old male *Kind2-D1* mice and their control littermates with an antibody against RANKL. Scale bar, 40 mm. *N* = 6 control mice; *N* = 8 *Kind2-D1* mice. *P < 0.05, versus controls, Student’s *t*-test. The results are expressed as the mean ± s.d. **j** Real-time RT-PCR (qPCR) analysis. *P < 0.05, versus controls, Student’s *t*-test. The results are expressed as the mean ± s.d. (k) Real-time RT-PCR (qPCR) analysis.*P < 0.05, versus controls, Student’s *t*-test. The results are expressed as the mean ± s.d. (**l**, **m**) MLO-Y4/BMM coculture. Parent MLO-Y4 cells or Kindlin-2-deficient subclones (#10, #18, and #20) were cocultured with primary BMMs from 2-month-old wild-type C57BL/6 mice in the presence of 10 nmol·L^−1^ 1,25-dihydroxyvitamin D3 and in the presence of 40 ng normal goat IgG (top) or 40 ng goat anti-mouse RANKL-neutralizing antibody (bottom) for 8 d, followed by TRAP staining (**l**). Note: no RANKL was added to the cultures. TRAP-positive multiple nucleated cells (MNC) with ≥3 nuclei per well were scored (**m**). ^#^*P* < 0.05, versus WT, *P < 0.05, versus IgG, Student’s *t*-test. The results are expressed as the mean ± s.d. Scale bar, 160 mm. Experiments were performed with six samples per group and independently repeated two times; qualitatively identical results were obtained.

Given the dramatically increased osteoclast formation in *Kind2-D1* bones (Fig. 2o–s), we determined the effect of Kindlin-2 ablation on osteoclast differentiation in primary bone marrow monocyte (BMM) cultures. We assessed whether osteoclast formation and gene expression induced by exogenous RANKL were normal in *Kind2-D1* BMM cultures in comparison with control cultures. The results showed that osteoclast formation in *Kind2-D1* BMM cultures was significantly increased compared with control cultures (Fig. 7a, b). Furthermore, osteoclasts that formed in *Kind2-D1* BMM cultures were much larger than those formed in control cultures (Fig. 7a). These results suggest that BMMs are influenced before in vitro differentiation by the altered bone microenvironment in *Kind2-D1* mice. We next treated primary BMMs with WT-CM or KO-CM and found that KO-CM increased osteoclast gene expression and osteoclast formation independent of the addition of exogenous RANKL protein (Fig. 7c–f). Furthermore, KO-CM promoted osteoclast formation in ex vivo cultured calvariae without the addition of exogenous RANKL (Fig. 7g). Immunohistochemistry (IHC) staining of tibial cross sections showed increased expression of RANKL in the osteocytes of *Kind2-D1* mice (Fig. 7h, i). In vitro studies showed that overexpression of Kindlin-2 reduced while deletion of Kindlin-2 increased the mRNA level of *RANKL* in MLO-Y4 cells (Fig. 7j, k). The results from the MLO-Y4/BMM coculture experiments revealed that Kindlin-2 deletion largely increased osteoclast formation; this increase was partially blocked by RANKL-neutralizing antibody (Fig. 7l, m).

### Kindlin-2 loss decreases osteocyte spreading and dendrite formation and increases cell apoptosis

**Fig. 8:**
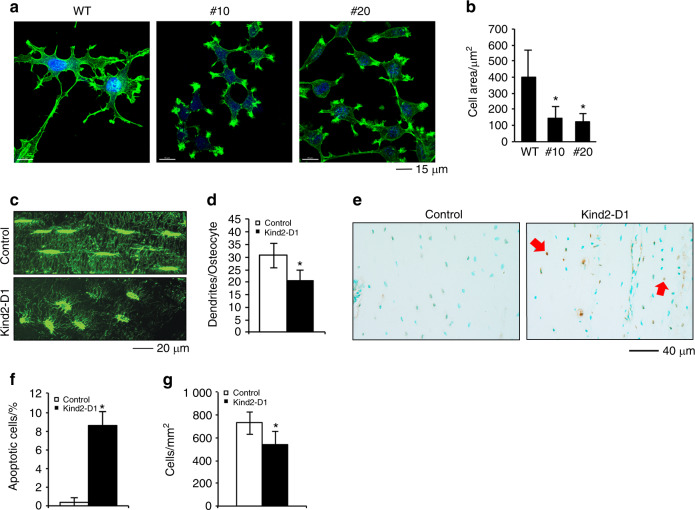
Kindlin-2 loss impairs osteocyte spreading and dendrite formation and increases cell apoptosis. **a**, **b** Confocal image analysis of MLO-Y4 osteocyte-like cells. Parent MLO-Y4 and Kindlin-2-deficient subclones (#10 and #20) were cultured in 8-well chambers (Thermo Fisher Scientific, Rochester, NY, USA, cat#: 154534) in a-MEM media for 24 h, followed by staining with 0.5% (v/v) Phalloidin-488 (Invitrogen, cat#, A12379). Scale bar, 15 mm. Measurements of cell area (b). *P < 0.05, versus wild-type (WT), Student’s *t*-test. The results are expressed as the mean ± standard deviation (s.d.). **c**, **d** Immunofluorescence (IF) staining. Undecalcified tibial sections of the two genotypes were stained with an anti-F-actin antibody. Scale bar, 20 mm. The numbers of osteocyte dendrites were quantified (**d**). *N* = 13 osteocytes from 6 control mice, *N* = 17 osteocytes from 6 *Kind2-D1* mice. *P< 0.05, versus control, Student’s *t*-test. The results are expressed as the mean ± standard deviation (s.d.). **e**, **f** Terminal deoxynucleotidyl transferase dUTP nick end labeling (TUNEL) staining. Tibial sections of 3-month-old female *Kind2-D1* mice and their control littermates were subjected to TUNEL staining (**e**). Quantification of apoptotic cells in the cortical bone matrix (**f**). Red arrows indicate apoptotic osteocytes imbedded in the bone matrix. *N* = 6 mice per group. *P < 0.05, versus controls, Student’s *t*-test. The results are expressed as the mean ± s.d. **g** Osteocyte density in cortical bones. *P < 0.05, versus WT, Student’s *t*-test. The results are expressed as the mean ± s.d. *N* = 9 mice for control, *N* = 10 mice for *Kind2-D1*.

We finally focused our studies on osteocytes. Deleting Kindlin-2 expression in MLO-Y4 osteocyte-like cells severely impaired cell spreading (Fig. 8a, b). The confocal analysis of osteocytes of cortical bone sections showed that Kindlin-2 loss significantly reduced the formation of osteocyte dendrites (Fig. 8c, d). It is interesting to note that deleting Kindlin-2 markedly altered the morphology of osteocytes in the bone matrix. Control osteocytes displayed an oval morphology, and mutant cells appeared to be round.

We performed terminal deoxynucleotidyl transferase dUTP nick end labeling (TUNEL) staining of tibial cross sections to investigate whether the loss of Kindlin-2 affects osteocyte apoptosis. As expected, fewer apoptotic osteocytes were observed in the matrix of control littermate bones; in contrast, the number of apoptotic osteocytes was significantly increased in *Kind2-D1* bones compared with control bones (Fig. 8e, f). Consistent with increased osteocyte apoptosis, the density of osteocytes in tibial diaphyseal cortical bones was reduced in *Kind2-D1* bones compared with control bones (Fig. 8g).

## Discussion

In the present study, through comprehensive analyses of cells and genetic mouse models, we establish a critical role of Kindlin-2 expression in osteocytes and mature osteoblasts in the control of bone mass and homeostasis in mice. Given the strikingly low bone mass phenotype due to abnormal bone remodeling in the altered bone microenvironment of *Kind2-D1* mice, our findings represent an important advance in bone biology.

One novel finding of this study is our demonstration that Kindlin-2 signaling in osteocytes and mature osteoblasts is a major determinant of the bone microenvironment. The altered bone microenvironment induced by Kindlin-2 loss reduces CFU-F and CFU-OB and increases osteoclast precursors. BMSCs from *Kind2-D1* mice tend to differentiate into adipocytes rather than osteoblasts. Interestingly, our recent studies demonstrated that deleting Kindlin-2 in MSCs suppresses osteogenic differentiation but accelerates adipogenic differentiation of MSCs. Kindlin-2 regulates MSC differentiation by control of YAP/TAZ.^30^

Drastically impaired osteoblast formation and function should largely contribute to the osteopenic phenotype in *Kind2-D1* mice. Reductions in CFU-F and CFU-OB and the impaired osteoblastic differentiation capacity of MSCs of *Kind2-D1* mice together decrease osteoblast number and bone formation, leading to severe osteopenia. Consistently, Osx^+^ cells (i.e., osteoblasts and osteoprogenitors) on bone surfaces are barely detected in *Kind2-D1* bones. The marked reductions in serum P1NP, osteoid production, MAR, and BFR in *Kind2-D1* mice further corroborate a severe impairment of bone formation. Impaired cell proliferation contributes to the reduced formation of CFU-F in *Kind2-D1* mice.

It is highly possible that Kindlin-2 signaling primarily from osteocytes regulates osteoblast formation and function by controlling sclerostin expression, which potently inhibits Wnts/β-catenin signaling. This notion is supported by multiple lines of evidence. First, sclerostin is almost exclusively produced by osteocytes,^12^ which account for 90%–95% of total bone cells. Second, Kindlin-2 loss greatly upregulates sclerostin in osteocytes in bone and in vitro. Third, the expression levels of β-catenin and its downstream target genes are dramatically reduced in osteoblasts in *Kind2-D1* bones. Fourth, conditioned media from Kindlin-2-deficient osteocytes dramatically reduces the levels of β-catenin, Runx2, and Osx proteins and suppresses osteoblast differentiation. Finally, upregulation of β-catenin in mutant cells reverses the osteopenic phenotype in *Kind2-D1* mice. Interestingly, Yu and coworkers showed that Kindlin-2 binds to β-catenin to promote tumor cell invasion and progression.^36,37^ Likewise, Lin et al. showed that Kindlin-2 accelerates hepatocellular carcinoma invasion and metastasis by upregulating Wnt/β-catenin signaling.^38^ Thus, Kindlin-2 can upregulate β-catenin through interactions with the latter and through an indirect mechanism involving sclerostin downregulation.

Increased sclerostin induced by osteocyte Kindlin-2 deficiency in the bone marrow microenvironment may play a crucial role in regulating the differentiation of bone marrow cells. Sclerostin is a potent inhibitor of Wnt/β-catenin signaling, which is known to promote osteoblastic differentiation but inhibits adipogenic differentiation of BMSCs. We cannot exclude other mechanism(s) through which Kindlin-2 loss in osteocytes impacts bone marrow cells.

We provide strong evidence that Kindlin-2 inhibits osteoclast formation and differentiation by reducing RANKL production in osteocytes, a main source of this key osteoclastogenic factor.^9,10^ *Kind2-D1* mice display increased osteoclast formation in bones. The increased osteoclast formation of primary *Kind2-D1* BMM cultures suggests that BMMs are influenced before in vitro differentiation by signals generated by Kindlin-2 loss in osteocytes. RANKL is greatly upregulated in osteocytes embedded in *Kind2-D1* bones and in Kindlin-2-deficient MLO-Y4 cells. Furthermore, conditioned media from Kindlin-2-deficient cells promotes osteoclast formation in primary BMM cultures and ex vivo cultured calvariae. The increase in osteoclast formation induced by Kindlin-2-deficient osteocytes in osteocyte/BMM cocultures is blocked by a RANKL-neutralizing antibody. Mechanism(s) through which Kindlin-2 loss increases RANKL production remain to be defined. Cumulative evidence suggests that increased osteocyte apoptosis may play a role in promoting local bone resorption and that apoptotic osteocytes may generate a pro-osteoclastogenic signal. Plotkin et al. reported that inhibition of osteocyte apoptosis suppressed osteocytic RANKL expression.^39^ Likewise, osteocyte apoptosis caused by hindlimb unloading triggers osteocyte RANKL production and subsequent bone resorption in mice.^40^ Thus, increased osteocyte apoptosis could partially contribute to the increased RANKL production in mutant osteocytes.

Kindlin-2 loss reduces osteocyte dendrite formation and impairs the communication of osteocytes with one another and with osteoclasts and osteoblasts located on bone surfaces, further damaging the bone microenvironment and bone homeostasis.

It should be noted that Kind2-D1 mice display severe osteopenia, while Kind2-Col1a1 mice only exhibit subtle osteopenia. We found that the percentage of Kindlin-2-expressing cells was reduced by 36% in Kind2-D1 compared with control littermates (from 89% to 56.9%), while the percentage of Kindlin-2-expressing cells was reduced by only 17.9% in Kind2-Col1a1 mice compared with control mice (from 87.6% to 71.9%) (Supplementary Fig. 8). Thus, it is possible that 10-kb Dmp1-Cre has a higher deleting efficiency for Kindlin-2 in osteocytes than 2.3-kb Col1a1-Cre. It is also possible that cells of the 2.3-kb mouse Col1a1-Cre lineage contribute less to osteocytes than those of the 10-kb Dmp1-Cre lineage. More detailed studies are required to explore these possibilities.

It has been reported that 10-kb Dmp1-Cre targets tissues other than bone, especially muscle.^41^ We did not observe significant differences in muscle weights between Kind2-D1 and control mice (Supplementary Fig. 9a, b). Furthermore, we did not observe apparent morphological abnormalities in Kind2-D1 muscles (Supplementary Fig. 9c). Most importantly, the level of Kindlin-2 protein was not markedly reduced in Kind2-D1 muscles compared with control muscles (Supplementary Fig. 9d). Collectively, these results do not support that the severe osteopenia in Kind2-D1 mice is caused by Kindlin-2 deletion in skeletal muscle.

Finally, mechanical loading is the most potent anabolic stimulus of bone, and the skeleton is constantly loaded by various mechanical forces generated from daily physical activities and gravity. The severe osteopenia in Kind2-D1 mice highlights the need to investigate whether osteocyte Kindlin-2 signaling mediates mechanotransduction in bone.

## Materials and Methods

### Animal studies

The generation of *Kindlin-2*^*fl/fl*^ mice was recently described.^29^ *Dmp1-Cre* transgenic mice, in which a 10-kb mouse *Dmp1* gene promoter drives Cre recombinase expression in osteocytes, were also previously described.^31^ *Col1a1-Cre* transgenic mice, in which a 2.3-kb mouse *Col1a1* gene promoter drives Cre expression in osteoblastic cells, were purchased from MGI.^42^ *β-catenin (ex3)*^*fl/fl*^ mice that harbor a mutant β-catenin allele whose exon 3 is floxed by loxP sequences were previously described.^43^ The animal protocols of this study were approved by the Institutional Animal Care and Use Committee of Southern University of Science and Technology.

### Histological evaluation and bone histomorphometry

For histological analyses, bone and other tissues were prepared, fixed, decalcified, and embedded using our standard protocols. Fixed nondemineralized femurs were subjected to microcomputerized tomography (μCT) analysis in the Department of Biology of Southern University of Science and Technology using a Bruker μCT (SkyScan 1172 Micro-CT, Bruker MicroCT, Kontich, Belgium) as previously described.^44^

### Mineral apposition rate (MAR), mineralizing surface per bone surface (MS/BS), and bone formation rate (BFR)

MAR, MS/BS, and BFR were determined as we previously described.^45^

### Quantitative real-time RT-PCR (qPCR) and western blot analyses

RNA isolation, reverse transcription (RT), and qPCR analyses were performed as we previously described.^46^ The DNA sequences of the mouse primers used for qPCR are summarized in Supplementary Table 2. Western blot analyses were conducted as we previously described.^45^ The antibodies used in this study are listed in Supplementary Table 3.

### Immunohistochemistry (IHC) and immunofluorescence (IF)

Five micrometer sections were used for IHC staining with antibodies or control IgG using the EnVision^+^System-HRP (DAB) kit (Dako North America Inc, Carpinteria, CA, USA). For IF staining, cells were cultured on sterile glass cover slips in six-well plates. Twenty-four hours later, the cells were fixed, permeabilized, blocked with 2% bovine serum albumin, and stained with primary antibody using our standard protocol. After washing, cells were incubated with anti-mouse Alexa Fluor 594 (Invitrogen, Carlsbad, CA, USA) secondary antibodies (1:300) for 1 h at room temperature. Images were obtained using a confocal microscope (SP2-AOBS Leica Microsystems, Wetzlar, Germany).

### Single-cell library construction and sequencing

Primary bone marrow cells from three 3-month-old *Kind2-D1* male mice and three control littermates were treated with red blood cell lysis buffer and cultured in proliferating media to obtain enough cells. Then, equal numbers of adhesive cells from each culture of the same group were pooled and processed properly based on the 10× Genomics Chromium Single Cell 3′ protocol (v2 Chemistry). Quality control and quantification were performed using an Agilent 4200 TapeStation System (Agilent Technologies, California) and a QuantStudio 5 Real-Time PCR System (Thermo Fisher Scientific, Massachusetts). Sequencing (PE 150 bp) was completed using a NovaSeq 6000 Sequencing System (Illumina, California).

### Single-cell RNA sequencing data analysis

Single cell 3’ RNA-seq FASTQ files were subjected to alignment, filtering, barcode counting, and UMI counting using Cell Ranger (version 2.2.0) with default parameters. The mouse reference dataset mm10 required for Cell Ranger was downloaded from 10× Genomics support. In total, 4 669 cells with 31 264 mean reads, 2 028 median genes, and 17 186 genes were detected in the control group, while 3 549 cells with 28 116 mean reads, 1 795 median genes, and 16 415 genes were detected in *Kind2-D1* cells. The output from Cell Ranger was imported into Seurat (version 2.3.4), and downstream analysis was performed.^47^ Genes detected in fewer than five cells were removed, and cells with more than 0.1% mitochondrial genes or less than 750 detected genes were filtered out. Furthermore, for data from the control group, cells with detected genes below 1 250 and an average of 2.5 UMIs per gene were filtered out. As a result, 4 557 cells with a total of 14 332 genes from the control group and 3 489 cells with a total of 13 734 genes from the *Kind2-D1* group were merged together, and a new combined dataset with 8 046 cells and 14 564 genes was generated for subsequent analysis. The top 3 396 highly variable genes with an average expression higher than 0.01 and a dispersion value greater than 0.1 were used for principle component analysis, and a total of 30 PCs were computed. For easier data visualization, further dimensionality reduction by t-distributed stochastic neighbor embedding or uniform manifold approximation and projection (UMAP) based on the first ten PCs was performed.^48,49^ A total of 13 clusters of cells were finally defined with the first ten PCs and resolution as 0.9 by the FindCluster function in Seurat. UMAP was computed with uwot (https://github.com/jlmelville/uwot) (version 0.0.0.9010). Differentially expressed genes between the control and *Kind2-D1* groups with adjusted *P* values lower than 0.01 were collected for enrichment analysis using Metascape (http://metascape.org/gp/index.html). As default, gene sets from the KEGG pathway (https://www.genome.jp/kegg/), GO Biological Processes (http://www.geneontology.org/), Reactome Gene Sets (https://www.reactome.org/), and CORUM database (https://mips.helmholtz-muenchen.de/corum/) were used in Metascape.

### In vitro and in vivo osteoclast differentiation

In vitro osteoclast formation was conducted as we previously described.^50^ TRAP staining of bone sections was performed as we previously described.^51^ The osteoclast surface and osteoclast number were determined as we previously described.^52^

### TUNEL staining of bone sections

Osteocyte apoptosis in bone sections was measured using an ApopTag Peroxidase In Situ Apoptosis Detection Kit (EMD Millipore Corporation, Temecula, CA, USA, cat#: S7100) as we previously described.^53^

### CFU-F assay and CFU-OB assay

CFU-Fs and CFU-OBs were measured in primary bone marrow cultures as we previously described.^52^

### ELISA

Serum levels of P1NP were measured using a RatLaps EIA Kit (Immunodiagnostic Systems Limited, Gaithersburg, MD, USA, cat#: AC-33F1). Serum levels of CTX were measured using a RatLaps EIA Kit (Immunodiagnostic Systems Limited, Gaithersburg, MD, USA, cat#: AC-06F1).

### CRISPR/Cas-9 technology

CRISPR/Cas-9 deletion of Kindlin-2 expression in MLO-Y4 cells was performed using the method described by Ran et al.^54^ Mutation and sequencing primers for CRISPR/Cas9 deletion of mouse Kindlin-2 in this study are listed in Supplemental Tables 4 and 5.

### Statistical analyses

The sample size for each experiment was determined based on our previous experiences. No randomization or blinding was applied in the studies. Student’s *t* test was used to test for differences between two groups of data. The results are expressed as the mean ± standard deviation, as indicated in the figure legends. Differences with *P* < 0.05 were considered statistically significant.

## Supplementary information

Focal adhesion protein Kindlin-2 regulates bone homeostasis in mice

Figure S1

Figure S2

Figure S3

Figure S4

Figure S5

Figure S6

Figure S7

Figure S8

Figure S9

## Data Availability

All data generated for this study are available from the corresponding authors upon reasonable request.
