# Supplementary material for: Focal adhesion protein Kindlin-2 regulates bone homeostasis in mice
Source: Bone Res. 2020 Jan 2;8:2. doi: 10.1038/s41413-019-0073-8 (PMC6946678; doi:10.1038/s41413-019-0073-8)
Supplement: Supplementary file 1 — Focal adhesion protein Kindlin-2 regulates bone homeostasis in mice [file 41413_2019_73_MOESM1_ESM.pdf]

Figure S1

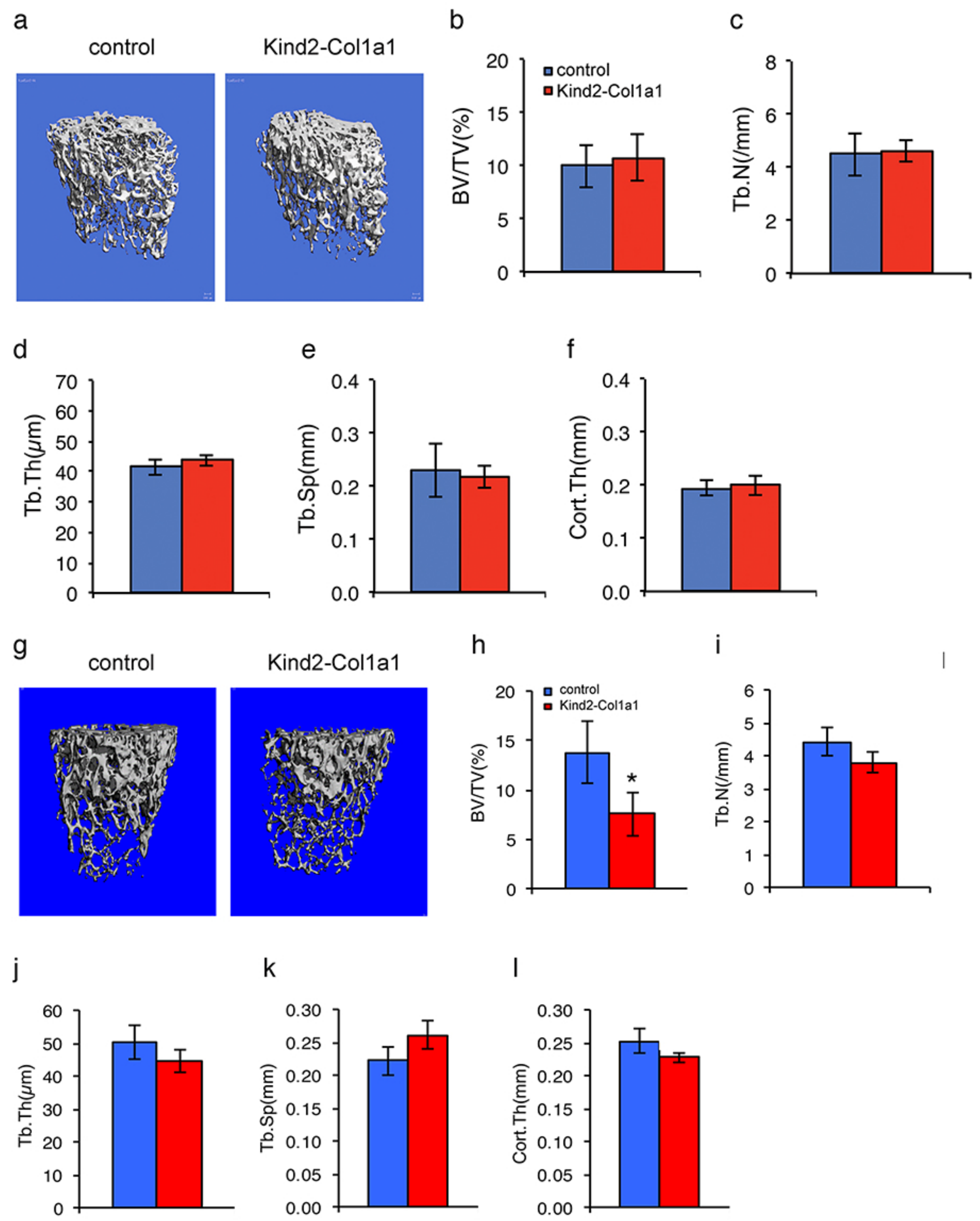

a
control
Kind2-Col1a1
b
BV/TV(%)
control
Kind2-Col1a1
c
Tb.N(/mm)
d
Tb.Th(μm)
e
Tb.Sp(mm)
f
Cort.Th(mm)
g
control
Kind2-Col1a1
h
BV/TV(%)
control
Kind2-Col1a1
*
i
Tb.N(/mm)
j
Tb.Th(μm)
k
Tb.Sp(mm)
l
Cort.Th(mm)

Figure S2

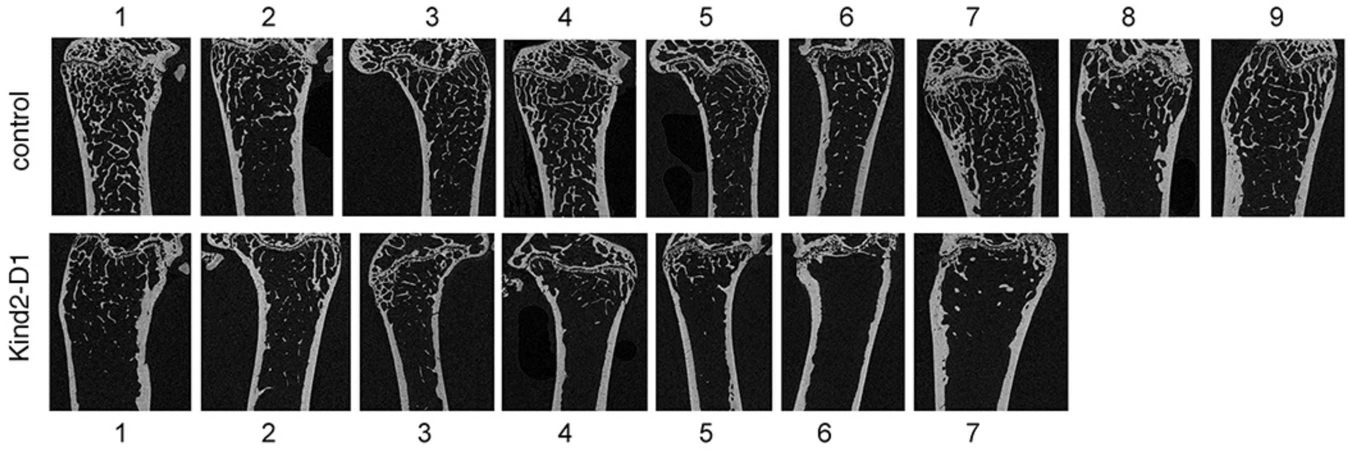

Figure S3

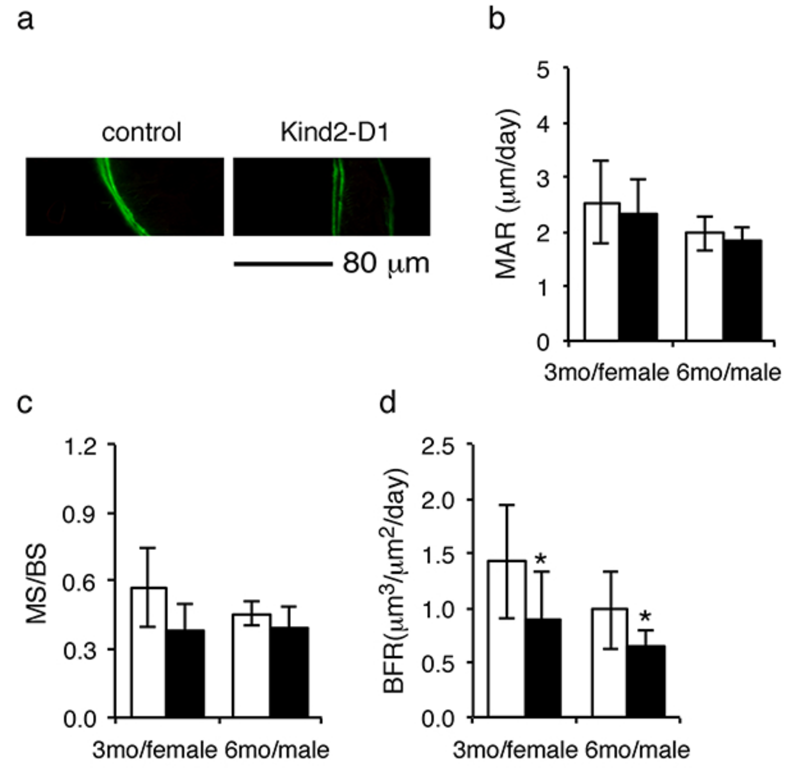

a
control
Kind2-D1
80 μm
b
MAR (μm/day)
5
4
3
2
1
0
3mo/female
6mo/male
c
MS/BS
1.2
0.9
0.6
0.3
0.0
3mo/female
6mo/male
d
BFR(μm3/μm2/day)
2.5
2.0
1.5
1.0
0.5
0.0
*
*
3mo/female
6mo/male

Figure S4

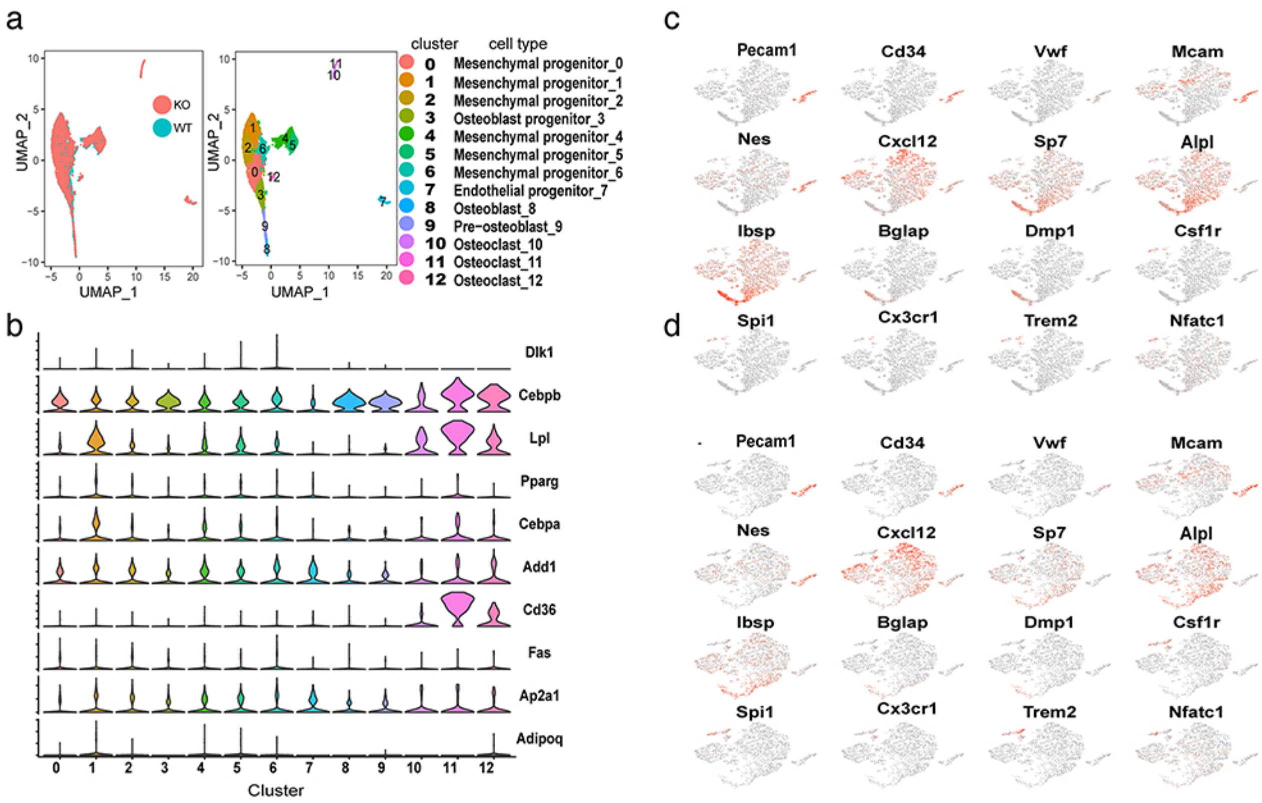
a
KO
WT
UMAP_1
UMAP_2
cluster
cell type
0 Mesenchymal progenitor_0
1 Mesenchymal progenitor_1
2 Mesenchymal progenitor_2
3 Osteoblast progenitor_3
4 Mesenchymal progenitor_4
5 Mesenchymal progenitor_5
6 Mesenchymal progenitor_6
7 Endothelial progenitor_7
8 Osteoblast_8
9 Pre-osteoblast_9
10 Osteoclast_10
11 Osteoclast_11
12 Osteoclast_12
b
Dlk1
Cebpb
Lpl
Pparg
Cebpa
Add1
Cd36
Fas
Ap2a1
Adipoq
Cluster
c
Pecam1
Cd34
Vwf
Mcam
Nes
Cxcl12
Sp7
Alpl
Ibsp
Bglap
Dmp1
Csf1r
d
Spi1
Cx3cr1
Trem2
Nfatc1
Pecam1
Cd34
Vwf
Mcam
Nes
Cxcl12
Sp7
Alpl
Ibsp
Bglap
Dmp1
Csf1r
Spi1
Cx3cr1
Trem2
Nfatc1

Figure S5

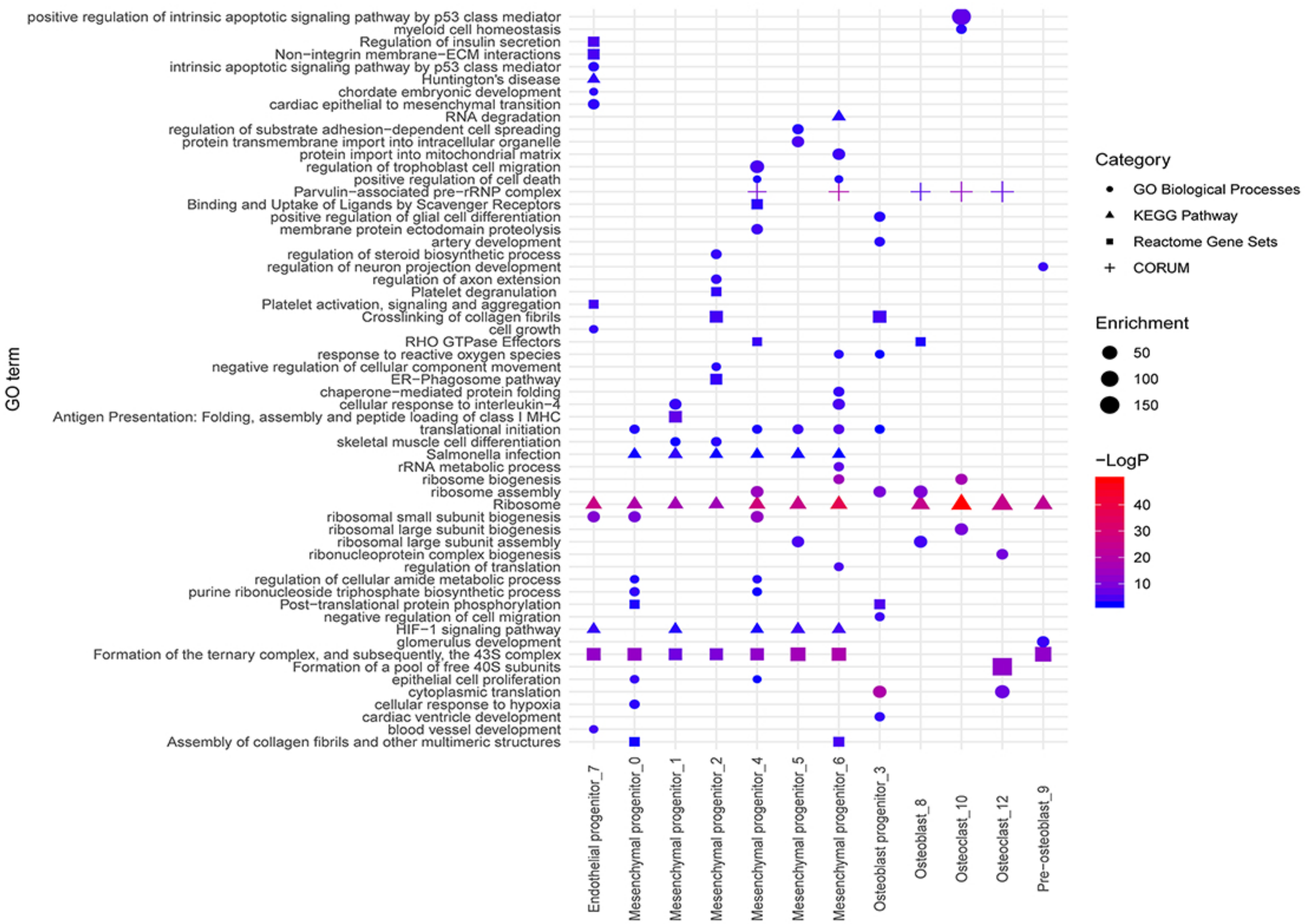

GO term
positive regulation of intrinsic apoptotic signaling pathway by p53 class mediator
myeloid cell homeostasis
Regulation of insulin secretion
Non−integrin membrane−ECM interactions
intrinsic apoptotic signaling pathway by p53 class mediator
Huntington's disease
chordate embryonic development
cardiac epithelial to mesenchymal transition
RNA degradation
regulation of substrate adhesion−dependent cell spreading
protein transmembrane import into intracellular organelle
protein import into mitochondrial matrix
regulation of trophoblast cell migration
positive regulation of cell death
Parvulin−associated pre−rRNP complex
Binding and Uptake of Ligands by Scavenger Receptors
positive regulation of glial cell differentiation
membrane protein ectodomain proteolysis
artery development
regulation of steroid biosynthetic process
regulation of neuron projection development
regulation of axon extension
Platelet degranulation
Platelet activation, signaling and aggregation
Crosslinking of collagen fibrils
cell growth
RHO GTPase Effectors
response to reactive oxygen species
negative regulation of cellular component movement
ER−Phagosome pathway
chaperone−mediated protein folding
cellular response to interleukin−4
Antigen Presentation: Folding, assembly and peptide loading of class I MHC
translational initiation
skeletal muscle cell differentiation
Salmonella infection
rRNA metabolic process
ribosome biogenesis
ribosome assembly
Ribosome
ribosomal small subunit biogenesis
ribosomal large subunit biogenesis
ribosomal large subunit assembly
ribonucleoprotein complex biogenesis
regulation of translation
regulation of cellular amide metabolic process
purine ribonucleoside triphosphate biosynthetic process
Post−translational protein phosphorylation
negative regulation of cell migration
HIF−1 signaling pathway
glomerulus development
Formation of the ternary complex, and subsequently, the 43S complex
Formation of a pool of free 40S subunits
epithelial cell proliferation
cytoplasmic translation
cellular response to hypoxia
cardiac ventricle development
blood vessel development
Assembly of collagen fibrils and other multimeric structures
Endothelial progenitor_7
Mesenchymal progenitor_0
Mesenchymal progenitor_1
Mesenchymal progenitor_2
Mesenchymal progenitor_4
Mesenchymal progenitor_5
Mesenchymal progenitor_6
Osteoblast progenitor_3
Osteoblast_8
Osteoclast_10
Osteoclast_12
Pre−osteoblast_9
Category
GO Biological Processes
KEGG Pathway
Reactome Gene Sets
CORUM
Enrichment
50
100
150
−LogP
40
30
20
10

Figure S6

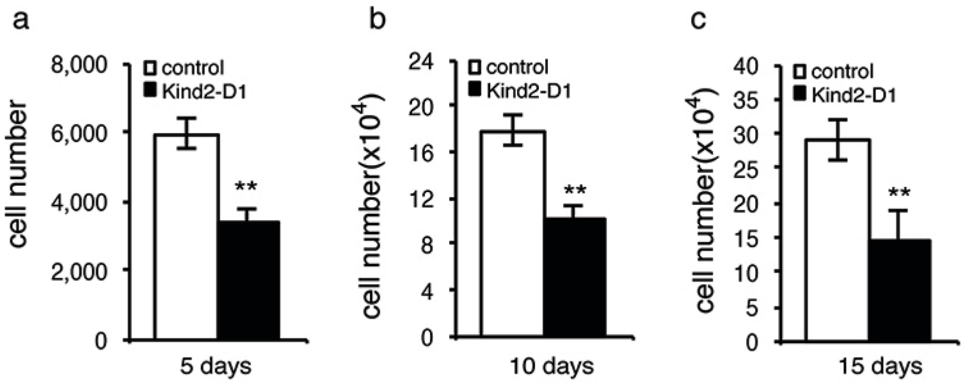

a
8,000
6,000
4,000
2,000
0
cell number
control
Kind2-D1
**
5 days
b
24
20
16
12
8
4
0
cell number(x10^4)
control
Kind2-D1
**
10 days
c
40
35
30
25
20
15
10
5
0
cell number(x10^4)
control
Kind2-D1
**
15 days

Figure S7

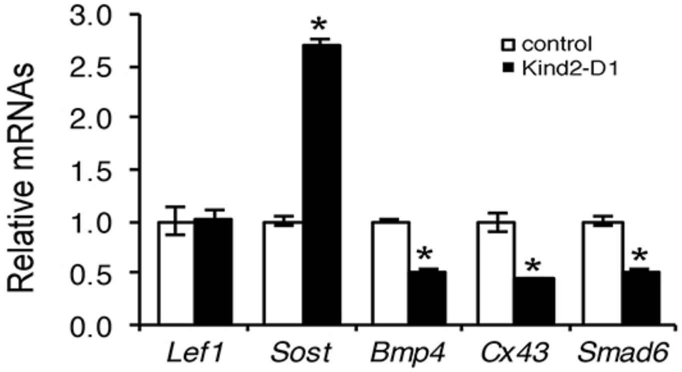

Relative mRNAs
3.0
2.5
2.0
1.5
1.0
0.5
0.0
control
Kind2-D1
*
*
*
*
Lef1
Sost
Bmp4
Cx43
Smad6

Figure S8

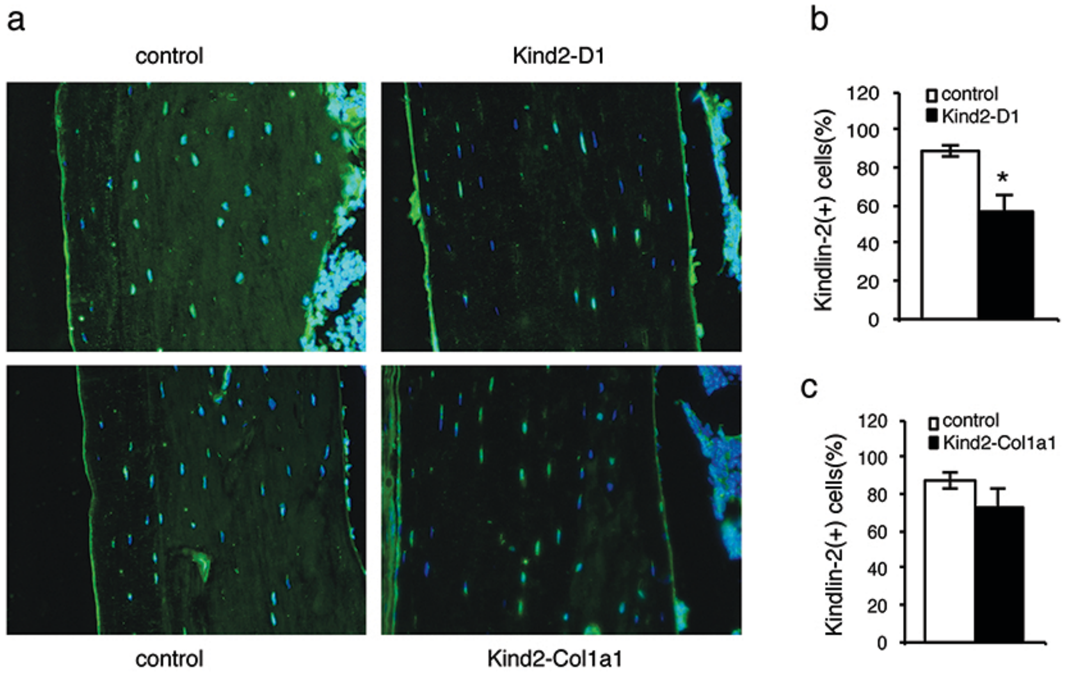
a
control
Kind2-D1
control
Kind2-Col1a1
b
Kindlin-2(+) cells(%)
control
Kind2-D1
*
120
100
80
60
40
20
0
c
Kindlin-2(+) cells(%)
control
Kind2-Col1a1
120
100
80
60
40
20
0

Figure S9

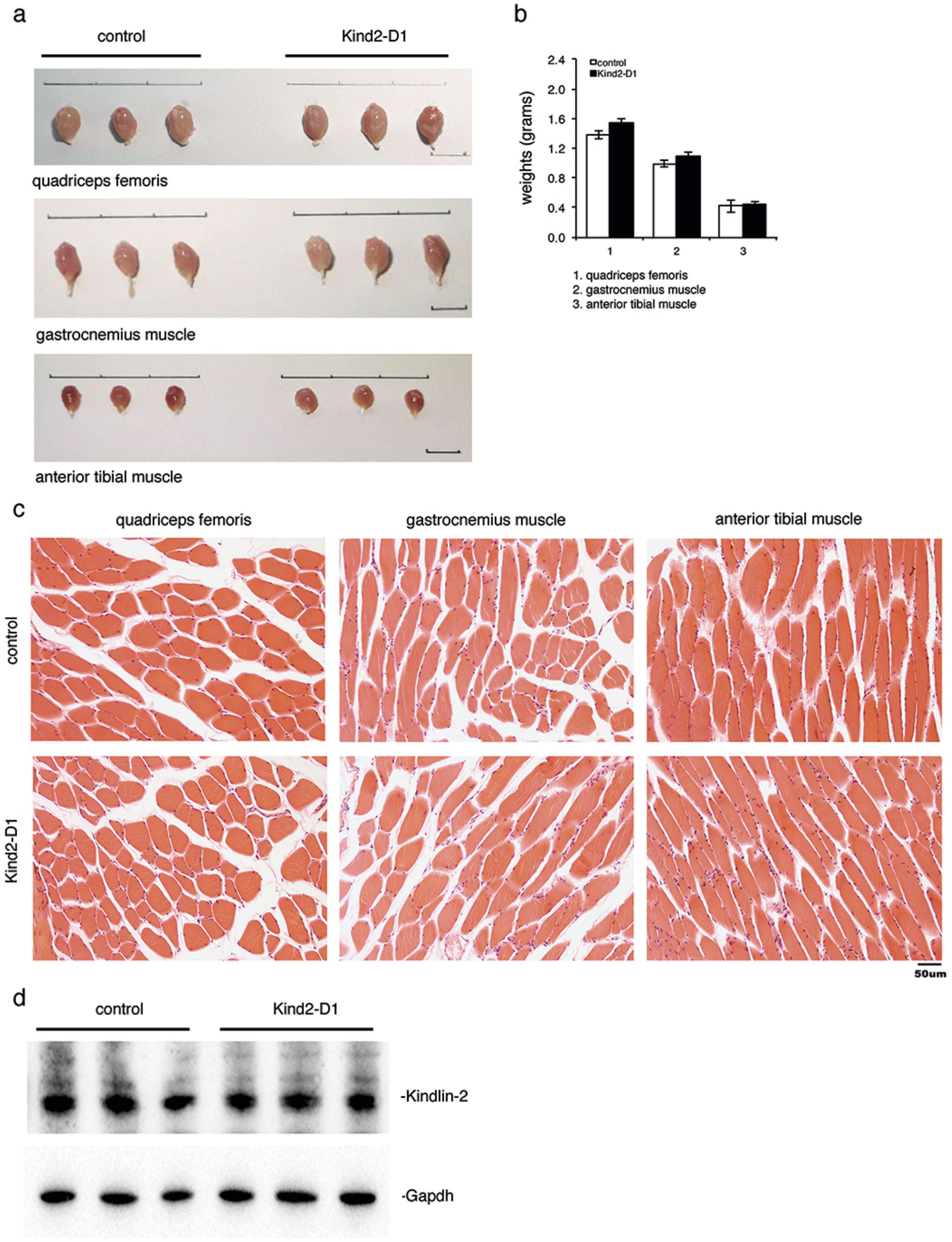
a
control
Kind2-D1
quadriceps femoris
gastrocnemius muscle
anterior tibial muscle
b
weights (grams)
2.4
2.0
1.6
1.2
0.8
0.4
0.0
control
Kind2-D1
1
2
3
1. quadriceps femoris
2. gastrocnemius muscle
3. anterior tibial muscle
c
quadriceps femoris
gastrocnemius muscle
anterior tibial muscle
control
Kind2-D1
50um
d
control
Kind2-D1
-Kindlin-2
-Gapdh

SUPPLEMENTAL INFORMATION

Supplementary Figure 1. Deleting Kindlin-2 expression in osteoblasts using the 2.3-kb mouse *Col1a1-Cre* transgenic mice slightly reduces bone mass in 4-, but not 2-, month-old mice. (**a**) Three-dimensional (3D) reconstruction from microcomputed tomography (μCT) scans of femurs from 2-month-old *Kind2-Col1a1* and control male mice. (**b-f**) Quantitative analysis of BV/TV, Tb.Th, Tb.N, Tb.Sp, and Cort.Th. *N* = 4 mice per group. (**g**) 3D reconstruction from μCT scans of femurs from 4-month-old *Kind2-Col1a1* and control male mice. (**h-j**) Quantitative analysis of BV/TV, Tb.Th, Tb.N, Tb.Sp, and Cort.Th. *N* = 4 mice per group.

Supplementary Figure 2. Kindlin-2 ablation dramatically reduces bone mass in 6-month-old male mice. Two-dimensional (2D) reconstruction from microcomputed tomography (μCT) scans of femurs from 6-mo-old male *Kind2-D1* mice and control littermates. N = 9 for control, N = 7 for *Kind2-D1* mice.

Supplementary Figure 3. Effects of Kindlin-2 loss on MAR, MS/BS and BFR in diaphyseal cortical bones. (**a**) Calcein double labeling. Sections of non-demineralized femurs of 6-mo-old control and *Kind2-D1* male mice were used for MAR, MS/BS, and BFR assays. (**b-d**) Quantitative MAR, MS/BS, and BFR data for diaphyseal cortical bones. Scale bar, 80 μm. *N* = 11 control, *N* = 10 for *Kind2-D1*.

Supplementary Figure 4. Four major cell types are identified with specific markers. (a) Data visualization using UMAP. (b) Vlnplot with markers of adipocytes. Important

adipocyte-related markers, including *Dlk1, Pparg*, and *Adipoq*, were not significantly expressed in any clusters. (c) TSNEPlot with indicated markers in control group cells. (d) TSNEPlot with indicated markers in *Kind2-D1* group cells.

Supplementary Figure 5. Gene set enrichment analysis results using metascape with *P* value lower than 1E-4. Genes with adjusted *P* value lower than 0.01 and absolute log2 fold change higher than 0.25 were used as input gene set for enrichment analysis by metascape with default parameters.

Supplementary Figure 6. Reduced growth rate of CFU-F cultures from Kind2-D1 mice. (**a-c**) Bone marrow nucleated cells (4×10^5 cells) from 2-mo-old male Kind2-D1 mice and control littermates were seeded in 24-well plates and cultured in media from the CFU-F assay kit. At d5 (a), d10 (b), and d15 (c), adhering cells were digested by trypsin and counted. N = 3 mice per group. **P*< 0.05, versus controls, Student's t-test. Results are expressed as mean ± standard deviation (s.d.).

Supplementary Figure 7. Effects of Kindlin-2 loss on expression of sclerostin and Wnt/β-catenin downstream target genes in bones. Real-time RT-PCR (qPCR) analyses. Tibiae were harvested from 2-mo-old male *Kind2-D1* mice and control littermates, growth plates at both ends were cut, and the bone marrow was flushed. Total RNA was isolated and used for qPCR analysis for expression of indicated genes and normalized to *Gapdh* mRNA. **P*<0.05, versus controls, Student's *t*-test. Results are expressed as mean ± standard deviation.

Supplementary Figure 8. Kindlin-2 expression in osteocytes in Kind2-D1 and Kind2-Col1a1 mice. (**a-c**) Immunofluorescence (IF) staining of tibial sections of 4-mo-old male Kind2-D1, Kind2-Col1a1 and their respective control littermates with an anti-Kindlin-2 antibody. Quantitative data of Kindlin-2-positive osteocytes embedded in the cortical bone matrix (b,c). N = 4 mice for Kind2-D1 and control, N = 5 mice for Kind2-Col1a1 and control. $^*P< 0.05$, versus controls, Student's t-test. Results are expressed as mean ± s.d.

Supplementary Figure 9. Skeletal muscles are not markedly affected in Kind2-D1 mice. (**a,b**) Muscle weight. The weights of the quadriceps femoris, gastrocnemius muscle, and anterior tibial muscle from 5-mo-old Kind2-D1 male mice and sex-matched control littermates were measured. N = 3 mice per group. Results are expressed as mean ± standard deviation (s.d.). (**c**) Hematoxylin and eosin (H/E) staining. Sections of above muscles were subjected to H/E staining. Scale bar: 50 μm. (**d**) Western blotting. Protein extracts from above quadriceps femoris were subjected to western blotting analysis using an anti-Kindlin-2 antibody. Gapdh protein was used as a loading control.

Supplementary Table 1

Differentially expressed genes between control group and Kind2-D1 group with adjusted *P* value lower than 0.01
P value calculated by FindMarkers Function with default wilcoxon sign test in Seurat (version 2.3.4)
pct.1, pct.2 represented percentage of cells with expression higher than 0
P value adjusted using bonferroni method

gene	p value	avg_logFC	pct.1	pct.2	adjusted p value	cluster	cell type
Rps28	8.67E-200	-0.935195574	1	0.998	1.26E-195	0	Mesenchymal progenitor_0
Rps29	1.02E-180	-0.647822936	1	1	1.49E-176	0	Mesenchymal progenitor_0
Rpl38	2.08E-160	-0.651818534	1	1	3.02E-156	0	Mesenchymal progenitor_0
Rpl41	5.62E-140	-0.453126906	1	1	8.19E-136	0	Mesenchymal progenitor_0
Gm8730	5.53E-136	0.74693076	1	0.988	8.06E-132	0	Mesenchymal progenitor_0
Rps2	3.10E-117	0.542896696	0.999	0.99	4.52E-113	0	Mesenchymal progenitor_0
Gm26917	9.81E-110	0.750494002	0.961	0.755	1.43E-105	0	Mesenchymal progenitor_0
Rps27	1.16E-105	-0.421216668	1	1	1.69E-101	0	Mesenchymal progenitor_0
Gm10076	3.56E-88	-0.381444646	1	0.998	5.19E-84	0	Mesenchymal progenitor_0
Rpl15	1.11E-86	0.542364399	0.994	0.945	1.62E-82	0	Mesenchymal progenitor_0
Rpl36	3.83E-86	-0.392695295	0.999	0.997	5.58E-82	0	Mesenchymal progenitor_0
Rpl37a	4.88E-78	-0.305913084	1	1	7.10E-74	0	Mesenchymal progenitor_0
mt-Nd2	1.95E-76	-0.717027106	0.772	0.926	2.84E-72	0	Mesenchymal progenitor_0
Eif2s2	7.41E-75	-0.581670875	0.931	0.983	1.08E-70	0	Mesenchymal progenitor_0
Atp5k	1.11E-74	-0.642080973	0.873	0.962	1.62E-70	0	Mesenchymal progenitor_0
Rplp0	5.17E-70	0.417087355	1	0.99	7.52E-66	0	Mesenchymal progenitor_0
Nmt1	4.06E-68	-0.727667996	0.29	0.675	5.92E-64	0	Mesenchymal progenitor_0
Nme2	8.06E-59	0.651753479	0.768	0.411	1.17E-54	0	Mesenchymal progenitor_0
Rpl37	8.66E-58	-0.276906921	0.999	1	1.26E-53	0	Mesenchymal progenitor_0

Snhg9	1.10E-56	-0.668090214	0.095	0.449	1.61E-52	0	Mesenchymal progenitor_0
Rps21	4.66E-52	-0.339592262	0.994	0.993	6.78E-48	0	Mesenchymal progenitor_0
Ftl1	4.43E-51	0.330716669	1	1	6.46E-47	0	Mesenchymal progenitor_0
Gm10260	1.52E-49	0.3206545	0.998	0.99	2.21E-45	0	Mesenchymal progenitor_0
Rps18	1.43E-47	0.291553747	1	0.991	2.09E-43	0	Mesenchymal progenitor_0
Sertad2	8.56E-47	-0.671570854	0.429	0.71	1.25E-42	0	Mesenchymal progenitor_0
Malat1	2.37E-46	-0.479263275	1	1	3.45E-42	0	Mesenchymal progenitor_0
Rpl29	6.11E-46	0.488437395	0.885	0.653	8.89E-42	0	Mesenchymal progenitor_0
Sec61g	7.14E-44	-0.305412901	0.996	0.991	1.04E-39	0	Mesenchymal progenitor_0
Lmo4	7.41E-42	-0.632562551	0.554	0.748	1.08E-37	0	Mesenchymal progenitor_0
Rpl39	7.73E-40	-0.348435048	0.998	0.998	1.13E-35	0	Mesenchymal progenitor_0
Ndufa3	1.71E-37	-0.461780816	0.851	0.919	2.49E-33	0	Mesenchymal progenitor_0
Gm10263	7.84E-36	-0.459843728	0.048	0.283	1.14E-31	0	Mesenchymal progenitor_0
Rps7	1.04E-35	0.275904451	1	0.986	1.52E-31	0	Mesenchymal progenitor_0
Rpl10	2.23E-33	0.262878249	0.999	0.99	3.25E-29	0	Mesenchymal progenitor_0
Gm10709	6.06E-32	0.31671197	0.975	0.922	8.83E-28	0	Mesenchymal progenitor_0
Calr	7.71E-31	0.392189753	0.949	0.862	1.12E-26	0	Mesenchymal progenitor_0
Rps27rt	2.81E-29	-0.485096825	0.381	0.615	4.10E-25	0	Mesenchymal progenitor_0
Timp1	3.41E-29	0.46889641	0.956	0.917	4.96E-25	0	Mesenchymal progenitor_0
Igkc	6.91E-29	0.408680515	0.401	0.128	1.01E-24	0	Mesenchymal progenitor_0
Usmg5	1.66E-28	-0.376840857	0.816	0.898	2.42E-24	0	Mesenchymal progenitor_0
Ibsp	2.13E-28	0.284494962	0.857	0.513	3.10E-24	0	Mesenchymal progenitor_0
Gm10020	3.68E-28	0.401382107	0.651	0.363	5.36E-24	0	Mesenchymal progenitor_0
Ybx1	7.08E-28	0.306285511	0.974	0.952	1.03E-23	0	Mesenchymal progenitor_0

Lox	6.06E-27	0.408871851	0.986	0.934	8.83E-23	0	Mesenchymal progenitor_0
Fos	5.14E-26	-0.614193958	0.575	0.739	7.48E-22	0	Mesenchymal progenitor_0
Hes1	1.93E-24	-0.59068097	0.457	0.641	2.81E-20	0	Mesenchymal progenitor_0
Ogn	2.26E-24	-0.448642582	0.727	0.848	3.30E-20	0	Mesenchymal progenitor_0
Myeov2	1.27E-23	-0.311961619	0.925	0.955	1.84E-19	0	Mesenchymal progenitor_0
2010107E04Rik	2.22E-23	-0.367954358	0.677	0.803	3.23E-19	0	Mesenchymal progenitor_0
Snhg18	4.41E-23	-0.423434724	0.614	0.751	6.42E-19	0	Mesenchymal progenitor_0
Pabpc1	9.95E-23	0.31557815	0.949	0.874	1.45E-18	0	Mesenchymal progenitor_0
Gm10126	2.10E-22	-0.259240409	0.02	0.162	3.06E-18	0	Mesenchymal progenitor_0
Gm10116	2.86E-21	0.384089766	0.711	0.459	4.17E-17	0	Mesenchymal progenitor_0
Ppp1r14b	3.08E-21	0.313976365	0.9	0.76	4.48E-17	0	Mesenchymal progenitor_0
mt-Nd3	4.63E-21	-0.377211169	0.227	0.437	6.75E-17	0	Mesenchymal progenitor_0
Egr1	4.21E-20	-0.497661068	0.451	0.636	6.13E-16	0	Mesenchymal progenitor_0
Nisch	1.55E-19	-0.373670847	0.612	0.732	2.25E-15	0	Mesenchymal progenitor_0
Polr2l	7.41E-19	-0.35204875	0.651	0.763	1.08E-14	0	Mesenchymal progenitor_0
Arpc1b	1.95E-18	0.275588297	0.952	0.874	2.84E-14	0	Mesenchymal progenitor_0
Myl9	3.07E-18	-0.377165754	0.895	0.929	4.47E-14	0	Mesenchymal progenitor_0
Gm10073	1.46E-17	-0.283196163	0.783	0.845	2.12E-13	0	Mesenchymal progenitor_0
Dstn	1.73E-17	-0.327770863	0.888	0.905	2.52E-13	0	Mesenchymal progenitor_0
Snrpg	3.12E-17	-0.271977591	0.868	0.898	4.54E-13	0	Mesenchymal progenitor_0
Wisp1	7.65E-17	0.288383536	0.871	0.731	1.11E-12	0	Mesenchymal progenitor_0
Eif4ebp1	1.06E-16	0.256625408	0.969	0.872	1.54E-12	0	Mesenchymal progenitor_0
Gm8186	1.67E-16	-0.358144879	0.296	0.475	2.43E-12	0	Mesenchymal progenitor_0
Prss23	1.88E-16	0.377746359	0.795	0.611	2.74E-12	0	Mesenchymal progenitor_0

Col12a1	3.13E-16	-0.322296157	0.948	0.962	4.57E-12	0	Mesenchymal progenitor_0
Pet100	3.87E-16	-0.376075288	0.349	0.515	5.64E-12	0	Mesenchymal progenitor_0
Fads3	1.35E-15	0.325835653	0.625	0.434	1.97E-11	0	Mesenchymal progenitor_0
Eif3k	1.76E-15	0.250714658	0.904	0.813	2.57E-11	0	Mesenchymal progenitor_0
Itm2a	2.84E-15	-0.473506066	0.721	0.801	4.14E-11	0	Mesenchymal progenitor_0
Rpl6l	3.51E-15	0.286029517	0.534	0.321	5.11E-11	0	Mesenchymal progenitor_0
Psap	5.51E-15	-0.311578496	0.869	0.903	8.02E-11	0	Mesenchymal progenitor_0
Apoe	2.68E-14	-0.289910208	0.106	0.254	3.90E-10	0	Mesenchymal progenitor_0
Tomm7	7.55E-14	-0.26134511	0.782	0.827	1.10E-09	0	Mesenchymal progenitor_0
Gm9493	1.18E-13	0.250211536	0.502	0.295	1.71E-09	0	Mesenchymal progenitor_0
Nme1	1.38E-13	0.277551749	0.855	0.746	2.02E-09	0	Mesenchymal progenitor_0
Usp50	2.57E-13	-0.279291645	0.771	0.807	3.75E-09	0	Mesenchymal progenitor_0
Dync1i2	6.26E-13	-0.316485379	0.401	0.549	9.11E-09	0	Mesenchymal progenitor_0
Pgk1	8.44E-13	0.293639999	0.63	0.458	1.23E-08	0	Mesenchymal progenitor_0
Toporsos	3.06E-12	-0.311029711	0.239	0.387	4.45E-08	0	Mesenchymal progenitor_0
Vps8	7.70E-11	-0.265841485	0.073	0.181	1.12E-06	0	Mesenchymal progenitor_0
Ssbp3	2.60E-10	-0.276452373	0.253	0.389	3.79E-06	0	Mesenchymal progenitor_0
PISD	3.27E-10	-0.257174254	0.125	0.245	4.77E-06	0	Mesenchymal progenitor_0
Ptrf	3.46E-10	-0.267163718	0.758	0.794	5.04E-06	0	Mesenchymal progenitor_0
Hk2	4.66E-10	-0.294694312	0.144	0.266	6.79E-06	0	Mesenchymal progenitor_0
Mmp23	5.33E-10	-0.275549541	0.568	0.653	7.76E-06	0	Mesenchymal progenitor_0
Clec11a	6.96E-10	-0.313874442	0.286	0.415	1.01E-05	0	Mesenchymal progenitor_0
Maged1	1.34E-09	-0.254880864	0.688	0.762	1.95E-05	0	Mesenchymal progenitor_0
Bicc1	2.46E-09	-0.266436189	0.607	0.693	3.59E-05	0	Mesenchymal progenitor_0

Pam	5.37E-09	-0.354561798	0.326	0.434	7.82E-05	0	Mesenchymal progenitor_0
Tspan31	6.62E-09	-0.255431594	0.287	0.406	9.65E-05	0	Mesenchymal progenitor_0
Fgfr2	7.03E-09	-0.266763609	0.232	0.361	0.000102449	0	Mesenchymal progenitor_0
Nrp2	7.07E-09	-0.313048687	0.628	0.712	0.000103025	0	Mesenchymal progenitor_0
Ctsb	1.02E-08	-0.32681173	0.768	0.798	0.000149112	0	Mesenchymal progenitor_0
Igfbp4	4.17E-08	-0.329048582	0.699	0.762	0.000606706	0	Mesenchymal progenitor_0
Ier2	5.06E-08	-0.3672142	0.428	0.515	0.000736288	0	Mesenchymal progenitor_0
Zfp36	5.80E-08	-0.250839957	0.144	0.249	0.000844151	0	Mesenchymal progenitor_0
Ptprs	7.26E-08	-0.282461558	0.47	0.56	0.00105798	0	Mesenchymal progenitor_0
Gpx3	8.32E-08	-0.371653044	0.719	0.755	0.001212437	0	Mesenchymal progenitor_0
Tmem86a	1.16E-07	-0.305359849	0.303	0.418	0.001686644	0	Mesenchymal progenitor_0
Kcnk2	2.22E-07	-0.259215324	0.109	0.204	0.003230612	0	Mesenchymal progenitor_0
Acta2	2.67E-07	-0.266579888	0.975	0.962	0.003890236	0	Mesenchymal progenitor_0
Mef2c	3.00E-07	-0.303059487	0.322	0.415	0.004364808	0	Mesenchymal progenitor_0
Rps29	1.51E-145	-0.642491626	1	1	2.20E-141	1	Mesenchymal progenitor_1
Rps28	2.98E-145	-0.906632805	0.987	0.997	4.33E-141	1	Mesenchymal progenitor_1
Rpl41	1.45E-111	-0.473054181	1	1	2.10E-107	1	Mesenchymal progenitor_1
Rpl38	8.22E-106	-0.63902187	0.994	1	1.20E-101	1	Mesenchymal progenitor_1
Rps27	1.20E-95	-0.492563213	1	1	1.75E-91	1	Mesenchymal progenitor_1
Gm10076	2.27E-76	-0.469791212	0.996	0.995	3.31E-72	1	Mesenchymal progenitor_1
Rpl37a	4.29E-69	-0.373975444	1	1	6.25E-65	1	Mesenchymal progenitor_1
Gm26917	6.57E-68	0.761804557	0.919	0.619	9.57E-64	1	Mesenchymal progenitor_1
Gm8730	9.80E-66	0.633929706	0.989	0.949	1.43E-61	1	Mesenchymal progenitor_1
Rpl36	3.77E-62	-0.443541267	0.985	0.997	5.49E-58	1	Mesenchymal progenitor_1

Calr	1.89E-49	0.669204578	0.913	0.654	2.76E-45	1	Mesenchymal progenitor_1
Atp5k	7.51E-49	-0.547453998	0.851	0.973	1.09E-44	1	Mesenchymal progenitor_1
mt-Nd2	1.19E-44	-0.611830881	0.718	0.907	1.73E-40	1	Mesenchymal progenitor_1
Rpl37	2.90E-44	-0.343020204	1	1	4.22E-40	1	Mesenchymal progenitor_1
Rpl39	2.59E-42	-0.441887204	0.985	0.994	3.77E-38	1	Mesenchymal progenitor_1
Rps21	4.21E-41	-0.43121603	0.923	0.987	6.14E-37	1	Mesenchymal progenitor_1
Rps2	1.76E-40	0.428195133	0.998	0.974	2.56E-36	1	Mesenchymal progenitor_1
mt-Cytb	5.81E-39	0.297318762	1	1	8.46E-35	1	Mesenchymal progenitor_1
Ibsp	3.78E-38	0.891479026	0.782	0.418	5.51E-34	1	Mesenchymal progenitor_1
Nmt1	3.32E-37	-0.709653643	0.185	0.526	4.83E-33	1	Mesenchymal progenitor_1
Malat1	3.99E-33	-0.481248611	1	1	5.81E-29	1	Mesenchymal progenitor_1
Sertad2	4.93E-32	-0.673933966	0.217	0.526	7.18E-28	1	Mesenchymal progenitor_1
Ndufa3	4.11E-30	-0.428622117	0.831	0.932	5.99E-26	1	Mesenchymal progenitor_1
Snhg9	1.34E-28	-0.565949577	0.047	0.304	1.95E-24	1	Mesenchymal progenitor_1
mt-Nd4	1.86E-28	0.305347846	0.998	0.995	2.70E-24	1	Mesenchymal progenitor_1
Eif2s2	4.13E-27	-0.484357905	0.742	0.878	6.01E-23	1	Mesenchymal progenitor_1
mt-Atp6	4.29E-27	0.255561834	1	1	6.24E-23	1	Mesenchymal progenitor_1
Hes1	1.92E-26	-0.692887893	0.343	0.611	2.79E-22	1	Mesenchymal progenitor_1
Sec61g	2.94E-26	-0.308900896	0.968	0.99	4.28E-22	1	Mesenchymal progenitor_1
Rpl15	7.87E-26	0.40382244	0.949	0.859	1.15E-21	1	Mesenchymal progenitor_1
Usmg5	8.64E-26	-0.430650222	0.71	0.844	1.26E-21	1	Mesenchymal progenitor_1
mt-Nd3	1.89E-25	-0.561925474	0.166	0.444	2.75E-21	1	Mesenchymal progenitor_1
Pfn1	8.20E-24	0.268254804	1	0.989	1.19E-19	1	Mesenchymal progenitor_1
Lmo4	1.50E-23	-0.55461179	0.469	0.703	2.19E-19	1	Mesenchymal progenitor_1

Nme2	1.54E-22	0.535859432	0.446	0.206	2.24E-18	1	Mesenchymal progenitor_1
Myeov2	1.37E-20	-0.356810501	0.846	0.916	1.99E-16	1	Mesenchymal progenitor_1
Rpl29	3.01E-20	0.431918391	0.704	0.492	4.38E-16	1	Mesenchymal progenitor_1
Polr2l	4.93E-20	-0.496261795	0.467	0.677	7.18E-16	1	Mesenchymal progenitor_1
Rps27rt	2.51E-18	-0.47734203	0.188	0.42	3.65E-14	1	Mesenchymal progenitor_1
2010107E04Rik	2.44E-17	-0.416123883	0.582	0.746	3.55E-13	1	Mesenchymal progenitor_1
Gm10260	7.46E-17	0.25355811	0.994	0.977	1.09E-12	1	Mesenchymal progenitor_1
Egr1	8.98E-17	-0.498915733	0.388	0.605	1.31E-12	1	Mesenchymal progenitor_1
Igkc	1.17E-16	1.11909222	0.298	0.109	1.70E-12	1	Mesenchymal progenitor_1
Rplp0	1.80E-16	0.250180379	0.998	0.977	2.62E-12	1	Mesenchymal progenitor_1
Crip1	5.22E-16	-0.490150925	0.81	0.899	7.61E-12	1	Mesenchymal progenitor_1
Snhg18	7.40E-16	-0.368138629	0.411	0.622	1.08E-11	1	Mesenchymal progenitor_1
Fos	2.41E-15	-0.484036147	0.586	0.744	3.50E-11	1	Mesenchymal progenitor_1
Gm10116	2.58E-15	0.406272534	0.525	0.315	3.76E-11	1	Mesenchymal progenitor_1
Pet100	4.87E-15	-0.43410105	0.331	0.535	7.10E-11	1	Mesenchymal progenitor_1
Arpc1b	6.90E-15	0.271246591	0.913	0.831	1.01E-10	1	Mesenchymal progenitor_1
Rbp4	1.70E-14	0.507591276	0.226	0.069	2.48E-10	1	Mesenchymal progenitor_1
Manf	2.40E-14	0.437078151	0.606	0.429	3.50E-10	1	Mesenchymal progenitor_1
Gm10263	2.55E-14	-0.314931254	0.024	0.158	3.71E-10	1	Mesenchymal progenitor_1
Hspa5	1.23E-13	0.427373925	0.644	0.468	1.80E-09	1	Mesenchymal progenitor_1
Gm10126	1.78E-13	-0.272242303	0.017	0.135	2.59E-09	1	Mesenchymal progenitor_1
Ppp1r14b	1.55E-12	0.355974635	0.708	0.572	2.26E-08	1	Mesenchymal progenitor_1
Pdia3	6.31E-12	0.358542964	0.395	0.217	9.19E-08	1	Mesenchymal progenitor_1
Gstp1	3.51E-11	0.369614756	0.514	0.349	5.11E-07	1	Mesenchymal progenitor_1

Psap	3.59E-11	-0.315206077	0.808	0.899	5.23E-07	1	Mesenchymal progenitor_1
Canx	3.87E-11	0.280775387	0.78	0.63	5.63E-07	1	Mesenchymal progenitor_1
Maob	1.18E-10	-0.396376019	0.226	0.386	1.72E-06	1	Mesenchymal progenitor_1
Esm1	1.76E-10	0.333600577	0.109	0.019	2.56E-06	1	Mesenchymal progenitor_1
Dync1i2	3.05E-10	-0.328228337	0.369	0.534	4.44E-06	1	Mesenchymal progenitor_1
Ptrf	3.05E-10	-0.254306562	0.701	0.812	4.44E-06	1	Mesenchymal progenitor_1
Cdkn1a	5.65E-10	0.293651338	0.593	0.423	8.23E-06	1	Mesenchymal progenitor_1
Eif4ebp1	6.05E-10	0.302131933	0.791	0.683	8.82E-06	1	Mesenchymal progenitor_1
Actn4	8.11E-10	-0.290879747	0.693	0.781	1.18E-05	1	Mesenchymal progenitor_1
Fosb	8.77E-10	-0.313915039	0.196	0.363	1.28E-05	1	Mesenchymal progenitor_1
Pdia6	9.48E-10	0.345463486	0.561	0.394	1.38E-05	1	Mesenchymal progenitor_1
Snrpg	1.01E-09	-0.286629438	0.682	0.781	1.47E-05	1	Mesenchymal progenitor_1
Mmp17	1.31E-09	-0.380118155	0.234	0.383	1.91E-05	1	Mesenchymal progenitor_1
Mrps6	2.06E-09	0.338597051	0.655	0.524	3.00E-05	1	Mesenchymal progenitor_1
Cycs	2.56E-09	0.281542651	0.52	0.367	3.73E-05	1	Mesenchymal progenitor_1
Igfbp5	2.57E-09	-0.511039231	0.48	0.627	3.75E-05	1	Mesenchymal progenitor_1
Nisch	2.74E-09	-0.28964228	0.508	0.633	3.99E-05	1	Mesenchymal progenitor_1
Myh9	3.05E-09	-0.297927848	0.58	0.693	4.44E-05	1	Mesenchymal progenitor_1
Gm10073	3.08E-09	-0.297772243	0.482	0.622	4.49E-05	1	Mesenchymal progenitor_1
Toporsos	3.14E-09	-0.299457231	0.162	0.315	4.57E-05	1	Mesenchymal progenitor_1
Pgk1	4.05E-09	0.299815795	0.46	0.299	5.90E-05	1	Mesenchymal progenitor_1
Gm10020	4.91E-09	0.275644896	0.371	0.222	7.14E-05	1	Mesenchymal progenitor_1
Grn	8.72E-09	-0.355504869	0.409	0.543	0.000127051	1	Mesenchymal progenitor_1
Bst2	9.37E-09	0.298973785	0.275	0.141	0.000136471	1	Mesenchymal progenitor_1

Lox	2.58E-08	0.263093578	0.957	0.912	0.000375941	1	Mesenchymal progenitor_1
S100a16	2.86E-08	0.250697106	0.684	0.556	0.000416027	1	Mesenchymal progenitor_1
Pam	5.61E-08	-0.382967875	0.35	0.477	0.000816387	1	Mesenchymal progenitor_1
Hp	6.76E-08	0.451355905	0.75	0.635	0.000983823	1	Mesenchymal progenitor_1
Thbs2	7.03E-08	-0.25325947	0.763	0.836	0.001023842	1	Mesenchymal progenitor_1
Hnrnpa3	1.10E-07	0.257114428	0.729	0.64	0.00159505	1	Mesenchymal progenitor_1
Usp50	1.10E-07	-0.279655592	0.648	0.744	0.001597243	1	Mesenchymal progenitor_1
Desi1	1.12E-07	-0.267445181	0.128	0.246	0.001625192	1	Mesenchymal progenitor_1
Timp1	1.12E-07	0.338133993	0.699	0.608	0.001636427	1	Mesenchymal progenitor_1
H2-D1	1.31E-07	0.29676192	0.934	0.912	0.001912458	1	Mesenchymal progenitor_1
Gpc1	2.20E-07	-0.310544713	0.309	0.437	0.003202602	1	Mesenchymal progenitor_1
mt-Atp8	2.38E-07	-0.265919001	0.476	0.6	0.003472998	1	Mesenchymal progenitor_1
Gm8186	2.46E-07	-0.266438663	0.205	0.338	0.003580293	1	Mesenchymal progenitor_1
1110008P14Rik	3.13E-07	0.259606935	0.388	0.257	0.00456009	1	Mesenchymal progenitor_1
Ogn	3.83E-07	-0.2545062	0.748	0.828	0.005576357	1	Mesenchymal progenitor_1
Ccng1	4.05E-07	0.283576989	0.514	0.391	0.00590233	1	Mesenchymal progenitor_1
Adgra2	5.96E-07	-0.266300939	0.164	0.286	0.008678668	1	Mesenchymal progenitor_1
Rps28	4.29E-151	-0.956225658	0.991	1	6.24E-147	2	Mesenchymal progenitor_2
Rps29	3.01E-136	-0.640079528	1	1	4.38E-132	2	Mesenchymal progenitor_2
Rpl38	5.59E-110	-0.594351119	1	1	8.14E-106	2	Mesenchymal progenitor_2
Rpl41	1.80E-93	-0.406585246	1	1	2.63E-89	2	Mesenchymal progenitor_2
Rps27	8.51E-91	-0.448457349	1	1	1.24E-86	2	Mesenchymal progenitor_2
Gm8730	3.45E-74	0.653508256	0.99	0.968	5.02E-70	2	Mesenchymal progenitor_2
Gm26917	9.32E-72	0.71149589	0.937	0.713	1.36E-67	2	Mesenchymal progenitor_2

Rps2	5.91E-68	0.517040691	1	0.983	8.60E-64	2	Mesenchymal progenitor_2
Rpl37a	2.18E-62	-0.321838706	1	1	3.17E-58	2	Mesenchymal progenitor_2
Gm10076	1.42E-56	-0.369800729	1	1	2.06E-52	2	Mesenchymal progenitor_2
mt-Nd2	1.35E-52	-0.658348186	0.751	0.939	1.96E-48	2	Mesenchymal progenitor_2
Rps21	5.30E-51	-0.437947257	0.97	0.989	7.71E-47	2	Mesenchymal progenitor_2
Nmt1	1.13E-45	-0.688391931	0.235	0.626	1.64E-41	2	Mesenchymal progenitor_2
Ibsp	3.59E-44	0.786232674	0.697	0.315	5.23E-40	2	Mesenchymal progenitor_2
Atp5k	6.45E-44	-0.523588239	0.861	0.946	9.39E-40	2	Mesenchymal progenitor_2
Eif2s2	7.38E-44	-0.531792723	0.875	0.952	1.07E-39	2	Mesenchymal progenitor_2
Rpl36	2.63E-42	-0.334646999	0.99	1	3.83E-38	2	Mesenchymal progenitor_2
Rpl15	1.88E-41	0.470526643	0.972	0.909	2.74E-37	2	Mesenchymal progenitor_2
mt-Cytb	5.39E-39	0.305245121	1	1	7.85E-35	2	Mesenchymal progenitor_2
Rpl37	5.77E-39	-0.297830081	1	1	8.41E-35	2	Mesenchymal progenitor_2
Calr	6.19E-39	0.550998351	0.944	0.799	9.01E-35	2	Mesenchymal progenitor_2
Nme2	5.44E-37	0.640315733	0.631	0.322	7.92E-33	2	Mesenchymal progenitor_2
Rpl39	1.53E-36	-0.397531136	0.993	0.998	2.23E-32	2	Mesenchymal progenitor_2
Snhg9	2.37E-35	-0.508096766	0.064	0.372	3.45E-31	2	Mesenchymal progenitor_2
Sec61g	4.58E-34	-0.306810997	0.991	0.991	6.67E-30	2	Mesenchymal progenitor_2
Malat1	4.07E-31	-0.399642084	1	1	5.93E-27	2	Mesenchymal progenitor_2
Usmg5	1.16E-30	-0.44373862	0.776	0.916	1.69E-26	2	Mesenchymal progenitor_2
Sertad2	8.34E-30	-0.566271261	0.381	0.678	1.21E-25	2	Mesenchymal progenitor_2
Rplp0	4.47E-27	0.321604291	0.998	0.998	6.51E-23	2	Mesenchymal progenitor_2
Lmo4	7.88E-26	-0.545925924	0.428	0.7	1.15E-21	2	Mesenchymal progenitor_2
mt-Atp6	4.84E-25	0.261847822	1	1	7.05E-21	2	Mesenchymal progenitor_2

Ndufa3	8.25E-25	-0.3787420 67	0.82 6	0.92 4	1.20E-20	2	Mesenchymal progenitor_2
Rpl29	1.24E-24	0.43661767 6	0.76 5	0.56 8	1.81E-20	2	Mesenchymal progenitor_2
mt-Nd4	3.06E-23	0.29334846 8	1	0.99 8	4.45E-19	2	Mesenchymal progenitor_2
Fos	4.40E-23	-0.6199480 9	0.43 5	0.69 8	6.41E-19	2	Mesenchymal progenitor_2
Ftl1	1.33E-22	0.25157701 3	1	0.99 8	1.93E-18	2	Mesenchymal progenitor_2
Rps18	3.11E-22	0.25813614 4	1	0.99 6	4.53E-18	2	Mesenchymal progenitor_2
Igkc	2.07E-21	0.89427937 4	0.36 2	0.12 3	3.02E-17	2	Mesenchymal progenitor_2
Gm10260	2.14E-21	0.28272503 3	0.99 5	0.98 7	3.12E-17	2	Mesenchymal progenitor_2
Snrpg	4.64E-21	-0.3550118 33	0.70 1	0.84 5	6.76E-17	2	Mesenchymal progenitor_2
Rps27rt	4.79E-21	-0.3986832 64	0.25 2	0.51 2	6.97E-17	2	Mesenchymal progenitor_2
Hes1	5.97E-21	-0.5664358 39	0.44 7	0.65 9	8.69E-17	2	Mesenchymal progenitor_2
Nisch	1.13E-20	-0.4042828 77	0.53 6	0.75 6	1.64E-16	2	Mesenchymal progenitor_2
mt-Nd3	1.45E-20	-0.4241519 22	0.20 5	0.46 7	2.12E-16	2	Mesenchymal progenitor_2
Ogn	1.13E-18	-0.4973547 37	0.68 3	0.84 9	1.65E-14	2	Mesenchymal progenitor_2
Gm10263	3.08E-18	-0.2923295 62	0.03 5	0.20 3	4.48E-14	2	Mesenchymal progenitor_2
Polr2l	3.31E-18	-0.4137287 87	0.55	0.76 2	4.82E-14	2	Mesenchymal progenitor_2
Gm10073	3.62E-18	-0.3536206 99	0.60 5	0.77 5	5.27E-14	2	Mesenchymal progenitor_2
Prss23	1.08E-17	0.39707429 4	0.95 5	0.92 6	1.58E-13	2	Mesenchymal progenitor_2
Myl9	2.32E-17	-0.2591694 26	0.99	0.99 8	3.38E-13	2	Mesenchymal progenitor_2
Egr1	4.58E-17	-0.4541898 96	0.28 9	0.51	6.68E-13	2	Mesenchymal progenitor_2
2010107E04 Rik	1.24E-16	-0.3014659 81	0.63 1	0.80 1	1.81E-12	2	Mesenchymal progenitor_2
Snhg18	1.58E-16	-0.4044956 96	0.50 6	0.71 1	2.31E-12	2	Mesenchymal progenitor_2
Timp1	1.98E-15	0.37248687 3	0.91	0.83 8	2.88E-11	2	Mesenchymal progenitor_2
Myeov2	4.30E-15	-0.2759549 39	0.87 3	0.92 7	6.26E-11	2	Mesenchymal progenitor_2

Lox	6.73E-15	0.32704086	0.988	0.968	9.80E-11	2	Mesenchymal progenitor_2
Dstn	2.85E-14	-0.274158981	0.955	0.981	4.15E-10	2	Mesenchymal progenitor_2
Vamp5	3.44E-14	0.40675999	0.685	0.531	5.02E-10	2	Mesenchymal progenitor_2
Psap	9.32E-14	-0.269416258	0.864	0.926	1.36E-09	2	Mesenchymal progenitor_2
Hnrnpa3	2.89E-13	0.298824588	0.798	0.661	4.21E-09	2	Mesenchymal progenitor_2
Grn	4.98E-13	-0.370476245	0.424	0.601	7.25E-09	2	Mesenchymal progenitor_2
Rtn4	7.58E-13	0.28457748	0.885	0.782	1.10E-08	2	Mesenchymal progenitor_2
Gm10116	1.90E-12	0.345270231	0.569	0.4	2.77E-08	2	Mesenchymal progenitor_2
Arpc1b	4.91E-12	0.290060699	0.877	0.797	7.15E-08	2	Mesenchymal progenitor_2
Apoe	8.23E-12	-0.272591059	0.09	0.244	1.20E-07	2	Mesenchymal progenitor_2
Wisp1	8.26E-12	0.279021634	0.868	0.747	1.20E-07	2	Mesenchymal progenitor_2
Actn4	9.26E-12	-0.266255649	0.859	0.935	1.35E-07	2	Mesenchymal progenitor_2
Tomm7	1.02E-11	-0.271045623	0.703	0.831	1.48E-07	2	Mesenchymal progenitor_2
Lasp1	1.09E-11	-0.276386051	0.659	0.784	1.59E-07	2	Mesenchymal progenitor_2
Dkk3	1.13E-11	-0.32020916	0.376	0.566	1.65E-07	2	Mesenchymal progenitor_2
Olfml3	1.56E-11	-0.340914006	0.341	0.523	2.27E-07	2	Mesenchymal progenitor_2
Mmp23	2.23E-11	-0.319195071	0.501	0.693	3.25E-07	2	Mesenchymal progenitor_2
Ndufv3	3.16E-11	-0.261489908	0.77	0.868	4.61E-07	2	Mesenchymal progenitor_2
Manf	4.20E-11	0.32635699	0.701	0.559	6.11E-07	2	Mesenchymal progenitor_2
Bmp1	4.29E-11	0.284823867	0.687	0.521	6.24E-07	2	Mesenchymal progenitor_2
Pet100	7.21E-11	-0.28940651	0.355	0.536	1.05E-06	2	Mesenchymal progenitor_2
Col4a2	1.45E-10	0.318071465	0.805	0.685	2.10E-06	2	Mesenchymal progenitor_2
Pdia3	1.47E-10	0.327927525	0.454	0.3	2.14E-06	2	Mesenchymal progenitor_2
Eif4ebp1	1.60E-10	0.256998783	0.934	0.885	2.33E-06	2	Mesenchymal progenitor_2

Nupr1	2.44E-10	0.278981537	0.965	0.912	3.56E-06	2	Mesenchymal progenitor_2
Cxcl12	3.96E-10	-0.345251499	0.715	0.832	5.77E-06	2	Mesenchymal progenitor_2
Ap3s1	4.25E-10	0.262133999	0.84	0.78	6.19E-06	2	Mesenchymal progenitor_2
Itm2a	4.63E-10	-0.407161144	0.329	0.499	6.74E-06	2	Mesenchymal progenitor_2
Emilin1	8.57E-10	-0.319836554	0.327	0.48	1.25E-05	2	Mesenchymal progenitor_2
Igfbp7	8.63E-10	0.302713638	0.99	0.978	1.26E-05	2	Mesenchymal progenitor_2
Gm10020	9.56E-10	0.287607723	0.41	0.264	1.39E-05	2	Mesenchymal progenitor_2
Ptprs	1.03E-09	-0.26994172	0.41	0.577	1.50E-05	2	Mesenchymal progenitor_2
Ppp1r14b	1.09E-09	0.292896341	0.783	0.685	1.59E-05	2	Mesenchymal progenitor_2
Sept7	2.17E-09	0.257499847	0.807	0.693	3.16E-05	2	Mesenchymal progenitor_2
Toporsos	6.82E-09	-0.252873368	0.193	0.346	9.93E-05	2	Mesenchymal progenitor_2
Tnfrsf12a	7.00E-09	0.254750361	0.683	0.566	0.000101906	2	Mesenchymal progenitor_2
Sap18	8.07E-09	0.301169605	0.59	0.439	0.000117533	2	Mesenchymal progenitor_2
Dync1i2	9.52E-09	-0.26154429	0.424	0.592	0.000138704	2	Mesenchymal progenitor_2
Aldh2	1.07E-08	-0.264271135	0.315	0.477	0.000156135	2	Mesenchymal progenitor_2
Col11a1	1.94E-08	-0.30947785	0.43	0.57	0.000283122	2	Mesenchymal progenitor_2
mt-Atp8	2.77E-08	-0.251992927	0.557	0.687	0.000402988	2	Mesenchymal progenitor_2
Cyb5r1	2.98E-08	0.258190392	0.424	0.291	0.000433542	2	Mesenchymal progenitor_2
Cox17	3.05E-08	-0.270938741	0.56	0.7	0.000444096	2	Mesenchymal progenitor_2
Tbx18	3.11E-08	0.336097965	0.303	0.173	0.000453323	2	Mesenchymal progenitor_2
Lgals3bp	3.15E-08	-0.264557248	0.172	0.307	0.000458589	2	Mesenchymal progenitor_2
Nexn	4.49E-08	-0.294413951	0.32	0.469	0.00065407	2	Mesenchymal progenitor_2
Vamp8	5.82E-08	0.270967846	0.757	0.663	0.00084696	2	Mesenchymal progenitor_2
Hist2h2aa1	1.00E-07	0.267445576	0.249	0.132	0.001461918	2	Mesenchymal progenitor_2

Pam	1.10E-07	-0.2999647<br>47	0.36<br>7	0.52<br>1	0.00159613<br>1	2	Mesenchymal<br>progenitor_2
Smtn	1.63E-07	-0.2530475<br>56	0.23<br>1	0.36<br>7	0.00237634<br>9	2	Mesenchymal<br>progenitor_2
Gpc1	2.21E-07	-0.2565323<br>48	0.30<br>1	0.45<br>3	0.00322334<br>8	2	Mesenchymal<br>progenitor_2
Maob	4.41E-07	-0.2804184<br>97	0.16<br>7	0.29<br>1	0.00642618<br>1	2	Mesenchymal<br>progenitor_2
Rps28	1.58E-97	-0.9331412<br>32	0.99<br>8	1	2.30E-93	3	Osteoblast<br>progenitor_3
Rps29	1.52E-93	-0.7009035	1	1	2.22E-89	3	Osteoblast<br>progenitor_3
Rpl41	1.51E-82	-0.4957822<br>16	1	1	2.19E-78	3	Osteoblast<br>progenitor_3
Rpl38	6.89E-81	-0.6431625<br>92	1	1	1.00E-76	3	Osteoblast<br>progenitor_3
Rpl36	7.82E-61	-0.4595128<br>79	0.99<br>7	1	1.14E-56	3	Osteoblast<br>progenitor_3
Gm8730	9.88E-61	0.66487063<br>6	1	0.99	1.44E-56	3	Osteoblast<br>progenitor_3
Rps27	3.51E-56	-0.4161044<br>38	1	1	5.11E-52	3	Osteoblast<br>progenitor_3
Gm10076	1.17E-55	-0.4030433<br>4	1	1	1.70E-51	3	Osteoblast<br>progenitor_3
Gm26917	2.76E-54	0.75144119<br>8	0.97<br>5	0.79<br>5	4.02E-50	3	Osteoblast<br>progenitor_3
Snhg9	1.60E-50	-0.7876897<br>25	0.11<br>9	0.59	2.32E-46	3	Osteoblast<br>progenitor_3
Rps2	3.49E-50	0.41632436<br>6	0.99<br>8	0.99<br>5	5.08E-46	3	Osteoblast<br>progenitor_3
Rpl37	2.59E-48	-0.3401092<br>99	1	0.99<br>5	3.77E-44	3	Osteoblast<br>progenitor_3
Rpl15	4.96E-46	0.46474627<br>9	0.98<br>9	0.97<br>6	7.23E-42	3	Osteoblast<br>progenitor_3
Rpl37a	3.44E-45	-0.3184781<br>95	1	1	5.00E-41	3	Osteoblast<br>progenitor_3
Eif2s2	5.64E-40	-0.5686537<br>24	0.96<br>2	0.98	8.21E-36	3	Osteoblast<br>progenitor_3
Atp5k	7.53E-40	-0.6770160<br>8	0.88<br>8	0.96<br>1	1.10E-35	3	Osteoblast<br>progenitor_3
Rps21	1.02E-37	-0.3950150<br>42	0.99<br>5	1	1.49E-33	3	Osteoblast<br>progenitor_3
mt-Nd2	1.91E-36	-0.6891543<br>04	0.84<br>3	0.95<br>6	2.79E-32	3	Osteoblast<br>progenitor_3
Nme2	3.13E-36	0.68867788<br>5	0.86<br>9	0.50<br>2	4.56E-32	3	Osteoblast<br>progenitor_3
Rplp0	4.66E-34	0.36804686<br>8	1	1	6.78E-30	3	Osteoblast<br>progenitor_3

Nmt1	3.81E-33	-0.698514786	0.31	0.698	5.55E-29	3	Osteoblast progenitor_3
Rpl39	1.29E-31	-0.40315516	1	1	1.88E-27	3	Osteoblast progenitor_3
Ftl1	1.52E-30	0.445961407	1	1	2.21E-26	3	Osteoblast progenitor_3
Rpl29	7.75E-29	0.531019597	0.924	0.8	1.13E-24	3	Osteoblast progenitor_3
Lmo4	8.04E-29	-0.694818076	0.601	0.829	1.17E-24	3	Osteoblast progenitor_3
Sec61g	9.62E-24	-0.307414355	0.992	1	1.40E-19	3	Osteoblast progenitor_3
Sertad2	2.96E-22	-0.520882979	0.438	0.702	4.31E-18	3	Osteoblast progenitor_3
Ndufa3	1.16E-21	-0.519158224	0.818	0.932	1.69E-17	3	Osteoblast progenitor_3
Rps27rt	1.97E-21	-0.536142069	0.503	0.766	2.86E-17	3	Osteoblast progenitor_3
Timp1	1.76E-20	0.557781968	0.975	0.898	2.56E-16	3	Osteoblast progenitor_3
Malat1	1.96E-19	-0.490886513	1	1	2.86E-15	3	Osteoblast progenitor_3
Hes1	2.77E-19	-0.668451745	0.396	0.663	4.03E-15	3	Osteoblast progenitor_3
Lox	1.06E-18	0.528173728	0.948	0.824	1.55E-14	3	Osteoblast progenitor_3
Smpd3	1.34E-17	-0.78297336	0.169	0.424	1.95E-13	3	Osteoblast progenitor_3
Snrpg	1.67E-17	-0.347515234	0.91	0.961	2.43E-13	3	Osteoblast progenitor_3
Usmg5	4.28E-17	-0.410118881	0.843	0.922	6.23E-13	3	Osteoblast progenitor_3
Snhg18	4.69E-17	-0.496171438	0.666	0.829	6.83E-13	3	Osteoblast progenitor_3
Gm10263	2.04E-16	-0.390906178	0.095	0.327	2.97E-12	3	Osteoblast progenitor_3
Gm10126	9.33E-16	-0.273122118	0.03	0.19	1.36E-11	3	Osteoblast progenitor_3
Gm10709	1.28E-15	0.280169365	0.975	0.971	1.87E-11	3	Osteoblast progenitor_3
Gm10073	1.30E-15	-0.348854646	0.85	0.946	1.89E-11	3	Osteoblast progenitor_3
Gm10020	1.33E-15	0.433786896	0.744	0.449	1.94E-11	3	Osteoblast progenitor_3
Gm10116	1.54E-15	0.474687134	0.769	0.483	2.24E-11	3	Osteoblast progenitor_3
Chchd2	1.89E-14	0.289944553	0.984	0.951	2.76E-10	3	Osteoblast progenitor_3

Apoe	1.04E-13	-0.32490018	0.074	0.263	1.52E-09	3	Osteoblast progenitor_3
Itm2a	1.12E-13	-0.592288412	0.652	0.82	1.63E-09	3	Osteoblast progenitor_3
Ppp1r14b	7.87E-13	0.316477231	0.943	0.878	1.15E-08	3	Osteoblast progenitor_3
Pgk1	9.24E-13	0.442496392	0.684	0.468	1.35E-08	3	Osteoblast progenitor_3
Ybx1	1.32E-12	0.252235793	0.972	0.985	1.93E-08	3	Osteoblast progenitor_3
mt-Nd3	1.73E-12	-0.416739066	0.248	0.473	2.52E-08	3	Osteoblast progenitor_3
Igfbp4	1.79E-12	-0.604658087	0.72	0.883	2.60E-08	3	Osteoblast progenitor_3
Igkc	3.15E-12	0.371147446	0.403	0.132	4.59E-08	3	Osteoblast progenitor_3
Pabpc1	3.72E-12	0.270195043	0.964	0.976	5.41E-08	3	Osteoblast progenitor_3
Timp2	1.74E-11	-0.282725651	0.881	0.961	2.53E-07	3	Osteoblast progenitor_3
Gm8186	2.25E-11	-0.354064782	0.402	0.595	3.28E-07	3	Osteoblast progenitor_3
Psap	2.85E-11	-0.39885651	0.866	0.912	4.15E-07	3	Osteoblast progenitor_3
Fos	2.97E-11	-0.488419872	0.669	0.78	4.33E-07	3	Osteoblast progenitor_3
Rpl10-ps3	9.22E-11	0.290709834	0.557	0.259	1.34E-06	3	Osteoblast progenitor_3
Mef2c	2.62E-10	-0.461326239	0.541	0.707	3.82E-06	3	Osteoblast progenitor_3
Toporsos	2.67E-10	-0.342442785	0.203	0.395	3.90E-06	3	Osteoblast progenitor_3
Tpm1	3.53E-10	-0.287572695	0.967	0.985	5.14E-06	3	Osteoblast progenitor_3
Rpl6l	3.59E-10	0.306245452	0.703	0.459	5.22E-06	3	Osteoblast progenitor_3
Ogn	4.77E-10	-0.342156534	0.498	0.712	6.95E-06	3	Osteoblast progenitor_3
Eif4ebp1	6.08E-10	0.26720729	0.964	0.922	8.86E-06	3	Osteoblast progenitor_3
Wisp1	1.26E-09	0.388487604	0.799	0.566	1.84E-05	3	Osteoblast progenitor_3
Fgfr2	1.35E-09	-0.36497966	0.277	0.473	1.97E-05	3	Osteoblast progenitor_3
Cdsn	1.57E-09	0.457092521	0.691	0.493	2.28E-05	3	Osteoblast progenitor_3
Bag1	1.68E-09	0.309946326	0.886	0.741	2.45E-05	3	Osteoblast progenitor_3

Romo1	2.14E-09	-0.252959286	0.93	0.956	3.12E-05	3	Osteoblast progenitor_3
Aplp2	8.96E-09	-0.323469703	0.677	0.785	0.000130528	3	Osteoblast progenitor_3
Bmp1	9.13E-09	0.305320687	0.638	0.385	0.000132979	3	Osteoblast progenitor_3
Polr2l	1.02E-08	-0.354354889	0.646	0.727	0.000148093	3	Osteoblast progenitor_3
Fads3	1.14E-08	0.281031814	0.665	0.449	0.000166345	3	Osteoblast progenitor_3
Rheb	2.20E-08	0.296593371	0.718	0.498	0.0003206	3	Osteoblast progenitor_3
Pcolce	2.26E-08	-0.343155487	0.797	0.902	0.000328649	3	Osteoblast progenitor_3
Nip7	3.99E-08	-0.645853938	0.383	0.522	0.00058087	3	Osteoblast progenitor_3
Prss23	4.07E-08	0.366591361	0.527	0.298	0.000593481	3	Osteoblast progenitor_3
Cxcl12	4.19E-08	-0.429077388	0.478	0.629	0.000610003	3	Osteoblast progenitor_3
Nisch	5.33E-08	-0.322255625	0.606	0.717	0.000776406	3	Osteoblast progenitor_3
Fry	6.28E-08	-0.366527726	0.063	0.185	0.000914634	3	Osteoblast progenitor_3
Gm9493	7.95E-08	0.28900119	0.642	0.424	0.001158387	3	Osteoblast progenitor_3
Atp6v1g1	1.64E-07	0.272379321	0.943	0.863	0.002394036	3	Osteoblast progenitor_3
Spp1	1.70E-07	0.557019228	0.888	0.81	0.002469095	3	Osteoblast progenitor_3
Dpm3	1.74E-07	-0.307957526	0.623	0.737	0.002530038	3	Osteoblast progenitor_3
Tnn	3.70E-07	0.258809111	0.373	0.176	0.005387108	3	Osteoblast progenitor_3
Mylk	4.78E-07	-0.275563079	0.372	0.537	0.006955408	3	Osteoblast progenitor_3
Gstp1	5.19E-07	0.286869897	0.703	0.522	0.007558867	3	Osteoblast progenitor_3
Rps26-ps1	5.35E-07	-0.28148271	0.392	0.551	0.007791668	3	Osteoblast progenitor_3
Pdcd11	6.03E-07	-0.26235852	0.191	0.341	0.00877879	3	Osteoblast progenitor_3
Rps29	8.39E-108	-0.662292442	1	1	1.22E-103	4	Mesenchymal progenitor_4
Rps28	9.45E-101	-0.889762142	0.995	1	1.38E-96	4	Mesenchymal progenitor_4
Rpl38	6.62E-87	-0.632035583	0.995	1	9.65E-83	4	Mesenchymal progenitor_4

Rpl41	1.48E-80	-0.427444072	1	1	2.16E-76	4	Mesenchymal progenitor_4
Gm26917	2.47E-69	0.798171486	0.97	0.748	3.60E-65	4	Mesenchymal progenitor_4
Gm8730	2.28E-66	0.83101334	0.995	0.956	3.32E-62	4	Mesenchymal progenitor_4
Rps2	1.80E-60	0.603276639	1	0.985	2.62E-56	4	Mesenchymal progenitor_4
mt-Nd2	3.83E-54	-0.727358588	0.764	0.956	5.58E-50	4	Mesenchymal progenitor_4
Rpl37a	2.15E-53	-0.361727411	1	1	3.13E-49	4	Mesenchymal progenitor_4
Gm10076	5.03E-52	-0.404963906	1	1	7.32E-48	4	Mesenchymal progenitor_4
Atp5k	4.30E-48	-0.658301829	0.898	0.971	6.26E-44	4	Mesenchymal progenitor_4
Rps27	4.36E-44	-0.386987918	1	0.998	6.35E-40	4	Mesenchymal progenitor_4
Rpl15	1.05E-42	0.541502545	0.985	0.915	1.53E-38	4	Mesenchymal progenitor_4
Ibsp	9.08E-42	1.037109319	0.866	0.408	1.32E-37	4	Mesenchymal progenitor_4
Nmt1	2.32E-40	-0.780628508	0.283	0.687	3.38E-36	4	Mesenchymal progenitor_4
Rpl29	1.54E-35	0.618022196	0.844	0.607	2.24E-31	4	Mesenchymal progenitor_4
Rps21	4.87E-33	-0.405629261	0.97	0.993	7.10E-29	4	Mesenchymal progenitor_4
Sec61g	8.44E-33	-0.339623794	0.993	0.983	1.23E-28	4	Mesenchymal progenitor_4
Rpl13	1.81E-32	0.296291475	1	0.995	2.64E-28	4	Mesenchymal progenitor_4
Gm10260	6.97E-32	0.403176938	0.998	0.981	1.02E-27	4	Mesenchymal progenitor_4
Rplp0	1.01E-31	0.46556105	0.998	0.981	1.47E-27	4	Mesenchymal progenitor_4
Eif2s2	9.41E-31	-0.494382448	0.918	0.956	1.37E-26	4	Mesenchymal progenitor_4
Rps18	1.37E-29	0.355733488	1	0.995	1.99E-25	4	Mesenchymal progenitor_4
Nme2	6.54E-29	0.619236578	0.7	0.386	9.52E-25	4	Mesenchymal progenitor_4
Rps6	2.14E-28	0.332156867	1	0.99	3.12E-24	4	Mesenchymal progenitor_4
Ftl1	1.80E-27	0.370000441	1	0.995	2.62E-23	4	Mesenchymal progenitor_4
Rpl39	4.32E-27	-0.373307591	0.995	0.998	6.29E-23	4	Mesenchymal progenitor_4

Rps7	6.91E-27	0.358162455	0.993	0.976	1.01E-22	4	Mesenchymal progenitor_4
Gm10709	1.02E-26	0.416888995	0.97	0.869	1.48E-22	4	Mesenchymal progenitor_4
Rpl9	2.68E-26	0.306793691	0.998	0.983	3.90E-22	4	Mesenchymal progenitor_4
Ndufa3	3.17E-26	-0.464023214	0.824	0.908	4.62E-22	4	Mesenchymal progenitor_4
Rpl36	8.12E-26	-0.29631695	0.998	0.995	1.18E-21	4	Mesenchymal progenitor_4
Lmo4	2.40E-24	-0.618910824	0.469	0.726	3.50E-20	4	Mesenchymal progenitor_4
Rpl10	3.68E-23	0.35036097	0.99	0.981	5.35E-19	4	Mesenchymal progenitor_4
Rps5	4.07E-22	0.303372781	1	0.995	5.92E-18	4	Mesenchymal progenitor_4
Malat1	4.22E-20	-0.490326999	1	1	6.14E-16	4	Mesenchymal progenitor_4
Psap	5.83E-20	-0.511461036	0.811	0.934	8.49E-16	4	Mesenchymal progenitor_4
Snhg9	8.81E-20	-0.480185197	0.099	0.357	1.28E-15	4	Mesenchymal progenitor_4
Calr	3.10E-19	0.45670719	0.896	0.716	4.51E-15	4	Mesenchymal progenitor_4
Igkc	3.52E-19	1.589007349	0.422	0.146	5.13E-15	4	Mesenchymal progenitor_4
Ppp1r14b	4.63E-19	0.385612066	0.903	0.757	6.74E-15	4	Mesenchymal progenitor_4
Gm10020	1.23E-18	0.451360685	0.566	0.296	1.80E-14	4	Mesenchymal progenitor_4
Grn	2.21E-18	-0.498885008	0.357	0.633	3.21E-14	4	Mesenchymal progenitor_4
mt-Nd3	5.30E-18	-0.50814814	0.211	0.466	7.73E-14	4	Mesenchymal progenitor_4
Dync1i2	1.25E-17	-0.497347303	0.429	0.653	1.82E-13	4	Mesenchymal progenitor_4
Gm10263	1.62E-17	-0.38596568	0.032	0.235	2.36E-13	4	Mesenchymal progenitor_4
Fos	2.19E-17	-0.575061725	0.409	0.653	3.19E-13	4	Mesenchymal progenitor_4
Rpl4	2.49E-17	0.337863045	0.998	0.961	3.63E-13	4	Mesenchymal progenitor_4
Snrpg	4.31E-17	-0.305145017	0.908	0.939	6.28E-13	4	Mesenchymal progenitor_4
Myl9	1.64E-16	-0.431780434	0.908	0.947	2.39E-12	4	Mesenchymal progenitor_4
Rpl17	2.22E-16	0.264160542	0.998	0.971	3.23E-12	4	Mesenchymal progenitor_4

Pabpc1	2.50E-16	0.379409844	0.918	0.84	3.65E-12	4	Mesenchymal progenitor_4
Rpl3	3.68E-16	0.27980456	0.998	0.983	5.35E-12	4	Mesenchymal progenitor_4
Sertad2	3.28E-15	-0.495869739	0.372	0.561	4.77E-11	4	Mesenchymal progenitor_4
Rpl18a	6.07E-15	0.261303068	1	1	8.85E-11	4	Mesenchymal progenitor_4
Ybx1	7.37E-15	0.25788635	0.993	0.964	1.07E-10	4	Mesenchymal progenitor_4
Polr2l	1.41E-14	-0.436367584	0.581	0.716	2.05E-10	4	Mesenchymal progenitor_4
Arpc1b	1.77E-14	0.317341581	0.955	0.835	2.58E-10	4	Mesenchymal progenitor_4
Hes1	3.29E-14	-0.551063896	0.476	0.636	4.80E-10	4	Mesenchymal progenitor_4
Myeov2	5.09E-14	-0.318428889	0.864	0.913	7.42E-10	4	Mesenchymal progenitor_4
Usmg5	6.10E-14	-0.361060802	0.792	0.847	8.89E-10	4	Mesenchymal progenitor_4
Ptrf	7.86E-14	-0.44728575	0.777	0.876	1.14E-09	4	Mesenchymal progenitor_4
2010107E04Rik	1.10E-13	-0.3712519	0.725	0.828	1.60E-09	4	Mesenchymal progenitor_4
Naca	1.94E-13	0.252801272	0.968	0.913	2.83E-09	4	Mesenchymal progenitor_4
Eef1g	4.73E-13	0.26182348	0.98	0.947	6.89E-09	4	Mesenchymal progenitor_4
Eif4ebp1	5.24E-13	0.317323122	0.926	0.784	7.64E-09	4	Mesenchymal progenitor_4
Rpl6l	5.69E-13	0.332359426	0.494	0.255	8.29E-09	4	Mesenchymal progenitor_4
Nisch	6.07E-13	-0.441361198	0.536	0.694	8.84E-09	4	Mesenchymal progenitor_4
Apoe	2.46E-12	-0.373558655	0.132	0.34	3.58E-08	4	Mesenchymal progenitor_4
Crip1	2.57E-12	-0.371062868	0.94	0.951	3.74E-08	4	Mesenchymal progenitor_4
Acta2	2.76E-12	-0.351224305	0.99	0.988	4.01E-08	4	Mesenchymal progenitor_4
Timp1	4.36E-12	0.340968153	0.948	0.867	6.36E-08	4	Mesenchymal progenitor_4
Gm10116	4.57E-12	0.313370646	0.658	0.425	6.66E-08	4	Mesenchymal progenitor_4
Dstn	5.65E-12	-0.331431409	0.893	0.92	8.23E-08	4	Mesenchymal progenitor_4
Atp5g1	6.32E-12	0.271811471	0.938	0.881	9.20E-08	4	Mesenchymal progenitor_4

Ogn	7.97E-12	-0.43208901	0.519	0.677	1.16E-07	4	Mesenchymal progenitor_4
Snhg18	9.49E-12	-0.384768714	0.382	0.566	1.38E-07	4	Mesenchymal progenitor_4
Rps27rt	1.37E-11	-0.372894304	0.273	0.476	2.00E-07	4	Mesenchymal progenitor_4
Usp50	2.40E-11	-0.304289251	0.744	0.828	3.49E-07	4	Mesenchymal progenitor_4
Egr1	5.59E-11	-0.391417158	0.37	0.558	8.15E-07	4	Mesenchymal progenitor_4
Tnfrsf12a	6.92E-11	0.319681612	0.61	0.396	1.01E-06	4	Mesenchymal progenitor_4
Nhp2	7.24E-11	0.344274427	0.72	0.553	1.05E-06	4	Mesenchymal progenitor_4
Pgk1	1.79E-10	0.303054525	0.687	0.461	2.60E-06	4	Mesenchymal progenitor_4
Romo1	1.80E-10	-0.270355513	0.916	0.908	2.63E-06	4	Mesenchymal progenitor_4
Slpi	2.44E-10	0.615037447	0.164	0.032	3.55E-06	4	Mesenchymal progenitor_4
Npm1	2.87E-10	0.315487921	0.943	0.879	4.18E-06	4	Mesenchymal progenitor_4
Mylk	3.87E-10	-0.453219254	0.685	0.811	5.64E-06	4	Mesenchymal progenitor_4
Tomm22	8.25E-10	0.288595514	0.687	0.502	1.20E-05	4	Mesenchymal progenitor_4
Jchain	1.05E-09	0.734320839	0.112	0.01	1.53E-05	4	Mesenchymal progenitor_4
Gm10073	1.38E-09	-0.285257854	0.737	0.806	2.01E-05	4	Mesenchymal progenitor_4
Srm	2.43E-09	0.280111714	0.531	0.323	3.54E-05	4	Mesenchymal progenitor_4
Myh9	2.85E-09	-0.317928219	0.69	0.801	4.15E-05	4	Mesenchymal progenitor_4
Wisp1	4.14E-09	0.358101263	0.734	0.568	6.04E-05	4	Mesenchymal progenitor_4
Ephx1	5.08E-09	-0.3263206	0.171	0.337	7.40E-05	4	Mesenchymal progenitor_4
Gm10036	8.03E-09	0.254124052	0.68	0.471	0.000116884	4	Mesenchymal progenitor_4
Col8a1	1.38E-08	-0.296160954	0.955	0.954	0.000201049	4	Mesenchymal progenitor_4
Timp2	1.41E-08	-0.288180583	0.901	0.903	0.000205778	4	Mesenchymal progenitor_4
Pttg1	2.67E-08	0.333625584	0.715	0.575	0.000389562	4	Mesenchymal progenitor_4
Hspe1	3.46E-08	0.250470908	0.876	0.772	0.000504457	4	Mesenchymal progenitor_4

Gm8186	3.67E-08	-0.327177416	0.471	0.595	0.000534294	4	Mesenchymal progenitor_4
C1qbp	4.49E-08	0.27731955	0.511	0.325	0.000653325	4	Mesenchymal progenitor_4
Tnrc6a	5.54E-08	-0.297350951	0.4	0.541	0.000806493	4	Mesenchymal progenitor_4
Gm9493	6.29E-08	0.276539356	0.452	0.296	0.000915631	4	Mesenchymal progenitor_4
Lasp1	6.35E-08	-0.359026528	0.615	0.699	0.000924792	4	Mesenchymal progenitor_4
Sgol1	6.71E-08	-0.358629832	0.216	0.362	0.000977746	4	Mesenchymal progenitor_4
Ssbp3	7.43E-08	-0.338330824	0.27	0.42	0.001082361	4	Mesenchymal progenitor_4
Gadd45g	8.58E-08	0.361729234	0.67	0.507	0.001249476	4	Mesenchymal progenitor_4
Lockd	1.05E-07	-0.309022465	0.727	0.784	0.001529509	4	Mesenchymal progenitor_4
Aspn	1.13E-07	-0.400919703	0.112	0.248	0.00163848	4	Mesenchymal progenitor_4
Fads3	1.52E-07	0.258796821	0.583	0.427	0.002209133	4	Mesenchymal progenitor_4
Anapc13	1.72E-07	-0.293935745	0.645	0.709	0.00250813	4	Mesenchymal progenitor_4
Tubb4b	2.03E-07	-0.259161896	0.816	0.854	0.002953091	4	Mesenchymal progenitor_4
Cnbp	2.04E-07	0.256645689	0.744	0.626	0.002974289	4	Mesenchymal progenitor_4
Olfml3	2.66E-07	-0.308995856	0.454	0.566	0.003872816	4	Mesenchymal progenitor_4
Larp7	4.43E-07	-0.283066319	0.263	0.408	0.006451052	4	Mesenchymal progenitor_4
Maged1	4.73E-07	-0.256078364	0.608	0.726	0.006886244	4	Mesenchymal progenitor_4
Glrx5	4.77E-07	0.275611051	0.717	0.57	0.006944922	4	Mesenchymal progenitor_4
Rps29	8.84E-99	-0.646832355	1	1	1.29E-94	5	Mesenchymal progenitor_5
Rps28	1.21E-88	-0.858656755	1	1	1.77E-84	5	Mesenchymal progenitor_5
Rpl41	6.38E-80	-0.437151245	1	1	9.29E-76	5	Mesenchymal progenitor_5
Rpl38	1.26E-67	-0.552371601	0.997	1	1.84E-63	5	Mesenchymal progenitor_5
Gm8730	1.40E-61	0.810950839	0.995	0.973	2.04E-57	5	Mesenchymal progenitor_5
Gm26917	1.70E-58	0.748528202	0.967	0.775	2.47E-54	5	Mesenchymal progenitor_5

Rps2	9.75E-52	0.533963177	1	0.991	1.42E-47	5	Mesenchymal progenitor_5
Gm10076	1.26E-46	-0.381182297	1	0.997	1.84E-42	5	Mesenchymal progenitor_5
Rps27	3.73E-45	-0.413041645	0.997	0.997	5.43E-41	5	Mesenchymal progenitor_5
Malat1	3.06E-43	-0.65720076	1	1	4.45E-39	5	Mesenchymal progenitor_5
Rpl36	4.58E-38	-0.366484084	0.997	1	6.66E-34	5	Mesenchymal progenitor_5
Rpl15	5.69E-38	0.576680164	0.977	0.916	8.28E-34	5	Mesenchymal progenitor_5
Snhg9	7.06E-38	-0.715198959	0.077	0.486	1.03E-33	5	Mesenchymal progenitor_5
Rpl37a	1.39E-36	-0.265754772	1	1	2.02E-32	5	Mesenchymal progenitor_5
mt-Nd2	2.11E-36	-0.608683373	0.829	0.952	3.07E-32	5	Mesenchymal progenitor_5
Nme2	1.55E-32	0.682706895	0.714	0.339	2.26E-28	5	Mesenchymal progenitor_5
Nmt1	2.99E-31	-0.67130036	0.276	0.661	4.35E-27	5	Mesenchymal progenitor_5
Atp5k	7.66E-31	-0.510167375	0.906	0.973	1.12E-26	5	Mesenchymal progenitor_5
Calr	3.10E-25	0.538847342	0.923	0.733	4.52E-21	5	Mesenchymal progenitor_5
Gm10260	1.32E-24	0.35037907	0.997	0.991	1.92E-20	5	Mesenchymal progenitor_5
Rplp0	1.65E-24	0.39929282	0.992	0.994	2.40E-20	5	Mesenchymal progenitor_5
Rpl10	1.62E-23	0.356724133	0.992	0.973	2.35E-19	5	Mesenchymal progenitor_5
Rpl29	2.44E-23	0.501952847	0.867	0.703	3.55E-19	5	Mesenchymal progenitor_5
Eif2s2	3.82E-23	-0.431748357	0.898	0.976	5.56E-19	5	Mesenchymal progenitor_5
Sertad2	4.98E-23	-0.575769545	0.334	0.631	7.25E-19	5	Mesenchymal progenitor_5
Rps18	5.78E-23	0.307142121	1	0.997	8.42E-19	5	Mesenchymal progenitor_5
Rpl39	2.62E-21	-0.348353827	1	0.991	3.82E-17	5	Mesenchymal progenitor_5
Ibsp	2.63E-21	0.732177205	0.821	0.477	3.83E-17	5	Mesenchymal progenitor_5
Rps21	1.67E-20	-0.324838921	0.985	0.994	2.43E-16	5	Mesenchymal progenitor_5
Ndufa3	4.04E-20	-0.447426639	0.839	0.913	5.88E-16	5	Mesenchymal progenitor_5

Polr2l	6.66E-19	-0.480796873	0.566	0.79	9.69E-15	5	Mesenchymal progenitor_5
Sec61g	2.70E-17	-0.26002692	0.995	0.988	3.94E-13	5	Mesenchymal progenitor_5
Lmo4	2.90E-17	-0.503677476	0.482	0.733	4.22E-13	5	Mesenchymal progenitor_5
Rpl9	3.85E-17	0.25746369	1	0.988	5.61E-13	5	Mesenchymal progenitor_5
Rps7	3.64E-16	0.289001422	0.992	0.988	5.31E-12	5	Mesenchymal progenitor_5
Ftl1	6.94E-16	0.287200959	1	1	1.01E-11	5	Mesenchymal progenitor_5
Rps6	1.23E-15	0.266862677	0.995	0.994	1.80E-11	5	Mesenchymal progenitor_5
Rps5	1.26E-15	0.268025936	1	1	1.83E-11	5	Mesenchymal progenitor_5
Timp1	2.47E-15	0.467902917	0.952	0.841	3.60E-11	5	Mesenchymal progenitor_5
Psap	3.14E-15	-0.399143588	0.867	0.931	4.57E-11	5	Mesenchymal progenitor_5
Igkc	3.86E-15	0.387513714	0.411	0.15	5.62E-11	5	Mesenchymal progenitor_5
Hes1	1.24E-14	-0.570241082	0.467	0.67	1.81E-10	5	Mesenchymal progenitor_5
Gm10020	2.34E-14	0.398137225	0.577	0.315	3.41E-10	5	Mesenchymal progenitor_5
Eif4ebp1	3.95E-14	0.339277525	0.934	0.826	5.75E-10	5	Mesenchymal progenitor_5
2010107E04Rik	3.96E-14	-0.367805878	0.679	0.82	5.77E-10	5	Mesenchymal progenitor_5
Actn4	4.20E-14	-0.361915092	0.819	0.898	6.12E-10	5	Mesenchymal progenitor_5
Gm10263	4.77E-14	-0.296225802	0.041	0.231	6.95E-10	5	Mesenchymal progenitor_5
Ybx1	2.27E-13	0.252604038	0.992	0.979	3.31E-09	5	Mesenchymal progenitor_5
Dstn	6.33E-13	-0.367158204	0.908	0.943	9.22E-09	5	Mesenchymal progenitor_5
Myl9	6.67E-13	-0.381859745	0.906	0.958	9.71E-09	5	Mesenchymal progenitor_5
Fos	9.33E-13	-0.5726957	0.523	0.724	1.36E-08	5	Mesenchymal progenitor_5
mt-Nd3	1.23E-12	-0.418472946	0.281	0.52	1.80E-08	5	Mesenchymal progenitor_5
Usmg5	3.09E-12	-0.336772675	0.793	0.88	4.50E-08	5	Mesenchymal progenitor_5
Usp50	3.33E-12	-0.324593791	0.724	0.847	4.85E-08	5	Mesenchymal progenitor_5

Nupr1	4.04E-12	0.398756551	0.934	0.832	5.88E-08	5	Mesenchymal progenitor_5
Grn	4.83E-12	-0.400123271	0.378	0.592	7.04E-08	5	Mesenchymal progenitor_5
Rpl3	1.38E-11	0.259744021	0.992	0.982	2.02E-07	5	Mesenchymal progenitor_5
Myeov2	1.83E-11	-0.315577339	0.88	0.916	2.66E-07	5	Mesenchymal progenitor_5
Pet100	2.40E-11	-0.358363853	0.306	0.529	3.49E-07	5	Mesenchymal progenitor_5
Erh	3.27E-11	0.302111822	0.923	0.841	4.76E-07	5	Mesenchymal progenitor_5
Gm8186	4.29E-11	-0.356694773	0.426	0.61	6.25E-07	5	Mesenchymal progenitor_5
Dync1i2	5.10E-11	-0.34197651	0.36	0.577	7.43E-07	5	Mesenchymal progenitor_5
Dmpk	5.98E-11	-0.308436214	0.097	0.279	8.70E-07	5	Mesenchymal progenitor_5
Ppp1r14b	2.32E-10	0.299076393	0.872	0.751	3.38E-06	5	Mesenchymal progenitor_5
Pgk1	3.98E-10	0.354775185	0.625	0.429	5.80E-06	5	Mesenchymal progenitor_5
Romo1	4.57E-10	-0.25398581	0.901	0.964	6.65E-06	5	Mesenchymal progenitor_5
Npm1	7.21E-10	0.261376785	0.969	0.94	1.05E-05	5	Mesenchymal progenitor_5
Ptrf	8.41E-10	-0.316859364	0.74	0.883	1.22E-05	5	Mesenchymal progenitor_5
Crip1	1.03E-09	-0.328030963	0.936	0.964	1.50E-05	5	Mesenchymal progenitor_5
Hk2	1.06E-09	-0.312492037	0.115	0.285	1.55E-05	5	Mesenchymal progenitor_5
Gm10073	1.70E-09	-0.291135977	0.758	0.841	2.48E-05	5	Mesenchymal progenitor_5
Pdia3	1.80E-09	0.316887825	0.477	0.285	2.62E-05	5	Mesenchymal progenitor_5
Tomm7	2.72E-09	-0.276012903	0.765	0.838	3.97E-05	5	Mesenchymal progenitor_5
Pam	3.58E-09	-0.324681049	0.372	0.577	5.21E-05	5	Mesenchymal progenitor_5
Nisch	5.95E-09	-0.317610359	0.515	0.688	8.67E-05	5	Mesenchymal progenitor_5
Snhg18	1.09E-08	-0.327016682	0.505	0.64	0.000158835	5	Mesenchymal progenitor_5
Gm10116	1.32E-08	0.303292775	0.592	0.399	0.000192552	5	Mesenchymal progenitor_5
Rps27rt	2.13E-08	-0.342287795	0.334	0.495	0.000310778	5	Mesenchymal progenitor_5

Timm13	4.82E-08	0.25396212	0.913	0.799	0.000702031	5	Mesenchymal progenitor_5
Ogn	5.34E-08	-0.393970224	0.543	0.685	0.000777745	5	Mesenchymal progenitor_5
Sap18	6.70E-08	0.269274377	0.722	0.526	0.000976375	5	Mesenchymal progenitor_5
Flna	7.01E-08	-0.2535229	0.918	0.964	0.001021037	5	Mesenchymal progenitor_5
Eif3k	8.09E-08	0.260680596	0.865	0.775	0.001178656	5	Mesenchymal progenitor_5
Timm17a	1.71E-07	0.261173173	0.663	0.462	0.002491355	5	Mesenchymal progenitor_5
Toporsos	1.77E-07	-0.250205811	0.207	0.372	0.002580634	5	Mesenchymal progenitor_5
Egr1	1.97E-07	-0.337858747	0.393	0.565	0.002873086	5	Mesenchymal progenitor_5
Mylk	3.61E-07	-0.322799336	0.77	0.835	0.005252034	5	Mesenchymal progenitor_5
Rps29	4.16E-76	-0.633166105	0.997	1	6.05E-72	6	Mesenchymal progenitor_6
Rps28	8.45E-63	-0.773700262	0.987	0.993	1.23E-58	6	Mesenchymal progenitor_6
Gm8730	1.31E-60	0.827986638	0.997	0.93	1.91E-56	6	Mesenchymal progenitor_6
Rps2	9.31E-55	0.663624222	0.997	0.974	1.36E-50	6	Mesenchymal progenitor_6
Rpl38	4.17E-48	-0.557334533	1	0.996	6.07E-44	6	Mesenchymal progenitor_6
Gm26917	4.87E-40	0.798787992	0.917	0.533	7.09E-36	6	Mesenchymal progenitor_6
Rps27	1.18E-39	-0.425111646	0.997	1	1.72E-35	6	Mesenchymal progenitor_6
Rpl15	2.01E-35	0.584193835	0.987	0.837	2.93E-31	6	Mesenchymal progenitor_6
Rplp0	2.52E-34	0.480708978	0.997	0.967	3.66E-30	6	Mesenchymal progenitor_6
Rpl41	1.04E-33	-0.353522029	1	1	1.51E-29	6	Mesenchymal progenitor_6
Rps18	1.58E-32	0.434598842	1	0.981	2.31E-28	6	Mesenchymal progenitor_6
Rpl13	8.00E-32	0.316557737	1	1	1.17E-27	6	Mesenchymal progenitor_6
Gm10260	1.31E-30	0.432410995	0.997	0.948	1.91E-26	6	Mesenchymal progenitor_6
Nme2	2.43E-29	0.787756897	0.646	0.204	3.54E-25	6	Mesenchymal progenitor_6
Atp5k	9.82E-28	-0.634475817	0.815	0.922	1.43E-23	6	Mesenchymal progenitor_6

Rps6	7.50E-27	0.39022907	1	0.967	1.09E-22	6	Mesenchymal progenitor_6
Calr	2.36E-25	0.681482369	0.859	0.537	3.43E-21	6	Mesenchymal progenitor_6
Ibsp	2.85E-24	0.689144786	0.714	0.256	4.15E-20	6	Mesenchymal progenitor_6
Rpl10	1.05E-23	0.381064417	0.992	0.959	1.54E-19	6	Mesenchymal progenitor_6
Rps5	1.79E-23	0.349369061	1	0.981	2.61E-19	6	Mesenchymal progenitor_6
Rpl9	5.41E-22	0.364093096	0.995	0.952	7.88E-18	6	Mesenchymal progenitor_6
Eef1a1	1.33E-20	0.317833788	1	0.985	1.94E-16	6	Mesenchymal progenitor_6
Rpl32	5.11E-20	0.283928729	1	0.993	7.44E-16	6	Mesenchymal progenitor_6
Nmt1	8.99E-20	-0.699383386	0.208	0.511	1.31E-15	6	Mesenchymal progenitor_6
Rpl19	2.07E-19	0.296485554	0.997	0.981	3.01E-15	6	Mesenchymal progenitor_6
Fos	8.84E-19	-1.192164007	0.31	0.574	1.29E-14	6	Mesenchymal progenitor_6
Timp1	1.11E-18	0.566755976	0.94	0.73	1.62E-14	6	Mesenchymal progenitor_6
Rpl13a	4.08E-18	0.257862416	1	0.996	5.94E-14	6	Mesenchymal progenitor_6
Eif4ebp1	4.28E-18	0.52495	0.878	0.581	6.24E-14	6	Mesenchymal progenitor_6
Rpl29	4.79E-18	0.528953852	0.729	0.393	6.97E-14	6	Mesenchymal progenitor_6
Malat1	7.28E-18	-0.572042781	1	1	1.06E-13	6	Mesenchymal progenitor_6
Gm10076	1.07E-17	-0.309725237	1	0.996	1.56E-13	6	Mesenchymal progenitor_6
Eif1	2.12E-17	0.324218279	0.995	0.959	3.08E-13	6	Mesenchymal progenitor_6
mt-Nd2	5.92E-17	-0.638821913	0.565	0.752	8.63E-13	6	Mesenchymal progenitor_6
Rpl4	7.10E-17	0.356663933	0.987	0.944	1.03E-12	6	Mesenchymal progenitor_6
Rpl23a	7.86E-17	0.304251273	0.99	0.967	1.15E-12	6	Mesenchymal progenitor_6
Rps3	4.81E-16	0.271124881	1	0.985	7.01E-12	6	Mesenchymal progenitor_6
Rpl3	1.38E-15	0.344195689	0.99	0.959	2.00E-11	6	Mesenchymal progenitor_6
Snhg9	5.95E-15	-0.609968947	0.06	0.27	8.66E-11	6	Mesenchymal progenitor_6

Rps7	5.97E-15	0.305216548	0.984	0.948	8.70E-11	6	Mesenchymal progenitor_6
Rps4x	6.35E-15	0.279515134	0.997	0.963	9.24E-11	6	Mesenchymal progenitor_6
Manf	6.48E-15	0.53052328	0.716	0.393	9.44E-11	6	Mesenchymal progenitor_6
Ndufa3	9.48E-15	-0.474459853	0.76	0.83	1.38E-10	6	Mesenchymal progenitor_6
Gm10709	1.34E-14	0.361990228	0.927	0.807	1.94E-10	6	Mesenchymal progenitor_6
Col8a1	2.91E-14	-0.462315706	0.938	0.97	4.23E-10	6	Mesenchymal progenitor_6
Rpl14	9.34E-14	0.270771563	0.997	0.959	1.36E-09	6	Mesenchymal progenitor_6
Rps3a1	4.17E-13	0.260071743	1	0.993	6.07E-09	6	Mesenchymal progenitor_6
Rpl7	6.47E-13	0.316503855	0.982	0.893	9.42E-09	6	Mesenchymal progenitor_6
Eif2s2	2.94E-12	-0.431555828	0.867	0.881	4.28E-08	6	Mesenchymal progenitor_6
Lox	3.69E-12	0.517386439	0.823	0.611	5.38E-08	6	Mesenchymal progenitor_6
Rpl36	5.08E-12	-0.27023402	0.992	0.993	7.40E-08	6	Mesenchymal progenitor_6
Cyb5r1	6.21E-12	0.448280492	0.401	0.141	9.05E-08	6	Mesenchymal progenitor_6
Gstp1	8.18E-12	0.468002303	0.656	0.378	1.19E-07	6	Mesenchymal progenitor_6
Ppp1r14b	9.16E-12	0.440300639	0.732	0.474	1.33E-07	6	Mesenchymal progenitor_6
Col6a2	1.50E-11	-0.39332995	0.919	0.922	2.19E-07	6	Mesenchymal progenitor_6
Eno1	2.02E-11	0.433536811	0.857	0.689	2.95E-07	6	Mesenchymal progenitor_6
Sec61g	2.26E-11	-0.274970737	0.969	0.959	3.30E-07	6	Mesenchymal progenitor_6
Polr2l	2.70E-11	-0.583236817	0.406	0.581	3.94E-07	6	Mesenchymal progenitor_6
Arpc1b	5.23E-11	0.320623556	0.94	0.807	7.62E-07	6	Mesenchymal progenitor_6
Hspa5	5.29E-11	0.475487265	0.732	0.463	7.71E-07	6	Mesenchymal progenitor_6
Gm10020	8.92E-11	0.312254397	0.451	0.181	1.30E-06	6	Mesenchymal progenitor_6
Sertad2	1.42E-10	-0.56986032	0.297	0.485	2.07E-06	6	Mesenchymal progenitor_6
Igkc	3.69E-10	-1.31796731	0.286	0.078	5.37E-06	6	Mesenchymal progenitor_6

Hnrnpa3	6.06E-10	0.341763903	0.81	0.533	8.82E-06	6	Mesenchymal progenitor_6
Rbm3	7.30E-10	0.288242413	0.974	0.856	1.06E-05	6	Mesenchymal progenitor_6
Rps21	1.10E-09	-0.256063461	0.961	0.959	1.60E-05	6	Mesenchymal progenitor_6
Col6a1	1.13E-09	-0.351875723	0.935	0.956	1.64E-05	6	Mesenchymal progenitor_6
Usmg5	1.57E-09	-0.412256711	0.706	0.774	2.29E-05	6	Mesenchymal progenitor_6
Lsm4	2.01E-09	0.397869973	0.576	0.307	2.93E-05	6	Mesenchymal progenitor_6
Egr1	2.12E-09	-0.783047822	0.362	0.522	3.08E-05	6	Mesenchymal progenitor_6
Arl6ip5	2.61E-09	0.398141365	0.391	0.167	3.81E-05	6	Mesenchymal progenitor_6
Tomm7	2.79E-09	-0.362312733	0.664	0.767	4.06E-05	6	Mesenchymal progenitor_6
Vim	3.42E-09	0.294412881	0.984	0.93	4.98E-05	6	Mesenchymal progenitor_6
Pabpc1	4.46E-09	0.319157607	0.859	0.674	6.49E-05	6	Mesenchymal progenitor_6
Pdgfrb	4.48E-09	-0.455364845	0.81	0.867	6.52E-05	6	Mesenchymal progenitor_6
Rpl27	4.74E-09	0.270747794	0.938	0.844	6.91E-05	6	Mesenchymal progenitor_6
Hspa9	6.64E-09	0.312321171	0.753	0.485	9.67E-05	6	Mesenchymal progenitor_6
Fads3	9.97E-09	0.362844804	0.503	0.267	0.00014517	6	Mesenchymal progenitor_6
Gm6576	1.28E-08	0.260434777	0.138	0.011	0.000185932	6	Mesenchymal progenitor_6
Mgp	1.62E-08	-0.447824329	0.865	0.926	0.000235316	6	Mesenchymal progenitor_6
Gm10126	1.79E-08	-0.302978055	0.018	0.126	0.000260529	6	Mesenchymal progenitor_6
Ybx1	1.81E-08	0.267983242	0.961	0.881	0.00026423	6	Mesenchymal progenitor_6
Psat1	1.87E-08	0.318094164	0.297	0.104	0.00027166	6	Mesenchymal progenitor_6
mt-Nd3	1.90E-08	-0.440604862	0.104	0.259	0.000275991	6	Mesenchymal progenitor_6
Ftl1	1.96E-08	0.259682453	1	0.996	0.000285969	6	Mesenchymal progenitor_6
Txn1	1.97E-08	0.330047545	0.878	0.656	0.000286895	6	Mesenchymal progenitor_6
Rpl6l	2.02E-08	0.28398969	0.422	0.185	0.000293558	6	Mesenchymal progenitor_6

Ptrf	2.57E-08	-0.434117966	0.62	0.7	0.00037377	6	Mesenchymal progenitor_6
Adrm1	2.57E-08	0.31204843	0.464	0.23	0.000374315	6	Mesenchymal progenitor_6
Rnf10	3.51E-08	0.290083955	0.331	0.13	0.000511884	6	Mesenchymal progenitor_6
Ctsb	3.52E-08	-0.683668774	0.51	0.615	0.00051271	6	Mesenchymal progenitor_6
Btf3	4.06E-08	0.277017213	0.917	0.722	0.000591089	6	Mesenchymal progenitor_6
Pgk1	4.73E-08	0.322163116	0.469	0.237	0.000688214	6	Mesenchymal progenitor_6
Nudt8	4.77E-08	0.255626967	0.195	0.044	0.000695235	6	Mesenchymal progenitor_6
Hmga1-rs1	5.94E-08	0.270449867	0.164	0.03	0.000865021	6	Mesenchymal progenitor_6
Gm9493	7.07E-08	0.25553194	0.328	0.126	0.001028991	6	Mesenchymal progenitor_6
Eef1b2	7.74E-08	0.28643333	0.87	0.644	0.001126656	6	Mesenchymal progenitor_6
Ldha	7.74E-08	0.293194424	0.919	0.811	0.001127181	6	Mesenchymal progenitor_6
Fosb	8.54E-08	-0.610766587	0.107	0.252	0.001244186	6	Mesenchymal progenitor_6
Yif1b	1.05E-07	0.262653193	0.424	0.2	0.001526541	6	Mesenchymal progenitor_6
Nhp2	1.13E-07	0.30093426	0.594	0.352	0.001645696	6	Mesenchymal progenitor_6
Hspe1	1.16E-07	0.346633662	0.82	0.633	0.001684175	6	Mesenchymal progenitor_6
Slc25a5	1.50E-07	0.287512295	0.846	0.644	0.002184111	6	Mesenchymal progenitor_6
Eif3k	1.94E-07	0.281236091	0.833	0.652	0.002822833	6	Mesenchymal progenitor_6
Vamp5	2.17E-07	0.305679969	0.461	0.237	0.003154531	6	Mesenchymal progenitor_6
Timm17a	2.22E-07	0.284675884	0.568	0.326	0.003240115	6	Mesenchymal progenitor_6
Gm10116	2.32E-07	0.335569106	0.51	0.296	0.003379436	6	Mesenchymal progenitor_6
Lrp1	2.92E-07	-0.368224792	0.562	0.656	0.004259825	6	Mesenchymal progenitor_6
Romo1	3.23E-07	-0.296730775	0.87	0.881	0.004710029	6	Mesenchymal progenitor_6
Rtn4	3.34E-07	0.340252777	0.753	0.567	0.004869188	6	Mesenchymal progenitor_6
Gm10036	3.66E-07	0.322503834	0.549	0.337	0.005325305	6	Mesenchymal progenitor_6

Pdia3	3.74E-07	0.320845697	0.414	0.211	0.005440252	6	Mesenchymal progenitor_6
Psap	4.10E-07	-0.334606096	0.758	0.785	0.005964936	6	Mesenchymal progenitor_6
Eif3f	4.66E-07	0.252472966	0.88	0.748	0.006793453	6	Mesenchymal progenitor_6
Srsf10	4.79E-07	0.299171904	0.323	0.141	0.006972267	6	Mesenchymal progenitor_6
Mthfd2	6.11E-07	0.274737766	0.396	0.189	0.008900385	6	Mesenchymal progenitor_6
Arpc1a	6.50E-07	0.27806505	0.443	0.233	0.009462679	6	Mesenchymal progenitor_6
Snhg18	6.71E-07	-0.501696109	0.427	0.533	0.009779364	6	Mesenchymal progenitor_6
Rps28	7.24E-51	-0.97863109	0.996	1	1.05E-46	7	Endothelial progenitor_7
Rps29	4.75E-49	-0.667939893	1	1	6.92E-45	7	Endothelial progenitor_7
Rpl38	3.61E-42	-0.592961626	1	1	5.26E-38	7	Endothelial progenitor_7
Rpl41	4.22E-42	-0.552787497	1	1	6.15E-38	7	Endothelial progenitor_7
Gm8730	7.82E-40	0.809722709	0.991	0.96	1.14E-35	7	Endothelial progenitor_7
Rps27	9.29E-36	-0.472030592	1	1	1.35E-31	7	Endothelial progenitor_7
Gm26917	3.42E-33	0.745774245	0.953	0.718	4.98E-29	7	Endothelial progenitor_7
Rpl37a	2.34E-29	-0.392123812	1	1	3.41E-25	7	Endothelial progenitor_7
Rps21	9.34E-29	-0.548912408	0.996	0.994	1.36E-24	7	Endothelial progenitor_7
Rpl15	1.56E-28	0.567086734	0.978	0.927	2.28E-24	7	Endothelial progenitor_7
Gm10076	2.82E-27	-0.465142844	0.996	0.994	4.11E-23	7	Endothelial progenitor_7
Rplp0	2.26E-26	0.52342237	1	0.977	3.30E-22	7	Endothelial progenitor_7
Eif2s2	4.80E-25	-0.569429442	0.901	0.972	6.99E-21	7	Endothelial progenitor_7
Ptrf	6.21E-25	-0.807099758	0.841	0.955	9.05E-21	7	Endothelial progenitor_7
Rps2	2.84E-23	0.475431188	1	0.994	4.14E-19	7	Endothelial progenitor_7
Snhg9	1.17E-22	-0.858969229	0.099	0.514	1.71E-18	7	Endothelial progenitor_7
Atp5k	1.91E-22	-0.605728638	0.853	0.949	2.78E-18	7	Endothelial progenitor_7

Nmt1	2.07E-22	-0.803305534	0.267	0.672	3.01E-18	7	Endothelial progenitor_7
mt-Nd2	4.49E-21	-0.7243953	0.763	0.915	6.54E-17	7	Endothelial progenitor_7
Col1a2	2.71E-19	0.443946384	0.978	0.757	3.95E-15	7	Endothelial progenitor_7
Cd200	5.45E-19	-0.940522177	0.409	0.763	7.94E-15	7	Endothelial progenitor_7
Bst2	6.42E-19	0.668331974	0.681	0.237	9.34E-15	7	Endothelial progenitor_7
Ibsp	4.19E-18	0.675723426	0.672	0.237	6.10E-14	7	Endothelial progenitor_7
Rpl36	4.42E-17	-0.358751932	0.996	0.994	6.44E-13	7	Endothelial progenitor_7
Rpl10	2.23E-16	0.381394178	0.991	0.96	3.25E-12	7	Endothelial progenitor_7
Hes1	2.49E-16	-0.868321541	0.422	0.723	3.62E-12	7	Endothelial progenitor_7
Malat1	9.55E-16	-0.493822802	1	0.994	1.39E-11	7	Endothelial progenitor_7
Marcks	6.15E-14	-0.548488538	0.888	0.96	8.96E-10	7	Endothelial progenitor_7
Eno1	6.47E-14	0.511802736	0.94	0.791	9.42E-10	7	Endothelial progenitor_7
Rpl29	7.13E-14	0.503476115	0.841	0.582	1.04E-09	7	Endothelial progenitor_7
Sep15	8.49E-14	0.426830487	0.931	0.78	1.24E-09	7	Endothelial progenitor_7
Polr2l	2.51E-13	-0.565983795	0.5	0.751	3.66E-09	7	Endothelial progenitor_7
Rpl39	2.57E-13	-0.40100621	0.974	0.983	3.75E-09	7	Endothelial progenitor_7
Rpl4	2.93E-13	0.368712666	0.983	0.983	4.26E-09	7	Endothelial progenitor_7
Ftl1	3.33E-13	0.292995893	1	1	4.85E-09	7	Endothelial progenitor_7
Rpl3	1.23E-12	0.379341981	0.978	0.944	1.79E-08	7	Endothelial progenitor_7
Ctla2a	4.94E-12	0.614421223	1	0.972	7.20E-08	7	Endothelial progenitor_7
Rpl18a	6.14E-12	0.261360081	1	1	8.94E-08	7	Endothelial progenitor_7
Rps3	6.36E-12	0.253086576	1	1	9.27E-08	7	Endothelial progenitor_7
Rpl37	6.65E-12	-0.267199559	0.996	0.994	9.69E-08	7	Endothelial progenitor_7
Arpc1b	7.55E-12	0.362481602	0.983	0.91	1.10E-07	7	Endothelial progenitor_7

Tfpi2	7.74E-12	0.829045555	0.608	0.294	1.13E-07	7	Endothelial progenitor_7
Slc25a4	1.02E-11	0.405893622	0.961	0.836	1.49E-07	7	Endothelial progenitor_7
Rps7	1.56E-11	0.3046903	0.987	0.955	2.28E-07	7	Endothelial progenitor_7
Cdh5	2.81E-11	-0.371574176	1	0.994	4.09E-07	7	Endothelial progenitor_7
Gm10126	3.77E-11	-0.302410639	0.009	0.198	5.49E-07	7	Endothelial progenitor_7
Sertad2	3.80E-11	-0.593216842	0.315	0.571	5.54E-07	7	Endothelial progenitor_7
Gm10020	4.12E-11	0.443220452	0.651	0.333	5.99E-07	7	Endothelial progenitor_7
Rps18	4.43E-11	0.296055505	0.991	0.983	6.45E-07	7	Endothelial progenitor_7
Pcdh17	6.44E-11	-0.699298971	0.664	0.808	9.38E-07	7	Endothelial progenitor_7
Rps6	1.53E-10	0.268485337	1	0.994	2.23E-06	7	Endothelial progenitor_7
Dync1i2	2.42E-10	-0.445660231	0.552	0.757	3.53E-06	7	Endothelial progenitor_7
Jag1	3.21E-10	0.671702364	0.728	0.463	4.68E-06	7	Endothelial progenitor_7
Gpihbp1	3.94E-10	-0.518857661	0.789	0.944	5.74E-06	7	Endothelial progenitor_7
Gng11	4.20E-10	0.309748413	0.996	0.989	6.11E-06	7	Endothelial progenitor_7
Tomm7	5.99E-10	-0.377870047	0.875	0.932	8.73E-06	7	Endothelial progenitor_7
Gm10709	1.44E-09	0.344003052	0.948	0.847	2.09E-05	7	Endothelial progenitor_7
Ndufa3	2.14E-09	-0.459119589	0.78	0.842	3.12E-05	7	Endothelial progenitor_7
Pgk1	2.22E-09	0.430199044	0.711	0.418	3.24E-05	7	Endothelial progenitor_7
Psap	2.82E-09	-0.426551214	0.897	0.938	4.10E-05	7	Endothelial progenitor_7
Myh9	4.06E-09	-0.456089102	0.655	0.797	5.91E-05	7	Endothelial progenitor_7
Timm17a	4.30E-09	0.398101529	0.733	0.497	6.26E-05	7	Endothelial progenitor_7
Rps27rt	4.34E-09	-0.440849986	0.25	0.508	6.32E-05	7	Endothelial progenitor_7
Gm10260	4.54E-09	0.272141874	0.987	0.966	6.61E-05	7	Endothelial progenitor_7
Nisch	5.53E-09	-0.429517754	0.672	0.825	8.05E-05	7	Endothelial progenitor_7

Cdkn1a	7.89E-09	0.350960372	0.909	0.768	0.000114932	7	Endothelial progenitor_7
Sec61g	8.41E-09	-0.276963996	0.961	0.977	0.000122541	7	Endothelial progenitor_7
Igkc	1.25E-08	0.34145048	0.401	0.141	0.000182287	7	Endothelial progenitor_7
Bola2	1.68E-08	-0.315407554	0.94	0.96	0.000244139	7	Endothelial progenitor_7
Pet100	2.21E-08	-0.444609887	0.409	0.633	0.000321704	7	Endothelial progenitor_7
Ppp1r14b	2.23E-08	0.320179912	0.948	0.876	0.000324182	7	Endothelial progenitor_7
Col1a1	2.55E-08	0.260192863	0.94	0.78	0.000370888	7	Endothelial progenitor_7
Gm10073	2.99E-08	-0.403318785	0.612	0.763	0.000435895	7	Endothelial progenitor_7
Prss23	3.27E-08	0.391608967	0.879	0.734	0.000476657	7	Endothelial progenitor_7
Eif4ebp1	3.86E-08	0.33905386	0.927	0.774	0.000562168	7	Endothelial progenitor_7
mt-Nd3	4.29E-08	-0.436684428	0.155	0.373	0.000625352	7	Endothelial progenitor_7
Ndufs3	4.31E-08	0.315138739	0.599	0.288	0.000627608	7	Endothelial progenitor_7
Lsm4	4.57E-08	0.330989913	0.759	0.531	0.000665822	7	Endothelial progenitor_7
Slc25a5	4.66E-08	0.388942593	0.858	0.689	0.000678804	7	Endothelial progenitor_7
Plau	6.37E-08	-0.446279992	0.065	0.249	0.000928133	7	Endothelial progenitor_7
Morc4	6.59E-08	-0.312529503	0.082	0.282	0.000959816	7	Endothelial progenitor_7
Manf	6.98E-08	0.308818179	0.849	0.633	0.001016873	7	Endothelial progenitor_7
Hspg2	7.17E-08	-0.332443464	0.948	0.966	0.001043823	7	Endothelial progenitor_7
Fabp5	8.03E-08	-0.495796193	0.362	0.576	0.001170181	7	Endothelial progenitor_7
Pdgfa	9.13E-08	0.440816205	0.595	0.339	0.001329688	7	Endothelial progenitor_7
Atp6v0e	9.15E-08	0.298481244	0.953	0.836	0.00133317	7	Endothelial progenitor_7
Rtn4	9.64E-08	0.286319288	0.953	0.87	0.00140417	7	Endothelial progenitor_7
Tgfbr2	1.02E-07	-0.465566465	0.608	0.734	0.001488461	7	Endothelial progenitor_7
Gnas	1.13E-07	0.264358802	0.966	0.904	0.00164866	7	Endothelial progenitor_7

B2m	1.29E-07	0.260591781	1	0.927	0.001872909	7	Endothelial progenitor_7
Gm10263	1.46E-07	-0.390550967	0.073	0.26	0.002123856	7	Endothelial progenitor_7
Myeov2	1.47E-07	-0.332282935	0.858	0.932	0.002139634	7	Endothelial progenitor_7
Mgll	1.77E-07	-0.505404157	0.121	0.316	0.002571719	7	Endothelial progenitor_7
D8Ertd738e	1.84E-07	0.313333304	0.918	0.723	0.002676069	7	Endothelial progenitor_7
Pdia3	1.98E-07	0.368791434	0.56	0.311	0.002877495	7	Endothelial progenitor_7
Ccdc85b	2.02E-07	0.290576527	0.944	0.791	0.002938649	7	Endothelial progenitor_7
Srm	2.15E-07	0.348015544	0.543	0.311	0.003135817	7	Endothelial progenitor_7
Eef1g	2.59E-07	0.252612547	0.978	0.927	0.003765999	7	Endothelial progenitor_7
Kcne3	2.72E-07	0.48033313	0.875	0.774	0.003968165	7	Endothelial progenitor_7
Clic1	2.78E-07	0.277072365	0.953	0.881	0.004043372	7	Endothelial progenitor_7
Rbp7	2.86E-07	-0.469332036	0.03	0.175	0.0041723	7	Endothelial progenitor_7
Nupr1	3.45E-07	0.378092804	0.647	0.379	0.005026946	7	Endothelial progenitor_7
Igfbp7	4.57E-07	0.351585048	0.953	0.944	0.00665942	7	Endothelial progenitor_7
Ly6e	4.66E-07	-0.433677259	0.78	0.876	0.006790312	7	Endothelial progenitor_7
Abca3	4.94E-07	-0.354372064	0.078	0.254	0.007195849	7	Endothelial progenitor_7
Bax	5.11E-07	0.251764981	0.935	0.893	0.007441193	7	Endothelial progenitor_7
Gm9493	5.40E-07	0.288654483	0.435	0.192	0.007871038	7	Endothelial progenitor_7
Actn4	5.61E-07	-0.320250263	0.853	0.881	0.008177565	7	Endothelial progenitor_7
Rapgef4	6.16E-07	-0.374431312	0.185	0.39	0.008970702	7	Endothelial progenitor_7
Rps28	3.67E-22	-0.985310532	1	1	5.35E-18	8	Osteoblast_8
Snhg9	8.36E-21	-0.695407422	0.093	0.65	1.22E-16	8	Osteoblast_8
Rps29	9.43E-21	-0.70357847	1	1	1.37E-16	8	Osteoblast_8
Rpl38	2.47E-18	-0.742981501	1	1	3.60E-14	8	Osteoblast_8

Gm8730	6.18E-18	0.813088399	1	1	9.00E-14	8	Osteoblast_8
Rpl41	1.01E-17	-0.466405446	1	1	1.48E-13	8	Osteoblast_8
Nme2	1.15E-16	1.022484266	0.955	0.525	1.68E-12	8	Osteoblast_8
Smpd3	2.17E-16	-1.580840638	0.762	0.925	3.17E-12	8	Osteoblast_8
Gm26917	1.51E-15	0.799178195	0.974	0.8	2.20E-11	8	Osteoblast_8
Rpl15	5.73E-15	0.647034312	0.996	0.9	8.34E-11	8	Osteoblast_8
Rps27	2.29E-14	-0.432489171	1	1	3.33E-10	8	Osteoblast_8
Lmo4	2.97E-13	-0.972389838	0.773	0.975	4.32E-09	8	Osteoblast_8
Rps2	3.37E-13	0.447322188	1	1	4.91E-09	8	Osteoblast_8
Gm10076	6.15E-13	-0.397035245	1	1	8.96E-09	8	Osteoblast_8
mt-Nd2	1.74E-12	-1.042700003	0.781	0.925	2.53E-08	8	Osteoblast_8
Gm10263	8.03E-12	-0.610274152	0.071	0.425	1.17E-07	8	Osteoblast_8
Rpl36	1.34E-11	-0.407791854	1	1	1.95E-07	8	Osteoblast_8
Eif2s2	2.25E-11	-0.575872329	0.967	1	3.28E-07	8	Osteoblast_8
Ftl1	2.37E-11	0.546163916	1	1	3.45E-07	8	Osteoblast_8
Rpl13	1.78E-10	0.280426818	1	1	2.59E-06	8	Osteoblast_8
Rpl37a	2.24E-10	-0.352037432	1	1	3.27E-06	8	Osteoblast_8
Mmp12	2.41E-10	-0.36572179	0.004	0.175	3.50E-06	8	Osteoblast_8
Snhg18	4.37E-10	-0.690196966	0.859	0.95	6.36E-06	8	Osteoblast_8
Sepw1	8.49E-10	0.628252687	0.959	0.675	1.24E-05	8	Osteoblast_8
Nmt1	4.10E-09	-0.655708949	0.216	0.6	5.97E-05	8	Osteoblast_8
Gm10116	4.26E-09	0.638803046	0.929	0.6	6.21E-05	8	Osteoblast_8
Rps21	5.82E-09	-0.419315978	0.996	1	8.47E-05	8	Osteoblast_8
mt-Nd3	6.32E-09	-0.598533963	0.175	0.575	9.21E-05	8	Osteoblast_8

Oaz1	7.76E-09	0.309656293	0.996	1	0.000112995	8	Osteoblast_8
Rpl39	1.29E-08	-0.463278019	0.993	0.975	0.000187942	8	Osteoblast_8
Arpc1b	1.88E-08	0.552047205	0.981	0.9	0.000273479	8	Osteoblast_8
Rpl18a	2.26E-08	0.317323372	1	1	0.000329585	8	Osteoblast_8
Rps27rt	3.08E-08	-0.553021577	0.45	0.775	0.000448505	8	Osteoblast_8
Rpl10	4.06E-08	0.322552094	1	1	0.00059149	8	Osteoblast_8
Malat1	4.77E-08	-0.58901859	1	1	0.000694561	8	Osteoblast_8
S100a10	6.27E-08	0.650228449	0.922	0.7	0.000913548	8	Osteoblast_8
Rpl29	7.11E-08	0.525363274	0.959	0.75	0.001034848	8	Osteoblast_8
Sertad2	1.03E-07	-0.663026289	0.472	0.8	0.001495406	8	Osteoblast_8
Rplp0	1.04E-07	0.386655032	1	0.975	0.001516618	8	Osteoblast_8
Gpc1	2.29E-07	-0.556209157	0.888	0.975	0.003336713	8	Osteoblast_8
Psap	3.62E-07	-0.784922749	0.87	0.925	0.005271638	8	Osteoblast_8
Gm10073	3.95E-07	-0.430965862	0.888	0.975	0.005746831	8	Osteoblast_8
Zfp36	4.13E-07	-0.584564376	0.253	0.625	0.006010814	8	Osteoblast_8
Atp5k	4.45E-07	-0.440763895	0.877	0.975	0.006480434	8	Osteoblast_8
H3f3b	5.36E-07	0.393237134	1	0.95	0.007803028	8	Osteoblast_8
Serpinh1	6.00E-07	-0.325239056	1	1	0.008738068	8	Osteoblast_8
Rps28	1.33E-26	-0.977214825	1	1	1.94E-22	9	Pre-osteoblast_9
Rps29	1.52E-26	-0.757555776	1	1	2.21E-22	9	Pre-osteoblast_9
Rpl41	1.30E-24	-0.546504198	1	1	1.90E-20	9	Pre-osteoblast_9
Smpd3	3.01E-23	-1.605311002	0.822	1	4.38E-19	9	Pre-osteoblast_9
Rpl38	3.12E-22	-0.682190288	1	1	4.55E-18	9	Pre-osteoblast_9
Rps27	6.75E-19	-0.429450061	1	1	9.84E-15	9	Pre-osteoblast_9

Eif2s2	9.37E-19	-0.791426257	0.956	1	1.36E-14	9	Pre-osteoblast_9
Gm10076	1.15E-18	-0.420952686	1	1	1.67E-14	9	Pre-osteoblast_9
Rpl36	2.33E-18	-0.405348605	1	1	3.39E-14	9	Pre-osteoblast_9
Gm26917	3.24E-18	0.700971937	0.987	0.936	4.73E-14	9	Pre-osteoblast_9
Gm8730	1.04E-17	0.738639205	1	1	1.52E-13	9	Pre-osteoblast_9
Snhg9	5.55E-17	-0.76447425	0.129	0.617	8.08E-13	9	Pre-osteoblast_9
Rpl37a	4.80E-16	-0.396842637	1	1	7.00E-12	9	Pre-osteoblast_9
Rps21	4.76E-15	-0.458862935	1	1	6.94E-11	9	Pre-osteoblast_9
Hes1	1.75E-13	-0.915965627	0.538	0.894	2.55E-09	9	Pre-osteoblast_9
RP23-4H17.3	1.88E-13	-0.40979856	0.013	0.298	2.74E-09	9	Pre-osteoblast_9
Rpl37	2.05E-13	-0.311274551	1	1	2.98E-09	9	Pre-osteoblast_9
Nme2	4.35E-12	0.725722892	0.92	0.66	6.33E-08	9	Pre-osteoblast_9
Lmo4	5.07E-12	-0.750399697	0.729	0.936	7.39E-08	9	Pre-osteoblast_9
Sec61g	5.95E-12	-0.405624811	0.996	1	8.66E-08	9	Pre-osteoblast_9
Snhg18	1.03E-11	-0.598478437	0.813	0.936	1.50E-07	9	Pre-osteoblast_9
Egr1	1.05E-11	-0.673361159	0.48	0.915	1.53E-07	9	Pre-osteoblast_9
Ftl1	1.08E-11	0.492086293	1	1	1.57E-07	9	Pre-osteoblast_9
Rps2	1.23E-11	0.357576113	1	1	1.79E-07	9	Pre-osteoblast_9
Atp5k	1.85E-11	-0.624563463	0.889	0.979	2.69E-07	9	Pre-osteoblast_9
Rps27rt	6.04E-11	-0.652190107	0.538	0.809	8.79E-07	9	Pre-osteoblast_9
mt-Nd2	2.00E-10	-0.678347446	0.867	0.957	2.92E-06	9	Pre-osteoblast_9
Sertad2	3.14E-10	-0.664392728	0.502	0.83	4.58E-06	9	Pre-osteoblast_9
Rpl29	4.93E-10	0.541405575	0.951	0.83	7.18E-06	9	Pre-osteoblast_9
Mef2c	7.03E-10	-0.457191232	0.969	1	1.02E-05	9	Pre-osteoblast_9

Apoe	1.55E-09	-0.388011881	0.08	0.404	2.26E-05	9	Pre-osteoblast_9
Rpl15	1.59E-09	0.367797617	0.996	1	2.32E-05	9	Pre-osteoblast_9
Rps26	2.44E-09	-0.260125155	1	1	3.55E-05	9	Pre-osteoblast_9
Rplp0	3.72E-09	0.34930892	1	1	5.42E-05	9	Pre-osteoblast_9
Timp1	6.91E-09	0.516783965	1	1	0.000100589	9	Pre-osteoblast_9
Gm10263	2.87E-08	-0.503389009	0.084	0.383	0.000417453	9	Pre-osteoblast_9
2010107E04Rik	1.25E-07	-0.48678751	0.684	0.894	0.001827032	9	Pre-osteoblast_9
Rpl39	1.29E-07	-0.357532694	1	1	0.001877075	9	Pre-osteoblast_9
Gm10073	1.73E-07	-0.446372978	0.911	0.979	0.002514851	9	Pre-osteoblast_9
Gm10116	2.39E-07	0.516641807	0.884	0.617	0.003483417	9	Pre-osteoblast_9
Fos	2.72E-07	-0.616622118	0.778	0.915	0.00396843	9	Pre-osteoblast_9
mt-Cytb	3.00E-07	0.271023103	1	1	0.004369359	9	Pre-osteoblast_9
Fat3	3.64E-07	-0.509135567	0.24	0.553	0.005296511	9	Pre-osteoblast_9
Rtn4	5.36E-07	0.508665762	0.791	0.447	0.007800571	9	Pre-osteoblast_9
Rapgef4	5.71E-07	-0.337481465	0.156	0.468	0.008319803	9	Pre-osteoblast_9
Scd2	6.66E-07	-0.570977777	0.804	0.936	0.009694188	9	Pre-osteoblast_9
Rps28	3.62E-19	-0.924796698	0.963	1	5.27E-15	10	Osteoclast_10
Rps29	1.21E-17	-0.589770927	1	1	1.77E-13	10	Osteoclast_10
Rpl38	2.70E-16	-0.786086173	1	1	3.93E-12	10	Osteoclast_10
Rpl41	1.70E-15	-0.593138891	1	1	2.48E-11	10	Osteoclast_10
Rpl15	8.26E-15	1.145707846	0.963	0.542	1.20E-10	10	Osteoclast_10
Gm8730	1.01E-14	1.238007772	0.981	0.759	1.48E-10	10	Osteoclast_10
mt-Nd2	3.23E-14	-1.110057792	0.741	0.976	4.71E-10	10	Osteoclast_10
Rpl18a	3.48E-13	0.693900189	1	1	5.07E-09	10	Osteoclast_10

Rpl9	6.24E-13	0.739463463	1	0.952	9.09E-09	10	Osteoclast_10
Rpl13	7.82E-13	0.697591596	1	1	1.14E-08	10	Osteoclast_10
Rpl37a	3.06E-12	-0.565304013	1	1	4.46E-08	10	Osteoclast_10
Rpl10	4.64E-12	0.741533909	0.981	0.916	6.76E-08	10	Osteoclast_10
Rplp0	5.27E-12	0.939411353	0.981	0.94	7.68E-08	10	Osteoclast_10
Rpl17	2.29E-11	0.715624016	1	0.855	3.33E-07	10	Osteoclast_10
Gm10260	5.18E-11	0.679779734	1	0.916	7.55E-07	10	Osteoclast_10
Phgdh	7.43E-11	0.990167146	0.463	0.012	1.08E-06	10	Osteoclast_10
Atp5k	1.57E-10	-0.770979974	0.926	0.964	2.29E-06	10	Osteoclast_10
Rps2	2.57E-10	0.670312343	1	0.952	3.74E-06	10	Osteoclast_10
Rps6	8.84E-10	0.650719627	1	0.988	1.29E-05	10	Osteoclast_10
Rps18	1.04E-09	0.615019273	1	0.988	1.52E-05	10	Osteoclast_10
Gm10076	1.57E-09	-0.515031458	1	1	2.29E-05	10	Osteoclast_10
Rps5	1.75E-09	0.561064455	1	0.988	2.56E-05	10	Osteoclast_10
Rpl23a	1.96E-09	0.505075223	1	0.964	2.85E-05	10	Osteoclast_10
Rpl3	2.98E-09	0.759097498	0.963	0.892	4.35E-05	10	Osteoclast_10
Rps7	3.87E-09	0.665259721	1	0.904	5.64E-05	10	Osteoclast_10
Tmsb10	6.23E-09	1.095012028	0.926	0.675	9.07E-05	10	Osteoclast_10
Prtn3	7.63E-09	1.9741284	0.352	0	0.00011107	10	Osteoclast_10
Nmt1	8.73E-09	-1.257931484	0.13	0.59	0.000127121	10	Osteoclast_10
Gm10263	9.60E-09	-1.476896488	0.019	0.482	0.000139815	10	Osteoclast_10
Plac8	1.23E-08	2.50980058	0.37	0.012	0.000179826	10	Osteoclast_10
Psap	1.39E-08	-0.745890915	0.796	0.952	0.000202287	10	Osteoclast_10
Rpl10a	2.52E-08	0.489739579	0.981	0.976	0.000366846	10	Osteoclast_10

Rpl29	3.22E-08	0.894943135	0.611	0.181	0.000468244	10	Osteoclast_10
Rpl32	5.54E-08	0.503888388	1	0.988	0.000806229	10	Osteoclast_10
Rpl19	7.08E-08	0.444216094	1	0.952	0.001030731	10	Osteoclast_10
Rpl36	7.08E-08	-0.482412721	0.981	1	0.001031056	10	Osteoclast_10
Rplp2	8.61E-08	-0.365704806	1	1	0.001253658	10	Osteoclast_10
Lgals1	9.98E-08	0.888230935	0.981	0.867	0.001453823	10	Osteoclast_10
Uba52	1.12E-07	0.609325886	0.944	0.831	0.001631025	10	Osteoclast_10
Srgn	1.33E-07	0.910404713	0.333	0.012	0.001940113	10	Osteoclast_10
Rps21	1.35E-07	-0.546617238	0.963	1	0.001960805	10	Osteoclast_10
Rps19	1.63E-07	0.312439431	1	1	0.00237456	10	Osteoclast_10
Ramp1	1.69E-07	0.882629898	0.333	0.012	0.00246359	10	Osteoclast_10
Rpl26	2.50E-07	0.365673847	1	1	0.003637223	10	Osteoclast_10
Ubb	4.14E-07	0.746097931	0.87	0.614	0.006028722	10	Osteoclast_10
Ccr2	4.32E-07	0.6407151	0.278	0	0.006291456	10	Osteoclast_10
Cd24a	4.52E-07	0.864074222	0.315	0.012	0.006582824	10	Osteoclast_10
S100a4	4.84E-07	-0.842515506	0.444	0.831	0.007043748	10	Osteoclast_10
Psap	2.36E-07	-1.531675274	1	1	0.003433121	11	Osteoclast_11
Rps5	3.01E-07	0.704275723	1	0.971	0.004383624	11	Osteoclast_11
Rps18	4.48E-07	0.750387526	1	0.914	0.006526081	11	Osteoclast_11
Rgs10	4.68E-07	1.089427658	0.875	0.276	0.00681364	11	Osteoclast_11
Rps2	3.63E-12	1.035157061	1	0.823	5.29E-08	12	Osteoclast_12
Rps19	1.98E-10	0.70917071	1	1	2.88E-06	12	Osteoclast_12
Rps14	2.53E-10	0.760116958	1	0.987	3.69E-06	12	Osteoclast_12
Rps5	6.48E-10	0.812576942	1	0.937	9.43E-06	12	Osteoclast_12

Psap	6.75E-10	-1.052768084	0.939	1	9.84E-06	12	Osteoclast_12
Rps16	7.03E-10	0.667620542	1	0.949	1.02E-05	12	Osteoclast_12
Gm8730	1.72E-09	1.044838486	0.97	0.722	2.50E-05	12	Osteoclast_12
Rpl13	2.50E-09	0.793355579	1	0.975	3.65E-05	12	Osteoclast_12
Rps23	9.24E-09	0.722635465	1	0.962	0.000134534	12	Osteoclast_12
Rpl35	9.59E-09	0.662280221	1	1	0.000139732	12	Osteoclast_12
Rps4x	2.02E-08	0.704363268	1	0.962	0.000294435	12	Osteoclast_12
Bgn	7.03E-08	-0.799575358	0.909	0.962	0.001024429	12	Osteoclast_12
Rpl15	8.66E-08	0.963558383	0.879	0.443	0.001261922	12	Osteoclast_12
Rps15a	1.51E-07	0.711047824	0.97	0.81	0.002206361	12	Osteoclast_12
Rps18	1.55E-07	0.698758229	1	0.962	0.002259477	12	Osteoclast_12
Ptrf	1.71E-07	-1.12521022	0.424	0.873	0.002495629	12	Osteoclast_12
Acta2	1.95E-07	-1.004287052	0.758	0.962	0.002839643	12	Osteoclast_12
Gm10260	2.71E-07	0.772122972	1	0.823	0.00394221	12	Osteoclast_12
Rpl18a	3.52E-07	0.654379612	1	0.987	0.005129459	12	Osteoclast_12
Rpl11	4.22E-07	0.686077057	1	0.835	0.006145945	12	Osteoclast_12
Calu	4.83E-07	-1.036004391	0.667	0.873	0.007028389	12	Osteoclast_12

Supplementary Table 2: Mouse real-time RT-PCR (qPCR) primers

Name	5' primer	3' primer
Adiponectin	TGTTCCTCTTAATCCTGCCCA	CCAACCTGCACAAGTTCCCTT
Alp	CCAACTCTTTTGTGCCAGAGA	GGCTACATTGGTGTTGAGCTTTT
aP2	GGGGCCAGGCTTCTATTCC	GGAGCTGGGTTAGGTATGGG
Axin2	TGACTCTCCTTCCAGATCCCA	TGCCCACACTAGGCTGACA
Bmp4	TTCCTGGTAACCGAATGCTGA	CCTGAATCTCGGCGACTTTTT
C/EBPα	CAAGAACAGCAACGAGTACCG	GTCACTGGTCAACTCCAGCAC
C/EBPβ	CAAGCTGAGCGACGAGTACA	CAGCTGCTCCACCTTCTTCT

CatK	AATACGTGCAGCAGAACGGAGGC	CTCGTTCCCCACAGGAATCTCTCTGTAC
Col1a1	GCTCCTCTTAGGGGCCACT	CCACGTCTCACCATTGGGG
Csfr1	CCTCCTCTGGTCCTGCTGCTGG	GCTCACACATCGCAGGGTCACC
Cx43	ACAGCGGTTGAGTCAGCTTG	GAGAGATGGGGAAGGACTTGT
Dkk1	CATGAGGCACGCTATGTGCTG	GCGGCGTTGTGGTCATTACC
Gapdh	CAGTGCCAGCCTCGTCCCGTAGA	CTGCAAATGGCAGCCCTGGTGAC
Kindlin-2	CTCTGACCGAGTCTTCAAGGC	ATGAGCGTCGTAAGTAGGGGT
Lef1	TGTTTATCCCATCACGGGTGG	CATGGAAGTGTCGCCTGACAG
Lrp5	GGGTCCACAAGGTCAAGGC	GCACCCTCCATTTCCATCC
Lrp6	GCCCACTACTCCCTGAATGCTG	TGTGGATAGGAAGGATGATGTCAG
Mmp9	TGCCCTGGAACTCACACGACATCTTC	TCCACCTGGTTCACCTCATGGTCC
Nfatc1	CCCCATCCGCCAGGCTACA	GGTTGTCTGCACTGAGCCAACTCC
Ocn	AGGGAGGATCAAGTCCCG	GAACAGACTCCGGCGCTA
Osx	ATGGCGTCCTCTCTGCTTG	TGAAAGGTCAGCGTATGGCTT
Pparγ2	TCGCTGATGCACTGCCTATG	GAGAGGTCCACAGAGCTGATT
Pref-1	GACCCACCCTGTGACCCC	CAGGCAGCTCGTGCACCCC
Pu.1	CTCCAGGTGTACCCCCCAGAGG	CTCCAGGGCGGGGCTGTC
Rank	GGACGGTGTTGCAGCAGAT	GCAGTCTGAGTTCCAGTGGTA
Rankl	CAGCATCGCTCTGTTCCTGTA	CTGCGTTTTCATGGAGTCTCA
Runx2	AACGATCTGAGATTTGTGGGC	CCTGCGTGGGATTTCTTGGTT
Smad6	GCAACCCCTACCACTTCAGC	GTGGCTTGTACTGGTCAGGAG
Sclerostin	AGCCTTCAGGAATGATGCCAC	CTTTGGCGTCATAGGGATGGT
Trap	CACTCCCACCCTGAGATTTGT	CATCGTCTGCACGGTTCTG

Supplementary Table S3: Antibody information

Antibody	Company	Catalog #	Application/Dilution
β-Actin	Sigma	A2228	WB (1:4000)
Active-β-catenin	Millipore	05-665	WB (1:2000)
β-catenin	CST	9562S	WB (1:1000), IHC (1:1000)
Kindlin-2	Abcam	Ab74030	WB (1:1000), IHC (1:700), IF (1:200)
Nfact1	Santa Cruz	sc-7294	WB (1:500), IHC (1:500)
Osx	Abcam	ab22552	WB (1:1000), IHC (1:2000)
Phalloidin-488	Invitrogen™	A12379	IF (1:200)
Runx2	Abcam	ab76956	WB (1:1000）
Rankl	Santa Cruz	sc-52950	IHC (1:500)
Anti-Rankl neutralizing Ab	R&D	AF462	40 ng/ml

Sclerostin	Abcam	Ab63097	IHC (1:200)
Sclerostin	Novus	NBP1-50549	WB (1:500)

Supplementary Table 4. Mutation primers for CRISPR/Cas9 deletion of mouse Kindlin-2.

K2-sgT	5'-CACCGCTGCTACGCGGACGGGACGT-3'
K2-sgB	5'-AAACACGTCCCGTCCGCGTAGCAGC-3'

Supplementary Table 5: Sequencing primers for CRISPR/Cas9 deletion of mouse Kindlin-2

K2 (Forward)	5'-AGTGACGCTGACGAGAGCCATTTCC-3'
K2 (Reverse)	5'-GCGCAGCACA CGCCAGGAGCTAGGA-3'
